# Single cell map of the adult female mouse urethra reveals epithelial and stromal macrophages with distinct functional identities

**DOI:** 10.1016/j.mucimm.2025.09.001

**Published:** 2025-09-02

**Authors:** Jasmine, Mrinal Samtiya, Richelle Rodrigues, Aparna Mandal, T.T. Kavya, Anubhuti Anushree, John T. Lafin, Chad M. Vezina, Douglas W. Strand, Diya Binoy Joseph

**Affiliations:** a Institute for Stem Cell Science and Regenerative Medicine (BRIC-inStem), GKVK-Post, Bellary Road, Bengaluru 560065, India; b Department of Urology, UT Southwestern Medical Center, 5323 Harry Hines Blvd, Dallas, TX 75390, United States; c Department of Comparative Biosciences, University of Wisconsin-Madison, Veterinary Medicine Building, 2015 Linden Dr, Madison, WI 53706, United States; d Regional Centre for Biotechnology, Faridabad, Haryana 121001, India

**Keywords:** Macrophage, Urethra, Single cell RNA-sequencing, Epithelium, Chemokine

## Abstract

Epithelial linings at mucosal sites act in concert with resident immune cells to direct host defense. The epithelial lining of the urethra is an understudied mucosal barrier with emerging roles in antimicrobial defense during urinary tract infections. Here, we present a comprehensive cellular atlas of the adult female mouse urethra, focusing on epithelial and resident immune cells. Single cell RNA-sequencing revealed two distinct macrophage populations compartmentalized within the epithelium and stroma. Epithelial-associated macrophages display a highly dendritic morphology and populate the urethral lining in increasing numbers over the course of development. Epithelial-associated macrophages express *Cx3cr1*, MHCII genes, *Cd74* and *Aif1/Iba-1*, representing an activated macrophage type (Mac-Activated) enriched for pathways involved in antigen presentation and the inflammatory response. In contrast, stromal macrophages express the scavenging receptors *Mrc1/Cd206, Lyve1, Cd163* and *Mgl2/Cd301b* and display a signature enriched for endocytic function (Mac-Endocytic), vasculature development and tissue homeostasis. We identified epithelial cells in the urethral lining and associated glands expressing the monocyte chemoattractant genes *Cx3cl1* and *Cxcl17*. Chemoattractant gene expression in the urethral epithelium follows a proximal–distal gradient which correlates with the increasing density of epithelial-associated macrophages expressing the receptor *Cx3cr1* along the proximal–distal axis of the urethra. The study delineates spatially compartmentalized macrophage subsets in the urethra and implicates epithelial-derived chemokines in the establishment of macrophage positioning and functional specialization along the urethral axis.

## Introduction

Mucosal linings are contact sites for host-pathogen interactions. The interplay between mucosal epithelial linings and resident immune cells play a major role in tissue homeostasis and host defense. Compared to other mucosal sites, the lower urinary tract, and in particular the urethra, has been understudied as a mucosal barrier. The urinary tract is susceptible to infections, specifically caused by uropathogenic bacteria from the gut ascending up the urethra to infect the bladder. Recent work has started to uncover the multi-faceted immune defense arsenal of the urethra. The epithelium lining the adult male prostatic urethra was shown to express a plethora of antimicrobial and immunomodulatory genes^1,2^. Specialized neuroendocrine cells in the urethral lining participate in defense against urinary pathogens by regulating muscle contraction to expel ascending pathogens^3^. Anatomical differences in urethral length make females more susceptible to urinary tract infections. Despite being the first mucosal lining in the urinary tract that comes in contact with pathogens, epithelial sub-types and resident immune cells in the female urethra have not been investigated.

Tissue resident macrophages are ubiquitous sentinels and mediators of homeostasis at mucosal sites. During early development, waves of hematopoiesis push embryonic macrophages to populate developing organs^4–6^. Subsequent definitive hematopoiesis in the bone marrow further contributes to tissue resident macrophage populations through monocyte progenitors^7,8^. Tissue resident macrophage function and gene expression is broadly conserved across the body, although macrophages can possess different characteristics based on the tissue niche they occupy^9,10^. Langerhans cells of the skin^11^, Kupffer cells of the liver^12^ and alveolar macrophages^13^ exhibit specialized functions influenced by the local tissue microenvironment. In organs like the gut^14^ and skin^15^, resident macrophages send out dendritic projections into the epithelial lining which allow them to sample antigens from the outside environment. Non-immune functions of macrophages, specific to the niches they occupy, have been steadily gaining prominence. For instance, cardiac macrophages participate in electric conductance in the heart^16^ while gut macrophages provide local signals that maintain the intestinal stem cell niche^17^.

Signals from specialized tissue compartments at mucosal sites can influence macrophage localization and functional diversification^18,19^. Based on proximity to the epithelial compartment, two distinct macrophage pools can be found in organs like the lung^13^, bladder^20^ and prostate^21^, suggesting that macrophage segregation is influenced byniche specific gene expression, creating a unique organization based on tissue type. Although the lower urinary tract is lined by a continuous epithelium^22^, previous work from our group identified compartmentalized gene expression in the bladder and urethral epithelium during embryonic development. We found that expression of chemokines and barrier function genes increases specifically in the urethral lining over the course of development^23^. In this study, we present a single cell atlas of the adult female mouse urethra which reveals a unique positioning of macrophages in the urethral lining, potentially influenced by compartmentalized epithelial chemokine gradients.

Our work reveals the diversity of resident immune cell populations in the female urethra and identifies two spatially separated and transcriptionally distinct macrophage populations. One population of epithelial-associated macrophages displays a large cell perimeter and sends out dendrites in between epithelial cells. The other macrophage population resides in the stromal compartment and expresses gene signatures for scavenging, vascular development and tissue homeostasis. We also identify two secretory epithelial cell types in the adult female mouse urethra which express chemoattractant genes. Expression of chemoattractant genes in the urethral lining increases as we move closer to the outside of the body and correlates with the density of epithelial-associated macrophages which also increases towards the urethral opening. This study sheds light on an understudied mucosal barrier that is at the frontline of the response to urinary pathogens, identifying novel macrophage populations and epithelial gene signatures that can influence immunity.

## Materials and methods

### Animal husbandry and usage

Procedures on animals were approved by the inStem Institutional Animal Ethics Committee under protocols INS-IAE-2022/01(M2) and INS-IAE-2023/14(N). Animals were housed in a specific pathogen free facility with routine health monitoring. Animals were provided adlibitum chow (Safe-D131), autoclaved water and housed in individually ventilated cages. Mice were subjected to a 12-hour light/dark cycle. Wildtype CD-1 mice (Charles River, USA # Crl:022) were obtained from the Animal Care and Resource Center at the Bangalore Life Sciences Cluster (BLiSC). Timed mating was performed with mice older than 7 weeks and noon of the day of vaginal plug detection was deemed to be embryonic day 0.5. For embryo collection, pregnant female mice at various stages of gestation were euthanized by approved methods and embryonic tissues were removed for fixation. Male and female embryos were collected at embryonic day (E) 14.5 and E16.5 stages. Only female mouse tissues were collected at E18.5, postnatal day (P) 0, 5 and 20 days. 8–10-week-old CD-1 male and female mice were used for adult stages. Pgk1-cre mice (B6.C-Tg(Pgk1-cre)1Lni/CrsJ, Strain #020811) and Csf1r^flox^ (B6.Cg-Csf1rtm1.2Jwp/J, Strain #021212) mice were obtained from The Jackson Laboratory and shared by the BLiSC Animal Care and Resource Center. Macrophage depletion mutants were generated by breeding Pkg1-cre mice with Csf1r^flox^ mice to achieve cre expression and Csf1r^flox^ allele homozygosity. Control mice did not carry the cre allele and had one allele of Csf1r^flox^. Timed breeding was performed to obtain control and mutant embryos at E18.5.

### Immunostaining of paraffin or OCT sections

For paraffin processing, whole embryos or dissected lower urinary tracts from embryos as well as postnatal and adult animals were fixed in 4 % paraformaldehyde in PBS or 10 % neutral buffered formalin overnight. Tissues were washed in 1x PBS and processed through a series of ethanol washes culminating in clearing with xylene before incubation in paraffin wax as previously described^23^. 5-μm sections were placed on Superfrost plus charged slides. Sections were heated at 60C, deparaffinized with xylene and rehydrated through a series of ethanol washes. Antigen retrieval was performed in a 0.01 M citric acid antigen retrieval solution by microwaving at high power for 20 min. For fresh-frozen sections, dissected tissues were washed in ice-cold PBS and embedded in optimal cutting temperature (OCT) media before cutting sections. 10-μm frozen sections on Superfrost plus slides were thawed and fixed in 10 % neutral buffered formalin for 15 mins, washed in 1x PBS and permeabilized in 0.25 % Triton X-100 in PBS for 5 mins. Slides were blocked in blocking buffer (1x Tris-buffered Saline, 0.2 mM sodium azide, 0.1 % (v/v) Tween-20, 0.1 % (w/v) Bovine serum albumin Fraction V, 5 % Horse serum) for 1 h at room temperature. Primary antibodies diluted in blocking buffer were applied to the slides and incubated overnight at 4C with gentle rocking. Slides were washed with 1x PBS and secondary antibodies were diluted in blocking buffer and incubated with tissue sections for 1 h at room temperature. Following washes, 300 nM of 4′,6-diamidino-2-phenylindole (DAPI) was applied to the slides for 5 mins. After final washes with 1x PBS, slides were mounted in a glycerol based mounting media containing the antifade reagent n-propyl gallate. Images were obtained using Zeiss Axio Observer 7. Primary and secondary antibodies used in this study are listed in the Key resources table.

### Immune cell image analysis

Lower urinary tract sections from embryos or mice across independent litters were labeled with antibodies to immune cell markers and DAPI to label nuclei. Image analysis was performed using ImageJ (Version 1.54p) and statistical analysis was performed using R statistics software. Epithelial and stromal compartments were separately markedas regions of interest (ROIs) and DAPI stained nuclei were segmented using Adjust threshold, split using Binary > Watershed and counted using Analyze particles. Immune cells were counted using the Cell-Counter plugin and represented as a percentage of nuclei in the epithelial or stromal compartments. Statistical analysis was performed with R statistics software. Bartlett’s test was performed to test equality of variance between the groups. For comparison between two groups, unpaired, two-tailed student’s *t*-test or Mann-Whitney *U* test was performed with significance level cut-off of 0.05 to reject null hypothesis. For macrophage counts in the urethra along the proximal–distal axis, the urethral length from bladder neck to the distal-most portion of the urethra on the section was measured and divided into three equal parts by length. Epithelial and stromal compartments in each third were marked as separate ROIs and DAPI stained nuclei were segmented using Adjust threshold, split using Binary > Watershed and counted using Analyze particles. Macrophages labeled by F4/80 were counted using the CellCounter plugin and represented as a percentage of nuclei. Bartlett’s test was performed to test equality of variance between groups. One-way ANOVA followed by Tukey’s HSD or Kruskal-Wallis test followed by Dunn’s test was performed for more than 2 groups with significance level cut-off of 0.05 to reject null hypothesis. For cell perimeter analysis, fluorescently labeled immune cells were traced using the free hand selection tool in ImageJ to mark perimeters. Cell perimeter for each individual cell was obtained in microns using Analyze > Measure. Dendrites on each cell were counted using the cell counter plugin. Comparisons were made between values obtained from immune cells in the epithelial and stromal compartments. Bartlett’s test was performed to test equality of variance between the groups. For comparison between two groups, unpaired, two-tailed student’s *t*-test or Mann-Whitney *U* test was performed with significance level cut-off of 0.05 to reject null hypothesis.

### Epithelial-stromal separation

This method was modified from a published study on endometrium^24^. Dissected adult female urethras from CD-1 mice were cut into two crosswise and placed in 1 % Trypsin solution in 1x HBSS for 45 min at 4C on a tube rotator. Following this, MgCl2 (10 mM) and DNase I (10 μg/ml) were added to the trypsin solution and the tissues were incubated for a further 45 mins at 37C. Digested tissues were incubated in 10 % sheep serum in 1x HBSS for 5 mins. Tissues were transferred to ice-cold 1x HBSS and squeezed between forceps to separate the epithelium from the stroma.

### Wholemount immunostaining

Separated urethral epithelial sheets from adult female mice were fixed for 20 mins in 10 % neutral buffered formalin at 4C. Following washes in 1x PBS, tissues were permeabilized in 1x PBS + 1 % Triton X-100 for 2 h at 4C with rocking. Tissues were transferred to horse blocking serum containing 1 % of Triton X-100 and incubated overnight at 4C on a rocker. For primary antibody incubations, tissues were transferred to horse blocking buffer with 1 % Triton X-100 containing primary antibodies and incubated for 48–72 h at 4C on a rocker. Following primary antibody incubation, tissues were washed with 1x PBS (3x30 mins) at room temperature. For secondary antibody incubation, tissues were transferred to horse blocking buffer with 1 % Triton X-100 and fluorescently labeled secondary antibodies. Tissues were incubated in the dark for 4–6 h at room temperature on a rocker. Following washes in 1x PBS, tissues were transferred to anti-fade mounting solution and incubated until sufficiently clear. Tissues were mounted and imaged using Zeiss Axio Observer 7 with Apotome 3 optical sectioning. Separated urethral epithelial sheets from adult wildtype CD-1 (n = 2) and genotype inappropriate mice (n = 2) from our colony were used for this in a bid to adhere to the 3Rs of animal usage. Primary and secondary antibody details are in the Key resources table.

### Single cell RNA-sequencing

Dissected adult female mouse urethras pooled from 6-8 CD-1 female mice were chopped into small pieces (0.5–1 mm) and added to Accutase solution (Sigma Aldrich, SCR005) containing 10 μM of Y-27632 and 1 mg/ml DNaseI. Tissues were incubated for 1.5 h on a tube rotator at 37C. For epithelial and stromal separations, tissues pieces were incubated in Accutase solution containing 10 μM of Y-27632 and 100 μg/ml DNaseI on a tube rotator at 37C for 1 h. For comparing transcriptional changes upon lipopolysaccharide (LPS) stimulation, mice were instilled with 100 μl of sterile 1x PBS or 100 μl of sterile 1x PBS containing 1 mg/ml LPS (Sigma Aldrich, L2630-10MG). Instillation was performed by catheterizing the mice with a length of polyethylene tubing (0.28 x 0.61 mm) threaded onto a 30-gauge needle attached to an insulin syringe. Mice were euthanized 3 h post-instillation and urethras from 3 mice per group were pooled for the PBS and LPS conditions. Tissues were incubated in Accutase solution for 1.5 h on a tube rotator at 37C. After digestion, tissue pieces were spun down by centrifugation and resuspended in a wash buffer containing 1x PBS and 1 % bovine serum albumin. Tissues were further broken up by trituration and passing through a needle. Cells were filtered through a 30-μm filter and red blood cell lysis was performed. Cells were resuspended in wash buffer, counted and resuspended to a density of 800–1000 cells/μl for single cell RNA-sequencing. A target of 10,000 cells per sample were loaded on to the 10x Chromium controller for cell partitioning. Libraries were prepared according to manufacturer’s instructions for the Chromium Next GEM Single Cell 3′ Kit v3.1 (10x Genomics, 1000269). Libraries were pooled for sequencing using the Illumina SP kit (Illumina, 20028401) on NovaSeq6000. Read-depth of 40,000–60,000 reads per cell was targeted. Base calling was performed using Illumina bcl2fastq. Barcoding processing and single cell 3′ counting were performed using cellranger-8.0.1. Cell clustering was performed using Seurat 5.1.0^25^. Cells were filtered by gene number and percent mitochondrial genes for downstream analysis. Feature plots, dot plots and violin plots for marker genes were generated using Seurat. Differential expression between clusters was performed using the FindMarkers and FindAllMarkers function in Seurat. Gene ontology and KEGG pathway analysis were performed using WebGestalt 2024^26^ against Mus musculus genome protein coding genes. Published datasets of single cell RNA-sequencing from whole mouse bladders (GSE129845, sample GSM3723360)^27^, mouse bladder immune cells (GSE149571, sample GSM45049690)^28^ and male mouse prostatic urethra (GSE1458650)^1^ were analyzed using Seurat 5.1.0. Where applicable, published bladder datasets and female urethra single cell RNA-sequencing data from this manuscript were integrated using the IntegrateData function in Seurat before clustering. R scripts for all analyses in Seurat are provided in Supplementary File 1.

### RNAscope

RNAscope assays for detection of mRNA transcripts were performed using RNAScope 2.5 HD Reagent Kit Brown (ACD, 322371) or the RNAscope Multiplex Fluorescent Reagent V2 kit (ACD, 323270) following manufacturer instructions. Tissues were fixed for 16–24 h in 10 % neutral buffered formalin at room temperature. 5-μm paraffin sections were probed with RNAscope® Probe-Mm-*Csf1r* (ACD, 428191), Probe-Mm-*Cx3cl1* (ACD, 426211), Probe-Mm-*Cx3cr1* (ACD, 314221) or Probe-Mm-*Cxcl17* (ACD, 519621). Standard pre-treatment steps were performed with 15 mins of antigen retrieval by steaming and 30 mins of protease treatment. After development of the stain with colorimetric dye DAB or the TSA Vivid Fluorophore 570, tissues were counterstained with hematoxylin or DAPI and mounted. Images were obtained using Zeiss Axio Observer 7.

### Flow cytometry

Dissected adult female mouse urethras and bladders pooled from 4-5 CD-1 female mice per sample were chopped into small pieces (0.5–1 mm) and added to Accutase solution containing 10 μM of Y-27632 and 1 mg/ml DNaseI. Tissues were incubated for 1.5 h on a tube rotator at 37C. Tissue pieces were spun down by centrifugation and resuspended in a wash buffer containing 1x PBS and 1 % bovine serum albumin. Tissues were further broken up by trituration and by passing through a needle. Cells were filtered through a 30-μm filter and red blood cell lysis was performed. Cells were incubated with Mouse TruStain FcX (Biolegend, 101320) and Ghost Dye™ Violet 450 (Tonbo, 13–0863-T100). Following washes in 1x PBS, cells were incubated with fluorescently conjugated primary antibodies. Cells were washed and filtered through a 70-μm filter and data was collected using an Attune NxT flow cytometer. Data was analyzed using FCS Express 7 and percentages of CD45 + cells with F4/80+/CD206 high and F4/80+/CD206 low expression were compared between bladder and urethra samples. Bartlett’s test was performed to test equality of variance between groups. One-way ANOVA followed by Tukey’s HSD was performed with significance level cut-off of 0.05 to reject null hypothesis. Antibody details are provided in the Key resources table.

### Xenium spatial transcriptomics

Xenium assay was performed on 5-μm paraffin sections of female C57BL6/J mouse lower urinary tracts under UT Southwestern IACUC approval number 2023–103391. The Mouse Tissue Atlassing Panel (10x Genomics, 1000627) was used with 100 additional custom probes. Custom probe details are provided in the Key resources table.

## Results

### Macrophages infiltrate the urethral epithelium over the course of development

**Fig. 1:**
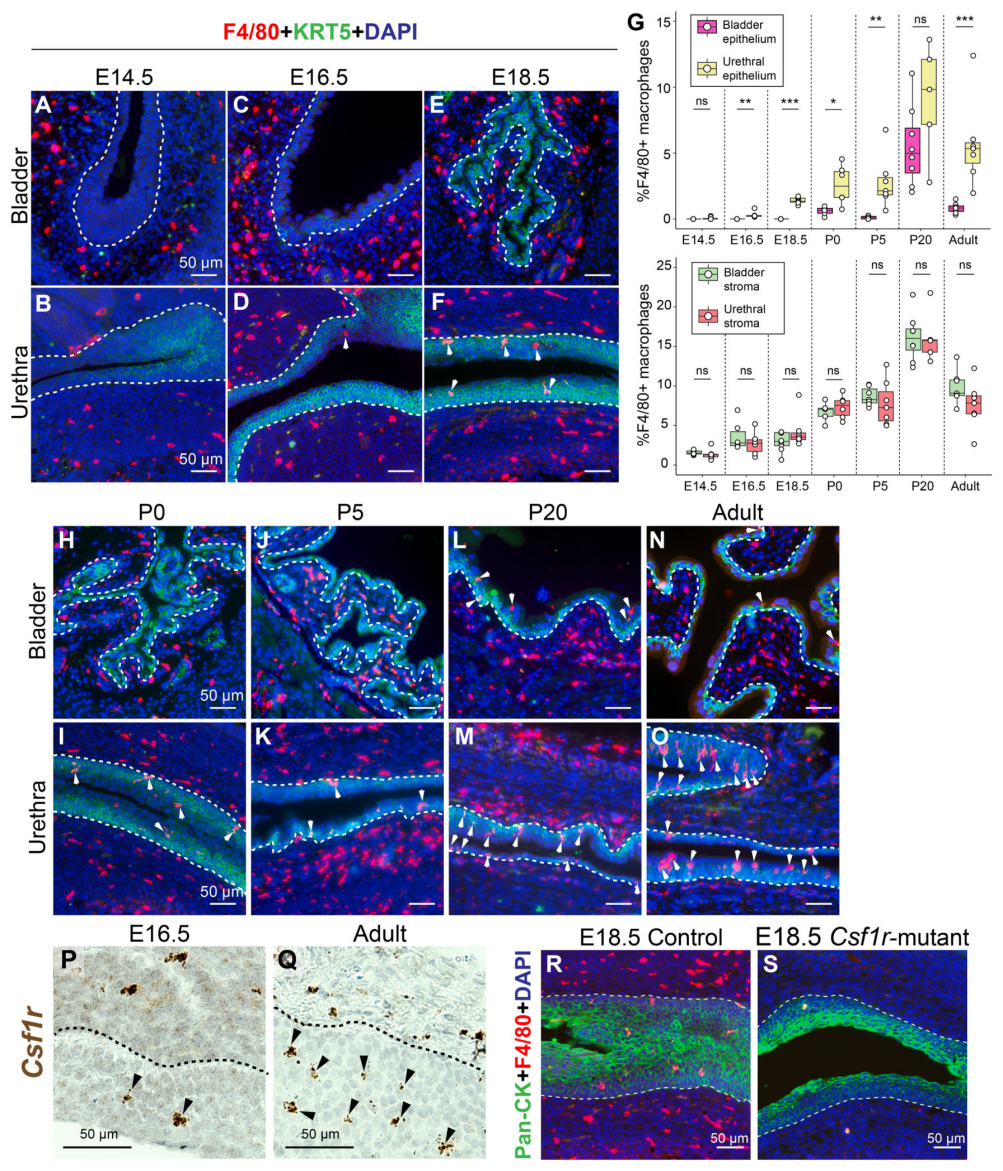
Macrophage kinetics in the developing and postnatal urethra vs bladder. Tissue sections of the bladder and urethra from (A-B) E14.5 mice, (C-D) E16.5 mice and (E-F) E18.5 female mice were labeled with antibodies to the macrophage marker F4/80 (in red) and the basal epithelial marker KRT5 (in green). (G) Quantification of F4/80 + macrophages in the epithelial and stromal compartments of the bladder and urethra across different stages. Male and female mice were assessed at E14.5 and E16.5 while only female mice were assessed from E18.5 to Adult.Tissue sections of the bladder and urethra from female mice at (H-I) P0, (J-K) P5, (L-M) P20 and (N-O) adult stages were labeled with antibodies to the macrophage marker F4/80 (in red) and the basal epithelial marker KRT5 (in green). Images and data are representative of n = 5–8 mice/group from at least n = 3 independent litters. Tissue sections from (P) E16.5 mouse urethra and (Q) adult female mouse urethra labeled with probes against the macrophage gene *Csf1r*. Tissue sections counterstained with hematoxylin. Images are representative of n = 3 mice/group. Tissue sections of E18.5 mouse urethras from (R) control and (S) *Csf1r* depleted mice labeled with antibodies against F4/80 (in red) and Pan-keratin (in green). Images are representative of n = 3 mice/group. Nuclei labeled by DAPI in blue. Scale bar represents 50 μm. Arrowheads indicate epithelial-associated macrophages in the urethra. Dotted lines represent the basement membrane. Abbreviations: E: embryonic day, P: postnatal day, ns: non-significant. * p < 0.05, ** p < 0.01, *** p < 0.001 from Student’s *t*-test or Wilcoxon Rank sum test. (For interpretation of the references to colour in this figure legend, the reader is referred to the web version of this article.)

Macrophages are the earliest immune cells to populate tissue sites during development. We assessed the infiltration of F4/80 + macrophages into the fetal lower urinary tract. Macrophages are interspersed in the mesenchymal compartments of the developing bladder and urethra at embryonic day (E) 14.5 (Fig. 1 A-B) and 16.5 (Fig. 1 C-D, Fig. S1 A). Due to sexual dimorphism in the urethra, we focused on the female lower urinary tract for subsequent stages. From E16.5, we started to observe macrophages in the epithelial lining of the urethra. At E18.5, macrophages infiltrate the epithelial lining of the female urethra in more numbers but are absent from the bladder lining (Fig. 1 E-G). We then assessed macrophage infiltration into the female urethra and bladder during postnatal stages. Macrophage infiltration into the urethral epithelium increases during postnatal development. At Postnatal day (P) 0 and P5, very few macrophages are found in the bladder epithelium but numbers in the urethral epithelium are rising (Fig. 1 G, H-K). Macrophage infiltration into the urethral epithelium peaks at postnatal day P20 and at this stage, there is a spike in macrophages present in the bladder epithelium as well (Fig. 1 G, L-M). The spike in epithelial macrophages at P20 coincides with the weaning stage in mice. In mouse intestines, it has been shown that monocyte derived macrophages appear at the time of weaning, diluting the embryonic macrophage pool and establishing the adult colonic macrophage population^29^. The spike in epithelial-associated macrophages in the lower urinary tract around weaning could be a result of monocyte infiltration at this stage although further research is required to establish a causal relationship. Macrophages are fewer in the adult female urethra (8 weeks) compared to P20 and show a higher density in the urethral epithelium compared to the bladder epithelium (Fig. 1 G, N-O, Fig. S1 A).

The density of stromal macrophages is comparable between the bladder and urethra at all stages analyzed. Similar to the epithelial compartment, we see a spike in macrophage density in the stromal compartment at P20 which subsides by the adult stage (Fig. 1 G). Epithelial-associated macrophages are also present in the adult male mouse urethral epithelium. We observe higher density of macrophages in the male urethral epithelium compared to the bladder epithelium while there are no differences in macrophage density in the stromal compartments of the bladder and urethra (Fig. S1 B-F).

Macrophages infiltrating the urethral epithelium and residing in the stroma during the embryonic and adult stages express the Colony stimulating factor-1 receptor (*Csf1r*) (Fig. 1 P-Q) and depletion of this gene completely ablates macrophages in the developing lower urinary tract including the urethral epithelium (Fig. 1 R-S). Overall, our findings reveal macrophage kinetics in the lower urinary tract over the course of embryonic and postnatal development and show robust macrophage infiltration into the urethral epithelium.

### Single cell analysis reveals heterogeneity of the resident immune niche in the adult female mouse urethra

**Fig. 2:**
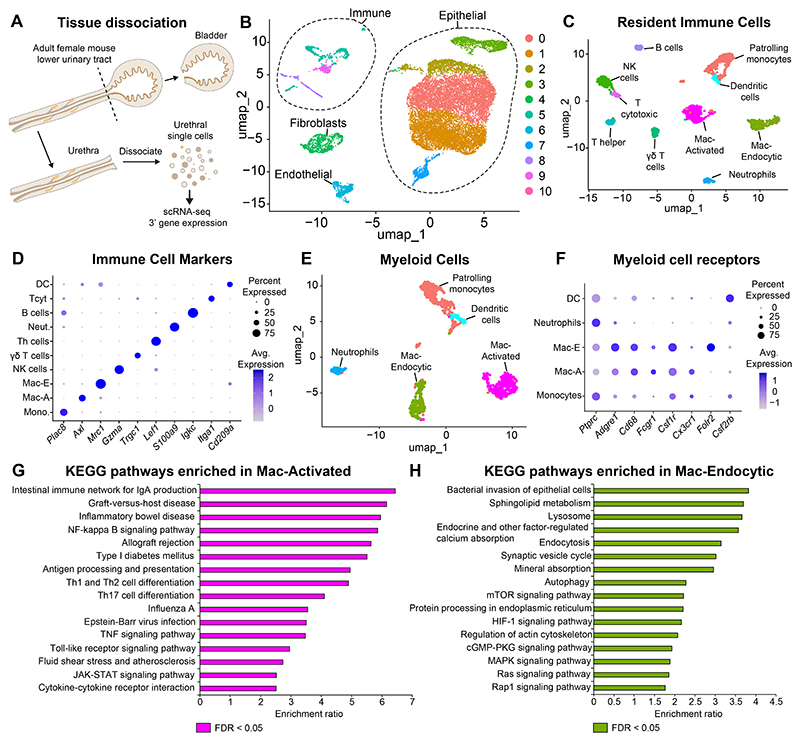
Immune cell populations in the adult female mouse urethra. (A) Schematic of tissue dissociation and single cell isolation of the adult female mouse urethra. (B) UMAP depicting major cell clusters from the female mouse urethra post filtering for stressed cells. (C) UMAP depicting clusters of immune cells from the adult female mouse urethra. (D) Dot plot showing marker genes for each immune cell population. (E) UMAP depicting myeloid cell populations. (F) Dot plot showing expression of key receptor genes in the myeloid cell clusters. (G) KEGG pathways enriched in the Mac-A cluster compared to the Mac-E cluster. (H) KEGG pathways enriched in the Mac-E cluster compared to the Mac-A cluster. Abbreviations: scRNA-seq: Single cell RNA-sequencing, DC: Dendritic cells, NK: Natural Killer cells, Neut.-:Neutrophils, Mac-E: Macrophage-Endocytic, Mac-A: Macrophage-Activated, Mono.: Monocytes, FDR: False Discovery Rate.

Bacteria ascend up the urethra to infect the bladder but the resident immune cell composition of the urethra has not been investigated. Given the anatomical differences between the male and female urethra and the increased susceptibility to urinary tract infections in females, we focused on the adult female mouse urethra for this study. To assess resident immune cells in the adult female mouse urethra, we dissected and pooled urethras from female CD-1 mice and performed single cell digestion (Fig. 2 A). Single cell RNA-sequencing was performed to assess gene expression (Table S1). Cells were filtered by quality control metrics and stress gene expression (Fig. S2 A-B, Table S2). Clusters corresponding to immune cells (*Ptprc* expression) and epithelial cells (*Epcam* expression) were identified (Fig. S2 C-E). Immune cells were sub-setted and re-clustered (Fig. 2 C, Table S3). The major myeloid immune cell clusters identified are patrolling monocytes (*Plac8, Adgre4, Ace*) and two macrophage clusters both expressing canonical macrophage markers (*Fcgr1, Adgre1, C1qa* and *C1qb*). One cluster, Mac-Activated (Mac-A), expressed the genes *Cd86, H2-Ab1, Axl* and *Cd74* involved in inflammatory processes. The other cluster, Mac-Endocytic (Mac-E), expressed genes involved in scavenging and endocytosis like *Mrc1, Cd163, Lyve1* and *Mgl2*. Additionally, dendritic cells (*Cd209a/DC-SIGN*) and neutrophils (*S100a9*) were identified. Among lymphoid clusters, we identified T helper cells (*Zeb1, Lef1*), Gamma delta T cells (*Trgc1, Trgc2*), NK cells (*Gzma, Klre1, Nkg7*), T cytotoxic cells (*Gzmc, Tnfrsf9, Itga1*) and B cells (*Igkc, Iglc2*) (Fig. 2 D, Table S3).

We generated a subset of the myeloid cell clusters and re-clustered them. We observed a monocyte cluster, two macrophage clusters, a dendritic cell cluster and a neutrophil cluster (Fig. 2 E, Table S4). Both macrophage clusters express *Adgre1* or F4/80, *Cd68, Cd64*/*Fcgr1* and *Csf1r* (Fig. 2 F, Fig. S2 F). Additionally, the two macrophage clusters express the complement genes *C1qa* and *C1qb* (Fig. S2 F). While the macrophage clusters express the receptor for colony-stimulating factor-1, *Csf1r*, the dendritic cell cluster expresses the receptor for colony-stimulating factor-2, *Csf2rb*. The first macrophage cluster, Mac-A, shows higher expression of the chemokine receptor *Cx3cr1* compared to the Mac-E cluster (Fig. 2 F). In addition, pathways for antigen processing and presentation, NF-kappa B signaling pathway, TNF signaling pathway, JAK-STAT signaling pathway and T cell differentiation are enriched in the Mac-A cluster (Fig. 2 F-G, Table S5, Table S6). The Mac-E cluster is characterized by the expression of *Mrc1* and other receptors involved in endocytosis like the folate receptor *Folr2*, which mediates folate uptake into cells ^30^. Pathways for endocytosis, autophagy, MAPK signaling, sphingolipid metabolism and HIF-1 signaling are enriched in the Mac-E cluster (Fig. 2 F, H, Table S5, Table S7).

Upon identifying Mac-A and Mac-E cells in the female mouse urethra, we assessed whether similar macrophage subtypes are present in the male urethra. Using a previously published dataset^1^ of single cell transcriptomes from the male mouse prostatic urethra, we found Mac-A (*Cx3cr1, Cd74, Ms4a7*) and Mac-E (*Pf4, Mrc1, Cd163*) cells in the male urethra as well (Fig. S3, Table S8).

Our assessment of resident immune cells in the urethra reveals two transcriptionally distinct macrophage populations, Mac-A with inflammatory gene expression and Mac-E with expression of scavenging receptors.

### Identification of epithelial and stromal macrophages in the urethra with distinct transcriptional signatures

**Fig. 3:**
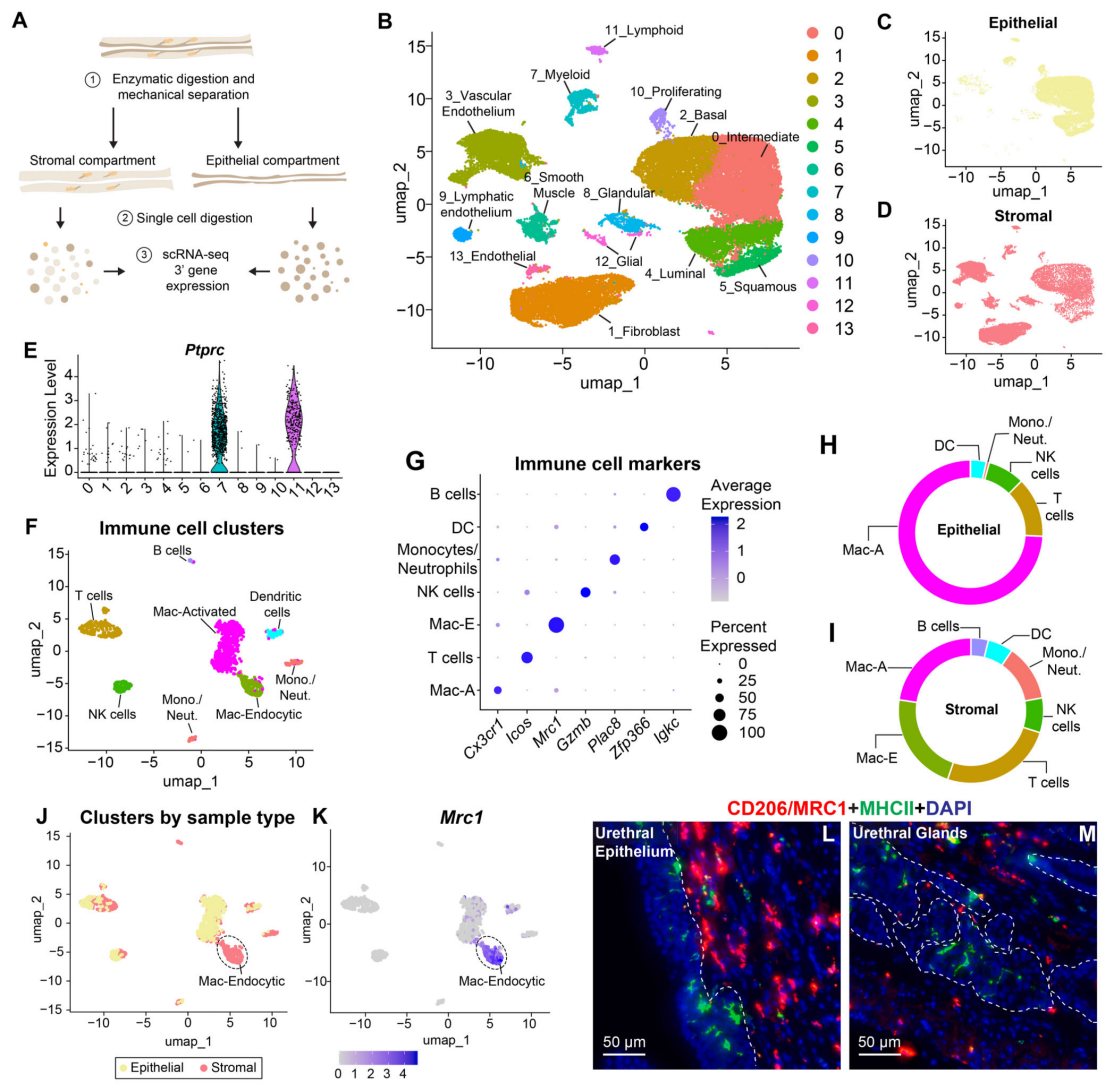
Distribution of macrophage populations in the epithelial and stromal compartments (A) Schematic depicting enzymatic digestion and mechanical separation of the epithelial and stromal compartments of the urethra followed by single cell digestion and RNA-sequencing. (B) UMAP depicting major cell clusters from the female mouse urethral epithelial and stromal compartments. UMAP of all cells split by sample type showing cells from the (C) epithelial and (D) stromal preparations. (E) Violin plot for *Ptprc*/CD45 expression identifying immune cell clusters. (F) UMAP of immune cells. (G) Dot plot showing markers for each immune cell cluster. (H) Donut plot depicting proportions of immune cell clusters in the urethral epithelial compartment. (I) Donut plot depicting proportions of immune cell types in the urethral stromal compartment. (J) UMAP of immune cells showing sample type. (K) Feature plot showing expression of *Cd206/Mrc1* in the Mac-Endocytic macrophage cluster which is predominantly present in the stromal preparation. Section of the adult female mouse urethra depicting (L) epithelium and (M) urethral glands labeled with antibodies to MHCII protein (in green) and CD206 (in red). MHCII high Mac-Activated macrophage cells predominate in the urethral epithelium and glands while CD206 + cells are present in the stromal compartment. Nuclei are labeled in blue. Scale bar represents 50 μm. Images are representative of n = 4 mice/group. Dotted line represents border between epithelium and stroma. Abbreviations: Mono./Neut.: Monocyte/Neutrophil, NK cells: Natural Killer cells, DC: Dendritic cells, Mac-A: Macrophage Activated, Mac-E: Macrophage Endocytic. (For interpretation of the references to colour in this figure legend, the reader is referred to the web version of this article.)

Having observed the infiltration of macrophages into the epithelial layer during development, we hypothesized that either the Mac-A or Mac-E populations would correspond to epithelial-associated macrophages. To test the distribution of Mac-A and Mac-E populations, we performed single cell RNA-sequencing on isolated urethral epithelium and stromal tissue compartments. The epithelial layer of the urethra was separated from the stromal layer by enzymatic digestion followed by mechanical separation and single cell digestion (Fig. 3 A). Single cell RNA-sequencing was performed and major cell clusters were identified (Fig. 3 B, Table S9). The epithelial cell preparation contained minimal cells corresponding to fibroblasts or smooth muscle cells (Fig. 3 C). The stromal cell preparation contained some epithelial cells, presumably contributions from urethral ducts and glands that extend into the stromal compartment from the main trunk of the urethral lining (Fig. 3 D). Two cell clusters corresponding to immune cells (*Ptprc* +) were sub-setted and re-clustered (Fig. 3 E-F, Table S10). We identified Mac-A and Mac-E clusters characterized by *Cx3cr1* and *Mrc1*/*Cd206* expression respectively (Fig. 3 G).

Next, we assessed the distribution of immune cell clusters in the epithelial and stromal preparations (Table S11). Macrophages are the most abundant cell type in each compartment (Fig. 3 H-I). The stromal compartment contains a substantial proportion of T cells and smaller proportions of NK cells, monocytes/neutrophils, dendritic cells and B cells (Fig. 3 I). We also observe T cells in the epithelial compartment (Fig. 3 H). Immunostaining with the T cell marker CD3e shows T cells closely associated with the epithelial and sub-epithelial space in addition to T cells associated with urethral glands (Fig. S4 A-B). Dendritic cells and NK cells are also present in the epithelial compartment (Fig. 3 H).

The epithelial compartment consists of only one macrophage cluster, the Mac-A. The stromal compartment contains both Mac-A and Mac-E cells (Fig. 3 H-I). Assessment of *Mrc1* expression revealed that high *Mrc1* expression is confined to immune cells from the stromal compartment and corresponds to the Mac-E cluster (Fig. 3 J-K). The presence of Mac-A cells in the stromal compartment could be explained by their residence in urethral glands which segregate with the stromal separation (Fig. 3 A). Indeed, F4/80 + macrophages are abundantly present in association with epithelial cells from urethral glands (Fig. S4 C-D). We marked Mac-A cells with antibodies to MHCII and Mac-E cells with MRC1/CD206. The two markers show distinct localization with MHCII high cells localizing to the urethral epithelium and urethral glands while CD206 expressing cells are present only in the stromal compartment (Fig. 3 L-M). In summary, we identified spatially separated macrophage populations with distinct transcriptional identities in the epithelial and stromal compartments of the urethra.

### Epithelial-associated Mac-A macrophages express inflammatory markers and display a dendritic morphology

**Fig. 4:**
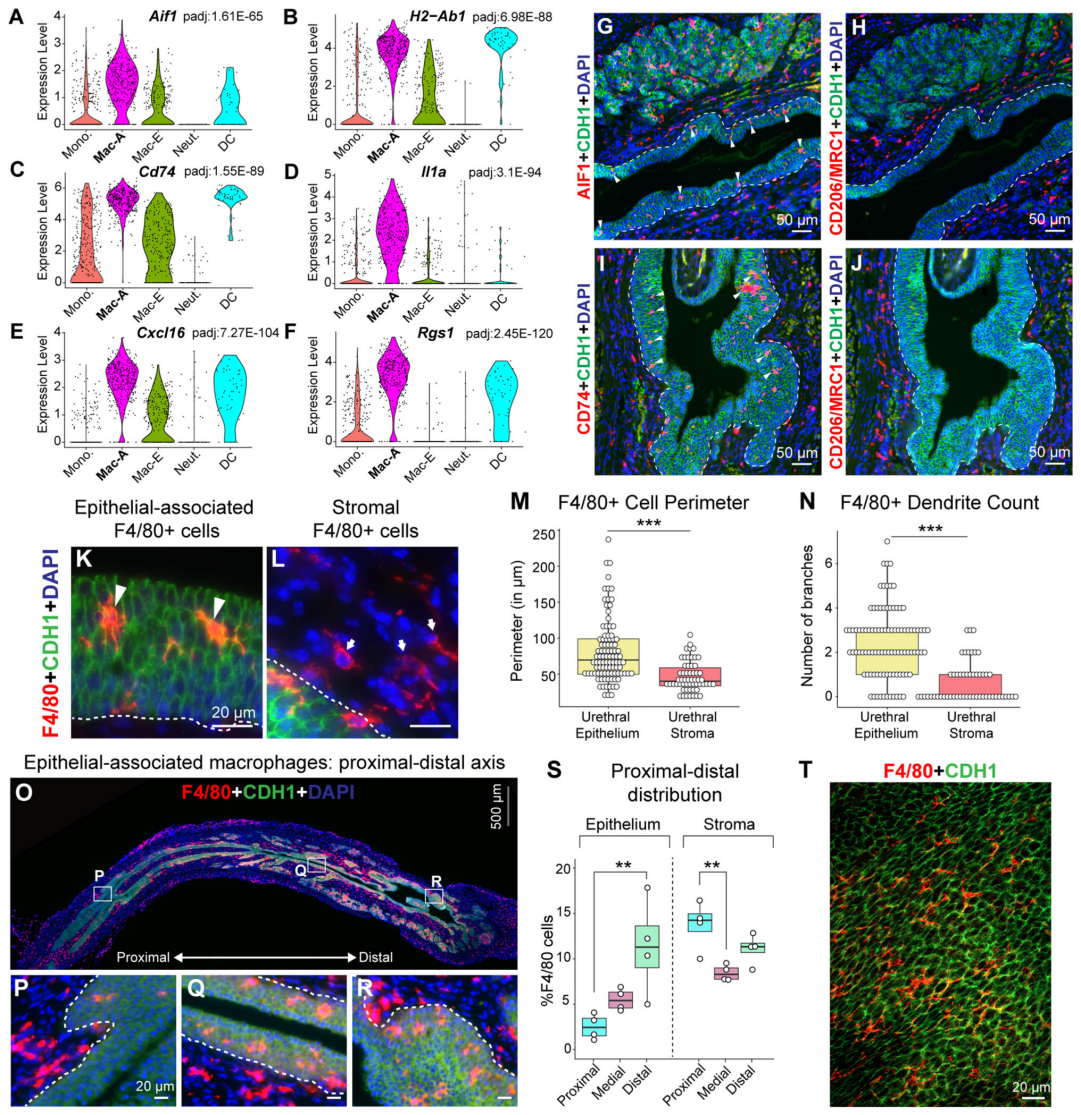
Mac-Activated macrophage population resides in the epithelial compartment of the urethra and display a dendritic morphology. Violin plots showing expression of (A) *Aif1*, (B) *H2-Ab1*, (C) *Cd74*, (D) *Il1a*, (E) *Cxcl16* and (F) *Rgs1*. Genes represent marker genes for the Mac-A cluster which show differential expression in Mac-A cluster compared to other clusters using the Wilcoxon rank sum test performed using the FindAllMarkers function in Seurat. Adjusted p values for differential expression of these genes in the Mac-A cluster are provided with the violin plots. Serial sections of the adult female mouse urethra labeled with antibodies to (G) AIF1 (in red) and CDH1 (in green) and (H) CD206 (in red) and CDH1 (in green). Serial sections of the adult female mouse urethra labeled with antibodies to (I) CD74 (in red) and CDH1 (in green) and (J) CD206 (in red) and CDH1 (in green). Nuclei are labeled in blue. Images are representative of n = 4 mice/group. Scale bar represents 50 μm. White arrowheads indicate epithelial-associated macrophages. Dotted line represents border between epithelium and stroma. (K-L) Tissue sections from the adult female mouse urethra labeled with antibodies against the macrophage marker F4/80 (in red) and the epithelial protein CDH1 (in green). Images are representative of least n = 4 mice/group. White scale bar represents 20 μm. White arrowheads indicate epithelial-associated macrophages. White arrows indicate stromal macrophages. Dotted line represents border between epithelium and stroma. (M) Quantification of cell perimeter of F4/80 + macrophages in the epithelial and stromal compartments. (N) Quantification of dendrite counts of F4/80 + macrophages in the epithelial and stromal compartments. (O) Scan of a 5-μm tissue section of an adult female mouse urethra labeled with antibodies against the macrophage marker F4/80 (in red) and the epithelial protein CDH1 (in green). Magnified images from (O) depicting the (P) proximal, (Q) medial and (R) distal urethra. (S) Quantification of macrophages in the epithelial and stromal compartment along the proximal–distal axis of the urethra. Images are representative of least n = 4 mice/group. White scale bar represents 20 μm. Gray scale bar represents 500 μm. Nuclei are labeled in blue. (T) Wholemount of isolated female mouse urethral epithelial lining labeled with antibodies against F4/80 (in red) and the epithelial protein CDH1 (in green). Images are representative of least n = 3 mice/group. White scale bar represents 20 μm. * p < 0.05, ** p < 0.01, *** p < 0.001 from Wilcoxon Rank sum test (M-N) or Kruskal-Wallis and Dunn’s test (S-Epithelium) or one-way ANOVA and Tukey’s HSD test (S-Stroma). (For interpretation of the references to colour in this figure legend, the reader is referred to the web version of this article.)

We further assessed gene expression, localization and cellular morphology of epithelial-associated Mac-A macrophages. Mac-A macrophages express higher levels of genes involved in inflammatory processes including *Aif1, H2-Ab1, Cd74, Il1a, Cxcl16* and *Rgs1* compared to the Mac-E population (Fig. 4 A-F). Immunostaining of the female urethra with antibodies against AIF-1/IBA-1 (Mac-A marker) and CD206 (Mac-E marker) revealed that AIF-1+ cells localize to the urethral epithelial lining and urethral glands while CD206+ cells are confined to the stromal compartment (Fig. 4 G-H). Similarly, CD74+ Mac-A cells are embedded in the urethral epithelial lining while CD206+ Mac-E cells are observed only in the stromal regions (Fig. 4 I-J). We observed a similar distribution of CD74+ and CD206+ cells in the male urethra. CD74+ cells were present within the epithelial lining and in the sub-epithelial space while CD206+ cells were widely distributed in the stromal compartment (Fig. S5).

We assessed the cellular morphology of epithelial-associated macrophages compared to macrophages present in the stromal compartment of the urethra (Fig. 4 K-L). Epithelial-associated macrophages have a larger cell perimeter than their stromal counterparts (Fig. 4 M). Epithelial-associated macrophages have several dendritic projections which are sent in between the junctions of epithelial cells of the urethra. In contrast, stromal macrophages have fewer dendrites and a spindle-shaped appearance (Fig. 4 N). Given the numerous dendrites and localization to the urethral epithelium, epithelial-associated Mac-A macrophages could be performing immune surveillance of urethral lumen contents.

We investigated the distribution of epithelial-associated macrophages across the proximal–distal axis of the urethra. Higher numbers of epithelial-associated macrophages reside in the distal urethral epithelium compared to the proximal urethral epithelium (Fig. 4 O-S). Wholemount preparations of urethral epithelium labeled with antibodies to F4/80 show macrophages forming an arbor of dendrites in the epithelial lining (Fig. 4 T).

Given the location of Mac-A cells in the epithelial lining, we tested whether these cells are able to detect and respond to inflammatory stimuli from the urethral lumen. We compared the transcriptional profiles of Mac-A cells from urethras of female mice that had PBS or bacterial LPS instilled into the lower urinary tract. At 3 h post-instillation, Mac-A cells from LPS treated mice showed higher expression of the cytokines *Il15, Cxcl10, Cxcl11* and *Ccl5* compared to Mac-A cells from PBS treated mice. Mac-A cells in the LPS instilled mice showed enrichment of innate immune signaling pathways including Toll-like receptor signaling pathway, TNF signaling pathway, RIG-I-like receptor signaling pathway and NOD-like receptor signaling pathway. (Fig. S6, Table S12). This suggests that Mac-A cells can respond to inflammatory stimuli in the urethral lumen by upregulating innate immune signaling pathways.

Mac-A cells are distinct from resident dendritic cells (DCs). DCs are present in smaller proportions than macrophages in both the stromal and epithelial compartments of the urethra (Fig. 3 H-I). We observed that CD11c high DCs only colonize the urethral epithelium around the time of weaning, with the highest percentage of DCs in the urethral epithelium observed at the adult stage (Fig. S7 A-H). Given that epithelial-associated macrophages are present from embryonic stages, it appears that DCs represent a different population than Mac-A. Additionally, co-staining of CD11c and CD86 with F4/80 shows that CD11c and CD86 high cells are distinct from F4/80+ epithelial-associated macrophages (Fig. S7 I-J). Mac-A macrophages and DCs show high expression of several inflammatory markers like *Cd74* and *H2-Ab1* (Table S4). However, DCs exclusively express the receptor *Ccr7*, the dendritic cell specific transcription factors *Zfp366* (DC-SCRIPT)^31^ and *Zbtb46*^32^ in addition to the CSF-2 receptor, *Csf2rb2* distinguishing them from Mac-A macrophages (Fig. S7 K).

Overall, we show that activated Mac-A macrophages with an inflammatory gene signature and multiple dendrites reside in the urethral epithelial compartment.

### Mac-E macrophages reside in the stromal compartment and represent a homeostatic tissue resident macrophage population

**Fig. 5:**
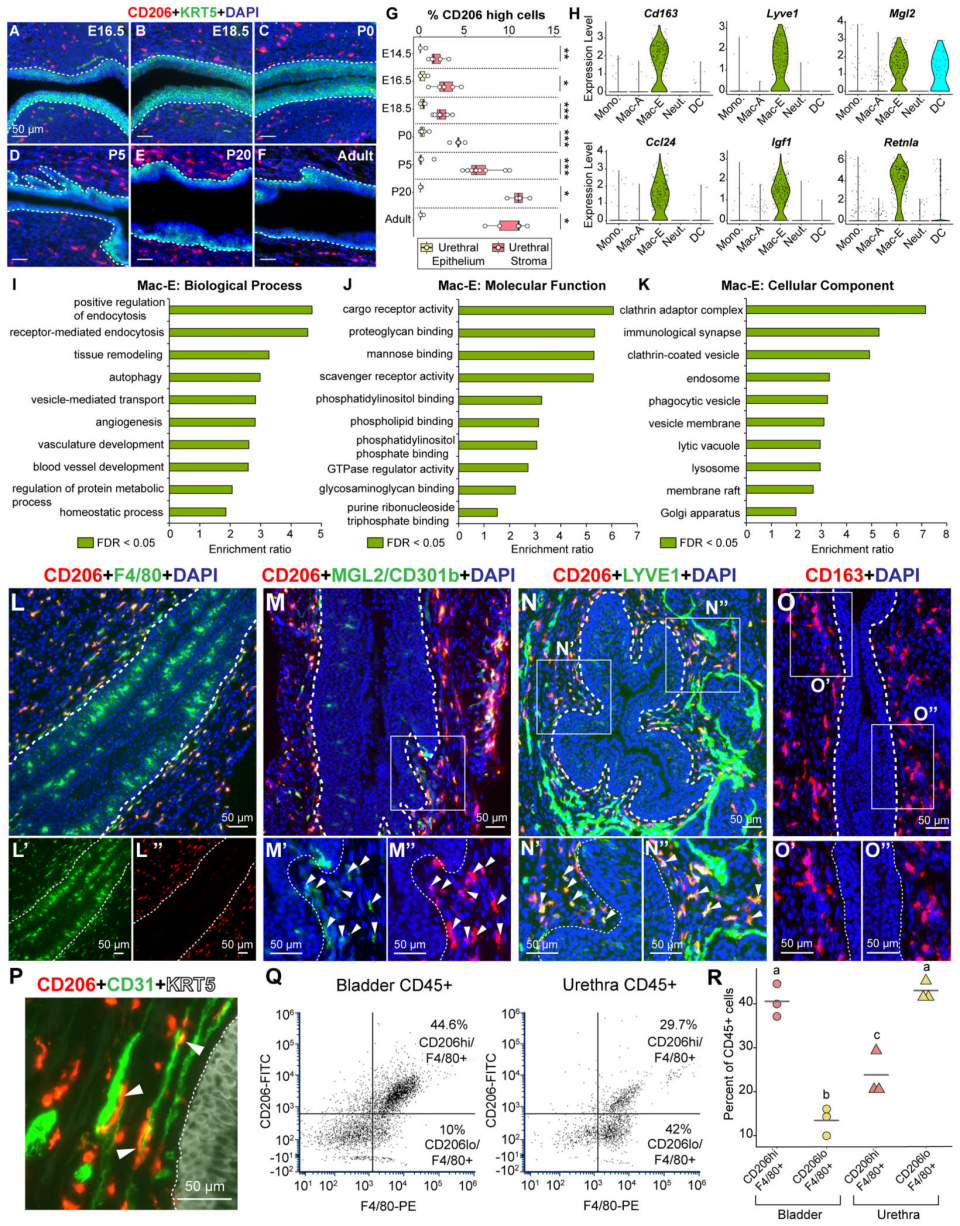
Mac-Endocytic macrophage population resides in the stromal compartment of the urethra. Tissue sections of the urethra from (A) E16.5 mice, (B) E18.5 female mice, (C) P0 female mice, (D) P5 female mice, (E) P20 female mice and (F) adult female mice were labelled with antibodies to CD206/MRC1 (Mac-E marker, in red) and Keratin 5 (basal epithelial marker, in green). (G) Quantification of CD206 high macrophages in the urethral epithelium and urethral stroma across different stages. * p < 0.05, ** p < 0.01, *** p < 0.001 from Wilcoxon Rank sum test or Student’s *t*-test. Images and data are representative of at least n = 4–9 mice/group from at least n = 2 independent litters. (H) Violin plots showing expression of genes *Cd163, Lyve1, Mgl2, Ccl24, Igf1* and *Retnla* which are differentially expressed in the Mac-E macrophage population. Direct differential expression comparison was performed between Mac-E and Mac-A macrophages. Genes with adjusted p-value < 0.0001 and positively upregulated in the Mac-E cluster were chosen for gene ontology analysis. Gene ontology analysis using WebGestalt was performed for (I) Biological process, (J) Molecular function and (K) Cellular component. Selected gene ontology terms with FDR < 0.05 are represented in the plots. (L) Tissue section from an adult female mouse urethra labeled with antibodies to CD206/MRC1 (in red) and F4/80 (in green). L’ and L” show isolated green channel and red channel separately. (M) Tissue section from an adult female mouse urethra labeled with antibodies to CD206 (in red) and MGL2/CD301b (in green). Magnified image from inset shown in M’ and M”. White arrowheads indicate cells co-expressing CD206 and CD301b in the stromal compartment (N) Tissue section from an adult female mouse urethra labeled with antibodies to CD206 (in red) and LYVE1 (in green). Magnified image from insets shown in N’ and N”. White arrowheads indicate cells co-expressing CD206 and LYVE1 in the stromal compartment. (O) Tissue section from an adult female mouse urethra labeled with anti-bodies to CD163 (in red). Magnified image from insets shown in O’ and O”. (P) Sections from adult female mouse urethra labeled with antibodies to CD206 (in red), CD31 (endothelial cell marker, green) and KRT5 (basal epithelial marker, in white). White arrowheads indicate CD206 + macrophages in close proximity to blood vessels. Nuclei are labeled in blue. Scale bar represents 50 μm. White dotted line represents border between epithelium and stroma. All fluorescent images are representative of n = 4 mice. (Q) Flow cytometry was performed on single cell preparations of pooled whole mouse urethras and bladders to assess expression of CD206 and F4/80. Live cells were assessed for CD45 expression. CD45 + cells were assessed for F4/80 and CD206 expression. Plots are representative of n = 3 pooled samples/group (R) Quantification of flow cytometry data from n = 3 pooled samples/group. One-way ANOVA and Tukey’s HSD posthoc test was performed. (For interpretation of the references to colour in this figure legend, the reader is referred to the web version of this article.)

The Mac-E population marked by *Mrc1/Cd206* expression is present exclusively in the stromal compartment (Fig. 3 I). We assessed the emergence of CD206+ cells over the course of development. CD206+ expressing macrophages are present during embryonic development and their numbers increase in the urethral stromal compartment over time. The epithelial compartment of the urethra is mostly devoid of Mac-E CD206-high cells at all stages (Fig. 5 A-G). We further assessed the gene expression signature of this population. The Mac-E population is marked by expression of multiple scavenging receptors including *Mrc1* (Fig. 2 D), *Cd163, Lyve1* and *Mgl2*, the chemokine *Ccl24*, the growth factor *Igf1* and the M2 macrophage marker *Retnla* (Fig. 5 H).

We performed gene ontology analysis with genes differentially expressed in the Mac-E cluster compared to the Mac-A cluster with a stringent cut-off of false discovery rate (FDR) < 0.0001 (Table S13). Biological processes enriched in the Mac-E cluster include receptor-mediated endocytosis, autophagy, angiogenesis, blood vessel morphogenesis, homeostatic process and tissue remodeling (Fig. 5 I, Table S13). Molecular functions including scavenger receptor activity, mannose binding, proteoglycan binding and cargo receptor activity are enriched in Mac-E cells indicating a potential role in scavenging and endocytosis (Fig. 5 J, Table S13). Cellular components for clathrin adaptor complex, endosome, phagocytic vesicle and lysosome are enriched in the Mac-E cluster (Fig. 5 K, Table S13).

Co-staining of CD206 with the pan macrophage marker F4/80 shows that epithelial-associated macrophages express low to undetectable levels of CD206 while CD206 expression overlaps with F4/80 expression only in the stromal compartment (Fig. 5 L). We tested another marker of Mac-E macrophages, CD301b or MGL2. *Mgl2* encodes for a carbohydrate receptor^33^ that is present on macrophages and dendritic cells^34,35^. We observe MGL2 expression only in the urethral stroma where it overlaps with CD206 expression (Fig. 5 M). LYVE1 high cells are present in the stromal compartment where they co-express CD206 (Fig. 5 N). In addition to macrophages, LYVE1 is also expressed on lymphatic endothelium (Fig. 3 B, Table S9). CD163, the scavenging receptor for the hemoglobin-haptoglobin complex^36^, is present only in the stromal compartment of the urethra (Fig. 5 O). Overall, we have demonstrated by multiple markers that the Mac-E population resides in the urethral stroma.

Gene ontology analysis (Fig. 5 I) shows enrichment of biological processes such as angiogenesis and blood vessel morphogenesis in the Mac-E population. Mac-E cells express several genes with known angiogenic functions (Table S4) including growth factors *Igf1* ^37^, *Hbegf* ^38^ and *Tgfb1* ^39^, cytokines *Ccl2* ^40^ and *Ccl24* ^41^ and matrix and matrix-remodeling factors *Emilin2* ^42^, *Ecm1* ^43^ and *Mmp9* ^44^. Immunostaining shows Mac-E macrophages lying in close proximity to endothelial cells in the urethral stroma (Fig. 5 P).

Previous work had described two transcriptionally and spatially distinct macrophage populations in the mouse bladder^20^. Similar to the two urethral macrophage populations, bladder macrophages can be distinguished by expression of *Mrc1* and *Cx3cr1*. One population in the bladder expresses high levels of *Mrc1/Cd206* and LYVE1 (Mac-M) and resides in the bladder muscle while the other expresses high levels of CX3CR1 (Mac-L) and is present in the lamina propria, just below the epithelial lining^20^. However, unlike the urethra there are no epithelial-associated macrophages in the bladder. We compared the proportions of CD206 high (Mac-E) and low (Mac-A) macrophage populations in the adult female bladder and urethra. CD206 high/F4/80+ Mac-E macrophages are more abundant in the bladder compared to the urethra. CD206 low/F4/80+ Mac-A macrophages are more abundant in the urethra compared to the bladder (Fig. 5 Q-R, Fig. S8). To confirm this at the transcriptional level, we integrated a published dataset^28^ of young mouse (3-month-old) bladder immune cells with immune cells from our female mouse urethra dataset. Clustering of myeloid populations reveals shared macrophage subtypes in the bladder and urethra which express markers of Mac-A (*Cx3cr1, Ms4a7, Rgs1, Il1a*) and Mac-E (*Mrc1, Folr2, Ccl24, Pf4*) (Fig. S9, Table S14). Mac-A cells are lower in abundance in the bladder with the majority of bladder macrophages being Mac-E cells (Fig. S9). These results indicate similar macrophage subtypes across the lower urinary tract, with differences in macrophage localization based on the organ site.

In summary, Mac-E macrophages in the urethra reside in the stromal compartment and express gene signatures indicating their participation in endocytosis, tissue remodeling, angiogenesis and homeostatic processes.

### Spatial mapping of epithelial populations in the urethra reveals secretory function of urethral luminal epithelial cells and urethral glands

**Fig. 6:**
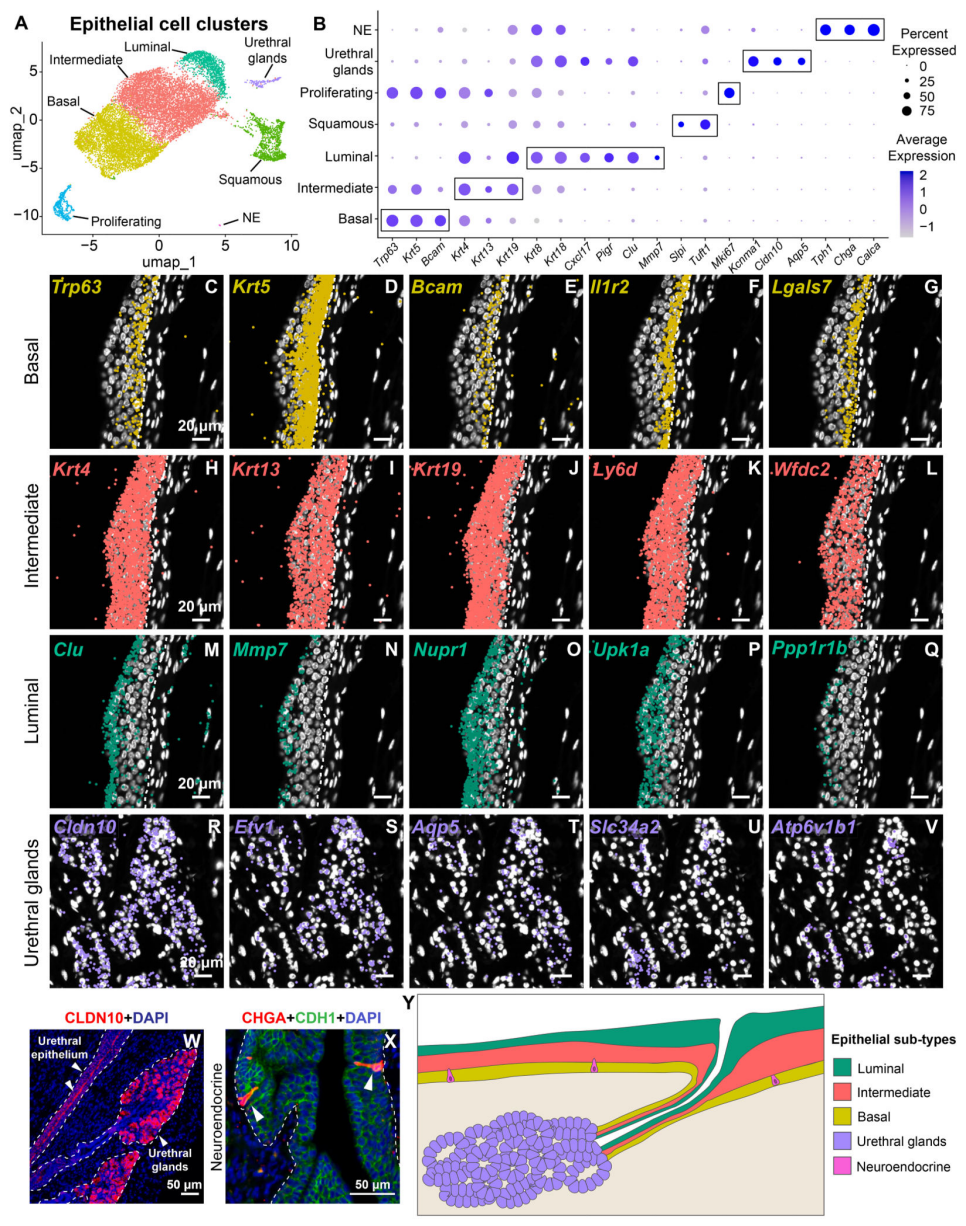
Spatial mapping of epithelial cell subtypes in the urethra. (A) UMAP depicting major epithelial cell clusters from the female mouse urethra. (B) Dot plot of markers expressed by epithelial cell clusters. Xenium expression data from the adult female mouse urethra showing localization of probes against the basal epithelial marker genes (C) *Trp63*, (D) *Krt5*, (E) *Bcam*, (F) *Il1r2* and (G) *Lgals7*. Xenium expression data from the adult female mouse urethra showing localization of probes against the intermediate epithelial marker genes (H) *Krt4*, (I) *Krt13*, (J) *Krt19*, (K) *Ly6d* and (L) *Wfdc2*. Xenium expression data from the adult female mouse urethra showing localization of probes against the luminal epithelial marker genes (M) *Clu*, (N) *Mmp7*, (O) *Nupr1*, (P) *Upk1a* and (Q) *Ppp1r1b*. Xenium expression data from the adult female mouse urethra showing localization of probes against the glandular epithelial marker genes (R) *Cldn10*, (S) *Etv1*, (T) *Aqp5*, (U) *Slc34a2* and (V) *Atp6v1b1*. Scale bar represents 20 μm. Nuclei are labeled in white. (W) Tissue section from adult female mouse urethra labeled with antibodies to CLDN10 (in red) depicting urethral glands connected to urethral epithelium. Images representative of n = 3 mice. Nuclei are labeled in blue. Scale bar represents 50 μm. White dotted line indicates border between epithelium and stroma. (X) Tissue section from the adult female mouse urethra labeled with antibodies to the neuroendocrine cell marker CHGA (in red) and the epithelial cell marker CDH1 (in green). Images representative of n = 4 mice. Nuclei are labeled in blue. Scale bar represents 50 μm. Arrowheads indicate neuroendocrine cells. White dotted line indicates border between epithelium and stroma. (Y) Schematic of epithelial cell layer organization in the adult female mouse urethra. Abbreviations: NE:Neuroendocrine. (For interpretation of the references to colour in this figure legend, the reader is referred to the web version of this article.)

The transcriptional and morphological differences between epithelial and stromal macrophages suggests that residence in the epithelium could influence the functional identity of tissue resident macrophages. We mapped epithelial cell types in the urethra by employing single cell and spatial transcriptomic approaches. Epithelial cell clusters were subsetted and re-clustered to reveal the granularity of epithelial subpopulations in the urethra (Fig. 6 A, Fig. S2 C-D, Table S15). The major populations identified are basal epithelial (*Krt5, Trp63, Bcam*), intermediate epithelial (*Krt4, Krt13, Krt19*) and luminal epithelial (*Krt8, Krt18, Cxcl17, Pigr*). In addition, an epithelial cell type corresponding to the urethral glands (*Cldn10, Aqp5, Kcnma1*) is also present. We also observe a small cluster with gene signatures corresponding to neuroendocrine cells (*Tph1, Chga, Calca*). In addition, a squamous-like population is also present (*Slpi, Tuft1, Plaur*) (Fig. 6 B, Table S15).

Using Xenium spatial transcriptomics, we mapped marker genes for the major epithelial clusters onto a section of the adult female urethra. The basal markers *Trp63, Krt5, Bcam, Il1r2* and *Lgals7* localize to the urethral epithelium towards the basement membrane. Expression of these markers is absent in the apical layers of the urethral epithelium (Fig. 6 C-G, Fig. S10 A-B). The intermediate epithelial markers *Krt4, Krt13, Krt19, Ly6d* and *Wdfc2* have a wider zone of expression (Fig. 6 H-L, Fig. S10 C-D). This is corroborated by the single cell data (Fig. 6 B) which shows a higher expression of these markers in the intermediate layer compared to the basal epithelial layer. Luminal epithelial marker genes *Clu, Mmp7, Nupr1, Upk1a* and *Ppp1r1b* localize to the apical-most layers of the urethra (Fig. 6 M-Q, Fig. S10 A-B). The gene signature of the luminal epithelium includes secreted proteins like *Mmp7, Cxcl17, Lgals3, Spint1, Clu* as well as those involved in membrane function like the chloride channel regulator *Clca1* and the Polymeric immunoglobulin receptor *Pigr* (Table S15).

Urethral glands are connected to the main urethral tube lumen by short ducts. Urethral gland epithelial cells are arranged in small acinilike clusters marked by strong expression of CLDN10 protein (Fig. 6 R, W, Fig. S10 C-D). Urethral glands express the salivary gland transcription factor *Etv1* ^45^ (Fig. 6 S) and the water channel protein *Aqp5* (Fig. 6 T). Urethral glands also express the potassium transporter *Kncma1* and the sodium-dependent phosphate transporter *Slc34a2* (Fig. 6 U). In addition, urethral gland cells express the muscarinic receptor *Chrm3* (Table S15) which suggests that secretion from these glands is mediated by acetylcholine ^46^. The expression of the gene *Atp6v1b1* (Fig. 6 V), encoding a subunit of vacuolar H + -ATPase^47^, indicates that secretions from urethral glands may be involved in acidification of the urethral lumen. Urethral glands also express genes for secreted proteins like *Egf, Cxcl17* and *Clu* (Table S15).

Neuroendocrine cells are rare epithelial cells interspersed in the urethral lining. These cells express the neuroendocrine secretory protein *Chga* (Fig. 6 X) and the neurotransmitter *Calca* (Table S15). Additionally, these cells express the Tyrosine hydroxylase gene *Tph1* which is required for serotonin biosynthesis and defense against pathogens^3^ (Table S15).

Overall, we have generated a comprehensive spatial and single cell resolution map of epithelial cells in the female mouse urethra, identifying novel cell types (Fig. 6 Y).

### Chemokine gradients along the proximal–distal axis of the urethra correspond with macrophage density

**Fig. 7:**
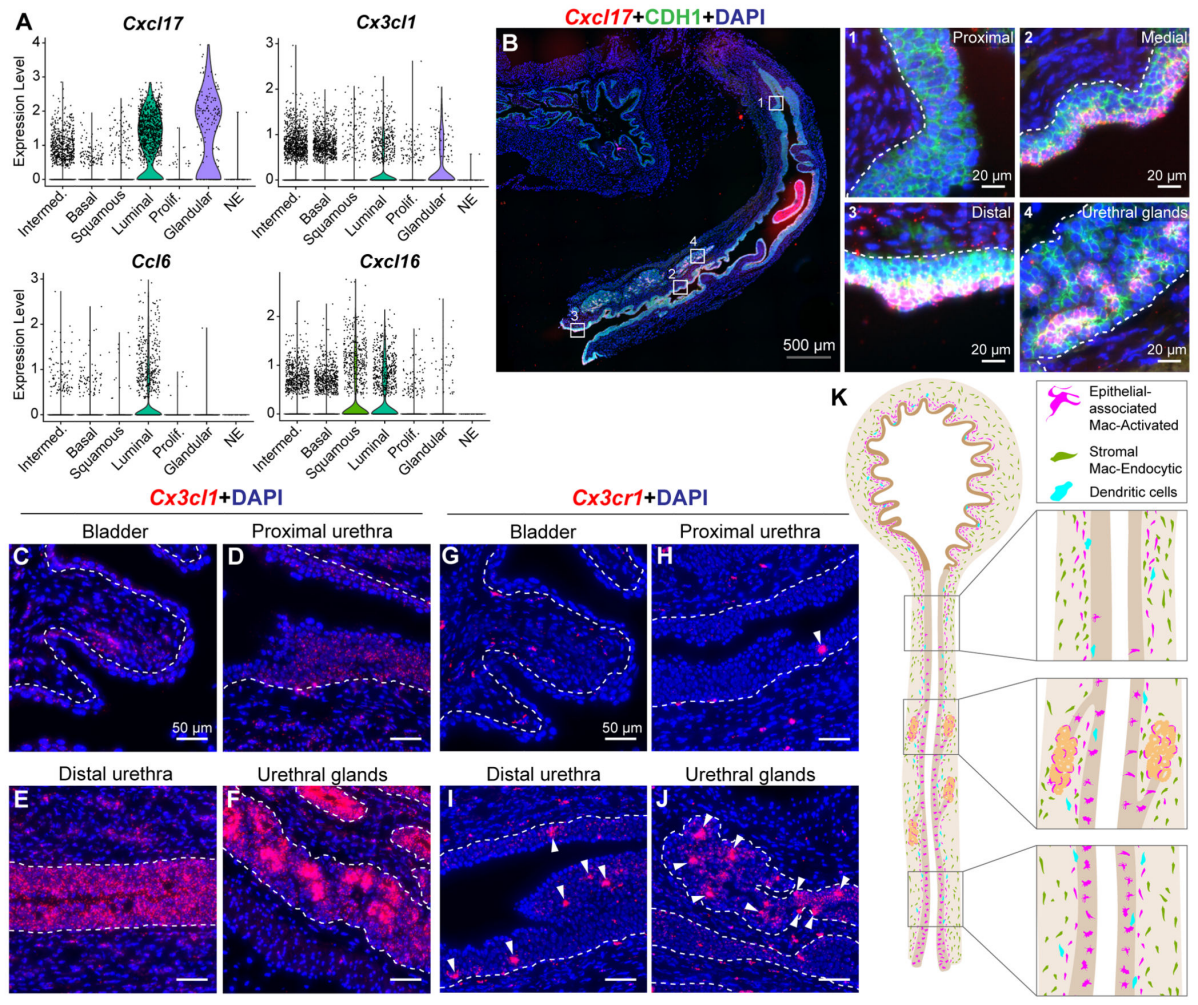
Chemokine expression in the adult female mouse urethral epithelium. Violin plots showing expression of chemokines (A) *Cxcl17, Cx3cl1, Ccl6* and *Cxcl16* in urethral epithelial cells. (B) Tissue section from adult female mouse lower urinary tract labeled with probes against *Cxcl17* mRNA (in red) and the epithelial marker CDH1 (in green). Magnified insets of 1. proximal urethra, 2. medial urethra, 3. distal urethra and 4. glandular regions are shown. Images are representative of n = 4 mice/group. Nuclei are labeled in blue. Gray scale bar represents 500 μm. White scale bar represents 20 μm. White dotted line represents border between epithelium and stroma. Tissue section of the adult female mouse lower urinary tract showing (C) bladder, (D) proximal urethra, (E) distal urethra and (F) urethral glands labeled with probes against *Cx3cl1* mRNA (in red). Images representative of n = 4 mice/group. Nuclei are labeled in blue. White scale bar represents 50 μm. White dotted line represents border between epithelium and stroma. Tissue section of the adult female mouse lower urinary showing (G) bladder, (H) proximal urethra, (I) distal urethra and (J) urethral glands were labeled with probes to receptor *Cx3cr1* (in red). Images representative of n = 4 mice/group. Nuclei are labeled in blue. White scale bar represents 50 μm. White dotted line represents border between epithelium and stroma. (K) Schematic showing distribution of macrophage subtypes and dendritic cells in the adult female mouse urethra. (For interpretation of the references to colour in this figure legend, the reader is referred to the web version of this article.)

Chemokine expression by urethral epithelial cells may influence the spatial distribution of resident immune cells. Constitutive expression of the mucosal chemokine *Cxcl17* is observed in urethral luminal cells and urethral glands. Additionally, *Cx3cl1* expression is observed in the urethral epithelium, with expression across all epithelial clusters except neuroendocrine cells (Fig. 7 A).

We assessed whether expression of these chemokines corresponds with the presence of epithelial-associated macrophages. We observe strong expression of *Cxcl17* mRNA in urethral luminal epithelial cells with transcript density increasing along the proximal–distal axis of the urethra. The expression pattern of the chemoattractant *Cxcl17* corresponds with the localization of Mac-A macrophages in the female urethral lining and urethral glands, with increasing numbers of Mac-A cells found along the proximal–distal axis of the urethra (Fig. 4 S). *Cxcl17* expression is absent in the bladder epithelium which is devoid of epithelial-associated macrophages (Fig. 7 B).

*Cxcl17* is the most recent addition to the chemokine family but its receptor has not been identified definitively^48^. To identify a receptor-ligand pair that corresponds to Mac-A localization to the urethral lining, we investigated *Cx3cl1* and its receptor *Cx3cr1. Cx3cr1* shows high expression in the Mac-A cluster (Fig. 2 F, Table S4) while *Cx3cl1* is expressed across urethral epithelial cell clusters (Fig. 7 A). Similar to *Cxcl17, Cx3cl1* shows a gradient of expression in the lower urinary tract epithelium. *Cx3cl1* gene expression is absent in the bladder epithelium, low in the proximal urethral lining and high in the distal urethral lining and urethral glands (Fig. 7 C-F). Correspondingly, *Cx3cr1* expressing Mac-A cells are absent in the bladder epithelium but their numbers increase in the urethral lining along the proximal–distal axis. *Cx3cr1* expressing Mac-A cells are also present in urethral glands (Fig. 7 G-J), revealing a potential link between macrophage organization and epithelial chemokine gradients. We also observe expression of the chemokines *Cx3cl1* and *Cxcl17* in the male urethral epithelium along with the presence of *Cx3cr1*+ cells within the male urethral lining (Fig. S11).

Altogether, the work has generated a spatial and transcriptomic map of major cell populations in the urethra and identified two spatially separated macrophage populations with distinct functional signatures (Fig. 7 K).

## Discussion

The lower urinary tract is a complex and intricate system for the study of immune homeostasis at mucosal sites. While epithelial architecture and resident immune cells in the bladder have been well characterized^20,49^, the epithelial lining of the urethra and associated immune cells have so far been overlooked in the study of mucosal barriers. This study addresses the gap and provides a single cell and spatial transcriptomic map of the adult female mouse urethra. Our work reveals an unexpected patterning of macrophages in the urethra through the identification of spatially separated macrophage populations with distinct morphological and functional characteristics. Mac-A cells were found to be enriched in the urethral epithelial lining while the Mac-E population is exclusive to the stromal compartment. Macrophages are the earliest immune cell types to populate the lower urinary tract. We observe macrophages infiltrating the urethral epithelium during late stages of embryonic development and in increasing numbers postnatally (Fig. 1). Localization of macrophages to the epithelial lining is specific to the urethra, considering that macrophages do not infiltrate the bladder epithelium, instead residing in the sub-epithelial lamina propria space.

The stromal macrophage cluster in the urethra was named Mac-Endocytic (Mac-Endocytic/Mac-E) given the high expression of scavenging receptors *Mrc1/Cd206, Lyve1, Cd163* and *Mgl2* in this population. Gene ontology analysis shows enrichment for endocytic machinery and endocytic pathways in the Mac-E cluster (Fig. 5 I). Gene expression signature of Mac-E macrophages suggests that these cells could have roles in tissue homeostasis by clearance of cellular debris. The expression of the genes *Mrc1, Ccl24* and *Retnla* (Fig. 5) in Mac-E cells suggests a similarity to an ‘M2′ polarized macrophage state^50,51^. Tissue resident macrophages have been implicated in vasculature development^52^ and angiogenesis^53,54^. We observe Mac-E macrophages in close proximity to endothelial cells (Fig. 5P). Gene ontology analysis reveals that Mac-E cells show an enrichment of genes involved in vasculature development and angiogenesis (Table S13). Based on gene expression signatures, Mac-E cells appear to function as tissue resident “homeostatic” macrophages.

Macrophages enriched in the urethral epithelium express genes reminiscent of an ‘M1′ polarized^55^ or activated macrophage population (Mac-Activated/Mac-A). Pathway analysis shows that this cluster is enriched for genes involved in antigen presentation, T cell activation and cytokine signaling (Table S6). Mac-A macrophages localize to the epithelial lining of the urethra and epithelial cells of urethral glands (Fig. 4, Fig. S4 C-D). Strikingly, Mac-A cells display multiple dendrites, with the dendrites projecting between epithelial cell junctions. In a close parallel, Langerhans cells with numerous dendrites residing in the epidermis layer of the skin perform immune surveillance functions by extending and retracting dendrites in a rhythmic manner^15^. Mac-A cells may perform similar functions in the urethra. Our results show that Mac-A cells upregulate cytokines and innate immune genes in response to bacterial lipopolysaccharide from the urethral lumen (Fig. S6, Table S12). Mac-A cells may act as early detectors of inflammatory signals from ascending pathogens in the urethral lumen and transmit cytokines for recruitment of other immune cells.

We observed an increase in the density of Mac-A macrophages along the proximal–distal axis of the urethra. This suggests that there could be a chemical gradient, potentially from microbial content or chemoattractant expression that is influencing the density of Mac-A macrophages in the urethra. Indeed, we observed that expression of chemokines *Cx3cl1* and *Cxcl17* in the urethral epithelium increases along the proximal–distal axis of the urethra (Fig. 7). We have previously shown that *Cx3cl1* and *Cxcl17* expression is observed in the urethral epithelium but not the bladder epithelium during late stages of embryonic development^23^. Here, we show that compartmentalized expression of these chemokines exclusively in the urethral epithelium persists into adulthood. *Cx3cl1* and *Cxcl17* are also expressed in urethral glands (Fig. 7). Interaction of *Cx3cl1* with its receptor *Cx3cr1* expressed on macrophages is critical to the recruitment and maintenance of gut macrophages^56^. We observe localization of *Cx3cr1* expressing Mac-A cells to the *Cx3cl1* expressing urethral epithelium and urethral glands. *Cxcl17* has been described as a homeostatic mucosal chemokine but there is still uncertainty about the identity of its receptor^48^. *Cxcl17* can act as a chemoattractant for monocyte lineage cells in the lung^57^. *Cxcl17* expression is confined to urethral luminal epithelial cells and urethral glands. The expression of *Cxcl17* increases along the proximal–distal axis of the urethral epithelium and correlates with the density of Mac-A macrophages. The role of these chemokines in influencing spatial organization of macrophages in the urethra merits further investigation.

Epithelial-immune interactions shape mucosal sites and inform innate defense strategies. For pathogens ascending the urinary tract, the urethra is the first mucosal site encountered. Although the bladder and urethra are contiguous, a comparison of these two mucosal sites had not been conducted before. We showed previously that chemokine expression is enriched in the developing urethra lining compared to the bladder lining^23^. In the current study, we identified high expression of the chemokines *Cx3cl1* and *Cxcl17* in the adult mouse urethral lining (Fig. 7). For a more comprehensive analysis, we compared single cell transcriptomes of mouse bladder epithelial cells from a previously published study^27^ with urethral epithelial cells from this study. This confirmed that multiple chemokines (*Cxcl1, Cxcl2, Cxcl5, Cxcl15, Cxcl16, Cxcl17, Cx3cl1*) are expressed at higher levels in urethral luminal epithelial cells compared to bladder epithelial cells (Fig. S12, Table S16).

We also compared resident macrophage populations in the bladder and urethra. A study^20^ using flow cytometry to sort CX3CR1 high and low populations from the mouse bladder showed the presence of two spatially distinct macrophage populations. We tested whether these two bladder macrophage subtypes correspond to Mac-A and Mac-E cells in the urethra. Integrating single cell RNA-sequencing data of mouse bladder immune cells from a previous study^28^ with urethral immune cells from the current study, we identified Mac-A and Mac-E like cells in the combined dataset. The proportion of Mac-A cells (*Cx3cr1*+) was higher in the urethra compared to the bladder. Mac-E cells (*Mrc1*+) were the predominant macrophage in the bladder (Fig. S9). In the bladder, the Mac-A like cell type (MacL) resides in the bladder lamina propria adjacent to the epithelium while the Mac-E like cell type (MacM) resides in the bladder muscle layer^20^. It is only in the urethra that macrophages enter the epithelial lining and display dendritic morphology. Further studies are required to test whether this positioning of macrophages can be attributed to expression of chemokine genes *Cx3cl1* and *Cxcl17* in the urethral epithelium (Fig. 7). Overall, our findings show that the urethra is enriched in immunomodulatory genes compared to the bladder and exhibits different spatial distribution of resident macrophage subtypes.

The presence of two transcriptionally distinct macrophage populations in the urethra might indicate different developmental origins corresponding to either embryonic or bone marrow sources. The bladder possesses functionally distinct macrophage populations transcriptionally similar to Mac-A and Mac-E, but lineage tracing in the bladder revealed contributions from both embryonic and bone marrow sources to the two macrophage populations^20^. Similarly, two spatially distinct macrophage populations distinguished by CD74 and LYVE1 expression in the mouse prostate also had contributions from both embryonic and bone marrow derived sources indicating that the populations are not distinct by origin^21^. Adult urethral macrophage pools may still retain embryonic macrophages with postnatal contributions from bone-marrow derived sources to populate the growing organ. The mechanism by which segregation of macrophages occurs in the urethra remains to be elucidated. Proximity to the urethral epithelium at the onset of chemoattractant expression (*Cx3cl1, Cxcl17*) may drive macrophages into the urethral lining where they undergo further functional differentiation. However, it is unclear why only a subset of macrophages expresses the chemokine receptor *Cx3cr1* and whether this determines whether a cell gets sorted into the urethral epithelial compartment.

Epithelial-associated Mac-A cells in the urethra and bladder lamina propria residing MacL cells appear to have similar transcriptomes (Fig. S9), suggesting that proximity to epithelium may shape macrophage phenotypes. However, residence in the epithelium does not seem to be the driver for producing a Mac-A like state as bladder MacL cells do not reside in the epithelial compartment. It remains to be elucidated whether epithelial and stromal compartments of the urethra can act as distinct signaling niches for macrophage differentiation. The specific signals influencing Mac-A and Mac-E cell differentiation require further investigation.

Our findings also shed new light on the intricate, layered structure of the urethral epithelium. The primary lining of the urethral cavity consists of three distinct layers: basal, intermediate, and luminal. Branching from this main epithelial trunk are small ducts that terminate in clusters of acinar glandular epithelium, structures that, until now, had not been described at the transcriptional level. These urethral glands express a variety of ion and water channels, suggesting specialized roles in fluid transport (Table S15). Notably, urethral glands also express a subunit of V-type ATPase, *Atp6v1b1*, an enzyme that could help acidify the urethral lumen as part of the innate defense system^47^. In a pattern reminiscent of other secretory tissues, such as the salivary glands^58^, Mac-A macro-phages are found in close proximity to urethral glands (Fig. S4 C-D), suggesting a potential role for macrophages in modulating secretory activity of the glands.

In conclusion, our study provides a comprehensive cellular atlas of the adult female mouse urethra revealing spatially distinct macrophage populations and secretory epithelial cell types expressing chemokines. This study reinforces the role of the urethra as an active immune barrier with the identification of novel cell types that may play a role in immune surveillance and innate defense. The presence of two distinct macrophage populations suggests complimentary functions in immune defense and tissue remodeling which could be important in the context of urinary tract infections. While this study focuses on the female mouse urethra, our work also reveals distinct macrophage populations in the male mouse prostatic urethra corresponding transcriptionally and spatially to those identified in the female. Single cell RNA-sequencing provides insights into the functions of these cells but further research is required to fully characterize their functions during homeostatic and inflammatory contexts. In particular, the location of Mac-A cells with dendritic morphology within the urethral lining makes them ideal cells to detect infectious agents ascending up the urethra. Epithelial and stromal compartments in the urethra may serve as specialized niches for macrophages and contribute to their functional specification. The regulation of gene expression in these niches could influence differentiation and spatial organization of resident immune populations and in turn affect susceptibility to infection. Our work on the urethra presents a paradigm for compartmentalized gene expression at mucosal barriers and how this influences localization and functional identities of resident immune cell populations.

## Supplementary Material

Supplementary data to this article can be found online at https://doi.org/10.1016/j.mucimm.2025.09.001.

Supplementary file 1 (R code)

Key resources table

Supplementary figures S1-S12

Supplementary table descriptions

Table S1

Table S2

Table S3

Table S4

Table S5

Table S6

Table S7

Table S8

Table S9

Table S10

Table S11

Table S12

Table S13

Table S14

Table S15

Table S16

## Data Availability

Sequence data have been deposited in NCBI GEO under accession numbers GSE293686, GSE304686 and GSE296982. Information on materials and reagents used in this study are provided in the Key resources table.
