# Supplementary material for: Single cell map of the adult female mouse urethra reveals epithelial and stromal macrophages with distinct functional identities
Source: Mucosal Immunol. Author manuscript; Available in PMC 2025 Sep 26. (PMC7618168; doi:10.1016/j.mucimm.2025.09.001)
Supplement: Supplementary file 1 (R code) [file EMS208696-supplement-_Supplementary_file_1__R_code_.pdf]

```
#R scripts for manuscript "Single cell map of the adult female mouse urethra
# reveals epithelial and stromal macrophages with distinct functional identities"

#######################Female mouse urethra: clustering#############################################
####################################################################################################
# Loading required packages

library(dplyr)
library(Seurat)
library(patchwork)
library(hdf5r)

# Load datasets for UR1 and UR2 into Seurat objects GSE293686

mus.UR1=Read10X_h5("UR_1_filtered_feature_bc_matrix.h5", use.names = TRUE, unique.features=TRUE)
mus.UR1=CreateSeuratObject(counts = mus.UR1, project = "UR1")
mus.UR1

mus.UR2=Read10X_h5("UR_2_filtered_feature_bc_matrix.h5", use.names = TRUE, unique.features=TRUE)
mus.UR2=CreateSeuratObject(counts = mus.UR2, project = "UR2")
mus.UR2

# Combining the two Seurat objects
mus=merge(mus.UR1, y = mus.UR2, add.cell.ids = c("UR1", "UR2"), project = "UR1and2")
mus
head(colnames(mus))
table(mus$orig.ident)

# Get percentage mito genes
mus[["percent.mt"]] <- PercentageFeatureSet(mus, pattern = "^mt-")

# Visualize QC metrics as a violin plot
VlnPlot(mus, features = c("nFeature_RNA", "nCount_RNA", "percent.mt"), ncol = 3)

# Filtering by nFeature and percent.mt
mus <- subset(mus, subset = nFeature_RNA > 200 & nFeature_RNA < 5000 & percent.mt < 15)
mus


# Normalizing
mus <- NormalizeData(mus, normalization.method = "LogNormalize", scale.factor = 10000)

# Identification of highly variable features (feature selection)
mus <- FindVariableFeatures(mus, selection.method = "vst", nfeatures = 2000)

# Identify the 10 most highly variable genes
top10 <- head(VariableFeatures(mus), 10)

# Plot variable features with and without labels
plot1 <- VariableFeaturePlot(mus)
plot2 <- LabelPoints(plot = plot1, points = top10, repel = TRUE)
plot1
plot2


# Scaling the data so that highly-expressed genes do not dominate
all.genes <- rownames(mus)
mus <- ScaleData(mus, features = all.genes)



# Perform linear dimensional reduction
mus <- RunPCA(mus, features = VariableFeatures(object = mus))

# Examine PCA results
print(mus[["pca"]], dims = 1:5, nfeatures = 5)

VizDimLoadings(mus, dims = 1:2, reduction = "pca")

DimPlot(mus, reduction = "pca")

DimHeatmap(mus, dims = 1, cells = 500, balanced = TRUE)

DimHeatmap(mus, dims = 1:15, cells = 500, balanced = TRUE)

# Determine the dimensionality of the dataset
ElbowPlot(mus) #For choosing number of PCs

# Clustering
mus <- FindNeighbors(mus, dims = 1:20)
mus <- FindClusters(mus, resolution = 0.2)

head(Idents(mus), 5)

mus <- RunUMAP(mus, dims = 1:20)
```

```
DimPlot(mus, reduction = "umap")


# DEGs

mus[["RNA"]] <- JoinLayers(mus[["RNA"]])
mus.markers <- FindAllMarkers(mus, only.pos = TRUE, min.pct = 0.25, logfc.threshold = 0.25)
write.csv(mus.markers, "MarkerGenes_AllCellsRes0.2.csv")



# Assessing stressed cell clusters

genes=c("Egr1", "Fos", "Jun", "Atf3","Hbegf")

DotPlot(object=mus,assay="RNA",features=genes)

genes.dissoc <- list(c("Atf3", "Btg2", "Cebpb", "Cebpd", "Cxcl3", "Cxcl2", "Cxcl1", "Dnaja1", "Dnajb1", "Dusp1", "Egr1",
"Fos", "Fosb", "Hsp90aa1", "Hsp90ab1", "Hspa1a", "Hspa1b", "Hspa1a", "Hspa1b", "Hspa8", "Hspb1", "Hspe1", "Hsph1", "Id3",
"Ier2", "Jun", "Junb", "Jund", "Nfkbia", "Nr4a1", "Ppp1r15a", "Socs3", "Zfp36"))

mus <- AddModuleScore(mus, features = genes.dissoc, ctrl= 20, name = 'stressed')

FeaturePlot(mus, features = 'stressed1')



# Remove stressed cells

mus <- subset(mus,idents=c("0","2","3","4","6","7","8","9","10","11"))
mus
#Perform linear dimensional reduction
mus <- RunPCA(mus, features = VariableFeatures(object = mus))

# Examine PCA results
print(mus[["pca"]], dims = 1:5, nfeatures = 5)

VizDimLoadings(mus, dims = 1:2, reduction = "pca")

DimPlot(mus, reduction = "pca")

DimHeatmap(mus, dims = 1, cells = 500, balanced = TRUE)

DimHeatmap(mus, dims = 1:15, cells = 500, balanced = TRUE)

#Determine the dimensionality of the dataset
ElbowPlot(mus) #For choosing number of PCs

#Clustering
mus <- FindNeighbors(mus, dims = 1:20)
mus <- FindClusters(mus, resolution = 0.1)

head(Idents(mus), 5)

mus <- RunUMAP(mus, dims = 1:20)

DimPlot(mus, reduction = "umap")


#Identify epithelial clusters

VlnPlot(mus, features = c("Epcam"))

#Identify immune clusters

VlnPlot(mus, features = c("Ptprc"))


mus.markers <- FindAllMarkers(mus, only.pos = TRUE, min.pct = 0.25, logfc.threshold = 0.25)
write.csv(mus.markers, "MarkerGenes_StressfilteredRes0.1.csv")



# Subset immune cells
musImmune <- subset(mus,idents=c("5","8","9"))
musImmune

#Perform linear dimensional reduction
musImmune <- RunPCA(musImmune, features = VariableFeatures(object = musImmune))

# Examine and visualize PCA results
print(musImmune[["pca"]], dims = 1:5, nfeatures = 5)

VizDimLoadings(musImmune, dims = 1:2, reduction = "pca")

DimPlot(musImmune, reduction = "pca")
```

```
DimHeatmap(musImmune, dims = 1, cells = 500, balanced = TRUE)

DimHeatmap(musImmune, dims = 1:15, cells = 500, balanced = TRUE)

#Determine the dimensionality of the dataset
ElbowPlot(musImmune) #For choosing number of PCs

#Clustering
musImmune <- FindNeighbors(musImmune, dims = 1:20)
musImmune <- FindClusters(musImmune, resolution = 0.3)

head(Idents(musImmune), 5)

musImmune <- RunUMAP(musImmune, dims = 1:20)

DimPlot(musImmune, reduction = "umap")

#Dotplot
genes=c("Plac8","Axl","Mrc1", "Gzma", "Trgc1", "Lef1", "S100a9","Igkc","Itga1","Cd209a")
DotPlot(object=musImmune,assay="RNA",features=genes)

genes=c("Fcgr3","Cd3e","Cd4", "Il23r")
DotPlot(object=musImmune,assay="RNA",features=genes)

#Find markers
musImmune.markers <- FindAllMarkers(musImmune, assay="RNA", only.pos = TRUE, min.pct = 0.25, logfc.threshold = 0.25)
write.csv(musImmune.markers, "MarkerGenes_ImmuneRes0.3.csv")

#Save Seurat object
saveRDS(musImmune, file = "musImmune.rds")

musImmune=readRDS(file = "musImmune.rds")



# Subset myeloid cells

musMyeloid <- subset(musImmune,idents=c("0","1","2","6"))

#Perform linear dimensional reduction
musMyeloid <- RunPCA(musMyeloid, features = VariableFeatures(object = musMyeloid))

# Examine and visualize PCA results a few different ways
print(musMyeloid[["pca"]], dims = 1:5, nfeatures = 5)

VizDimLoadings(musMyeloid, dims = 1:2, reduction = "pca")

DimPlot(musMyeloid, reduction = "pca")

DimHeatmap(musMyeloid, dims = 1, cells = 500, balanced = TRUE)

DimHeatmap(musMyeloid, dims = 1:15, cells = 500, balanced = TRUE)

#Determine the dimensionality of the dataset
ElbowPlot(musMyeloid) #For choosing number of PCs

#Clustering
musMyeloid <- FindNeighbors(musMyeloid, dims = 1:20)
musMyeloid <- FindClusters(musMyeloid, resolution = 0.2)

head(Idents(musMyeloid), 5)

musMyeloid <- RunUMAP(musMyeloid, dims = 1:20)

DimPlot(musMyeloid, reduction = "umap")

#Violin plots
VlnPlot(musMyeloid, features = c("Csf1r"))

#Dotplot
genes=c("Ptprc","Adgre1", "Cd68", "Fcgr1", "Csf1r","Cx3cr1","Folr2","Csf2rb")
DotPlot(object=musMyeloid,assay="RNA",features=genes)

#Find markers
musMyeloid.markers <- FindAllMarkers(musMyeloid, assay="RNA", only.pos = TRUE, min.pct = 0.25, logfc.threshold = 0.25)
write.csv(musMyeloid.markers, "MarkerGenes_MyeloidRes0.2.csv")

cluster0.markers <- FindMarkers(musMyeloid, ident.1 = 1, ident.2 = 2, min.pct = 0.25)
head(cluster0.markers, n = 5)
write.csv(cluster0.markers, "Mac1vsMac2Markers.csv")



# Subset epithelial cells
```

```
musEpi <- subset(mus,idents=c("0","1","2","3","7","10"))

#Perform linear dimensional reduction
musEpi <- RunPCA(musEpi, features = VariableFeatures(object = musEpi))

# Examine and visualize PCA results a few different ways
print(musEpi[["pca"]], dims = 1:5, nfeatures = 5)

VizDimLoadings(musEpi, dims = 1:2, reduction = "pca")

DimPlot(musEpi, reduction = "pca")

DimHeatmap(musEpi, dims = 1, cells = 500, balanced = TRUE)

DimHeatmap(musEpi, dims = 1:15, cells = 500, balanced = TRUE)

#Determine the dimensionality of the dataset
ElbowPlot(musEpi) #For choosing number of PCs

#Clustering
musEpi <- FindNeighbors(musEpi, dims = 1:20)
musEpi <- FindClusters(musEpi, resolution = 0.2)

head(Idents(musEpi), 5)

musEpi <- RunUMAP(musEpi, dims = 1:20)

DimPlot(musEpi, reduction = "umap")

#Dotplot
genes=c("Trp63", "Krt5", "Bcam", "Krt4", "Krt13","Krt19","Krt8","Krt18",
"Cxcl17","Pigr","Clu","Mmp7","Slpi","Areg","Tuft1","Mki67","Top2a","Cenpf","Kcnma1","Cldn10","Aqp5","Tph1","Chga","Calca")
DotPlot(object=musEpi,assay="RNA",features=genes)

#Find markers
musEpi.markers <- FindAllMarkers(musEpi, assay="RNA", only.pos = TRUE, min.pct = 0.25, logfc.threshold = 0.25)
write.csv(musEpi.markers, "MarkerGenes_EpiRes0.2.csv")



#############################Female Urethra Epi, Stromal separation###################################
####################################################################################################

# Load datasets for each sample into Seurat objects GSE293686

mus.UrEpi1=Read10X_h5("UREpi_1_filtered_feature_bc_matrix.h5", use.names = TRUE, unique.features=TRUE)
mus.UrEpi1=CreateSeuratObject(counts = mus.UrEpi1, project = "UrEpi1")
mus.UrEpi1

mus.UrEpi2=Read10X_h5("UREpi_2_filtered_feature_bc_matrix.h5", use.names = TRUE, unique.features=TRUE)
mus.UrEpi2=CreateSeuratObject(counts = mus.UrEpi2, project = "UrEpi2")
mus.UrEpi2

mus.UrMes1=Read10X_h5("URSt_1_filtered_feature_bc_matrix.h5", use.names = TRUE, unique.features=TRUE)
mus.UrMes1=CreateSeuratObject(counts = mus.UrMes1, project = "UrMes1")
mus.UrMes1

mus.UrMes2=Read10X_h5("URSt_2_filtered_feature_bc_matrix.h5", use.names = TRUE, unique.features=TRUE)
mus.UrMes2=CreateSeuratObject(counts = mus.UrMes2, project = "UrMes2")
mus.UrMes2

# Merging samples

mus=merge(mus.UrEpi1, y = c(mus.UrEpi2,mus.UrMes1,mus.UrMes2), add.cell.ids = c("UrEpi1", "UrEpi2","UrMes1","UrMes2"),
project = "UrEpiMes")
mus
head(colnames(mus))
table(mus$orig.ident)


# Get percentage mito genes
mus[["percent.mt"]] <- PercentageFeatureSet(mus, pattern = "^mt-")

# Visualize QC metrics as a violin plot
VlnPlot(mus, features = c("nFeature_RNA", "nCount_RNA", "percent.mt"), ncol = 3)

# Filtering by nFeature and percent.mt
mussubset <- subset(mus, subset = nFeature_RNA > 200 & nFeature_RNA < 5000 & percent.mt < 15)
mussubset
mus=mussubset


# Normalizing
mus <- NormalizeData(mus, normalization.method = "LogNormalize", scale.factor = 10000)

# Identification of highly variable features (feature selection)
```

```
mus <- FindVariableFeatures(mus, selection.method = "vst", nfeatures = 2000)

# Identify the 10 most highly variable genes
top10 <- head(VariableFeatures(mus), 10)

# Plot variable features with and without labels
plot1 <- VariableFeaturePlot(mus)
plot2 <- LabelPoints(plot = plot1, points = top10, repel = TRUE)
plot1
plot2


# Scaling the data so that highly-expressed genes do not dominate
all.genes <- rownames(mus)
mus <- ScaleData(mus, features = all.genes)



# Perform linear dimensional reduction
mus <- RunPCA(mus, features = VariableFeatures(object = mus))

# Examine PCA results
print(mus[["pca"]], dims = 1:5, nfeatures = 5)

VizDimLoadings(mus, dims = 1:2, reduction = "pca")

DimPlot(mus, reduction = "pca")

DimHeatmap(mus, dims = 1, cells = 500, balanced = TRUE)

DimHeatmap(mus, dims = 1:15, cells = 500, balanced = TRUE)

# Determine the dimensionality of the dataset
ElbowPlot(mus) #For choosing number of PCs

# Clustering
mus <- FindNeighbors(mus, dims = 1:20)
mus <- FindClusters(mus, resolution = 0.2)

head(Idents(mus), 5)

mus <- RunUMAP(mus, dims = 1:20)

DimPlot(mus, reduction = "umap")

table(mus@meta.data$RNA_snn_res.0.2)

table(mus@meta.data$RNA_snn_res.0.2, mus@meta.data$orig.ident)

VlnPlot(mus, features = c("Krt1"))

FeaturePlot(mus, features = c("Ptprc"))

DimPlot(mus, reduction = "umap", group.by="orig.ident")

# DEGs

mus[["RNA"]] <- JoinLayers(mus[["RNA"]])
mus.markers <- FindAllMarkers(mus, only.pos = TRUE, min.pct = 0.25, logfc.threshold = 0.25)
write.csv(mus.markers, "UrEpiMesMarkerGenes_AllCellsRes0.2.csv")



# Subset immune cells
musImmune <- subset(mus,idents=c("7","11"))
musImmune

#Perform linear dimensional reduction
musImmune <- RunPCA(musImmune, features = VariableFeatures(object = musImmune))

# Examine and visualize PCA results
print(musImmune[["pca"]], dims = 1:5, nfeatures = 5)

VizDimLoadings(musImmune, dims = 1:2, reduction = "pca")

DimPlot(musImmune, reduction = "pca")

DimHeatmap(musImmune, dims = 1, cells = 500, balanced = TRUE)

DimHeatmap(musImmune, dims = 1:15, cells = 500, balanced = TRUE)

#Determine the dimensionality of the dataset
ElbowPlot(musImmune) #For choosing number of PCs

#Clustering
musImmune <- FindNeighbors(musImmune, dims = 1:15)
```

```
musImmune <- FindClusters(musImmune, resolution = 0.2)

head(Idents(musImmune), 5)

musImmune <- RunUMAP(musImmune, dims = 1:15)

DimPlot(musImmune, reduction = "umap")

DimPlot(musImmune, reduction = "umap", group.by="orig.ident")

table(musImmune@meta.data$RNA_snn_res.0.2)

table(musImmune@meta.data$RNA_snn_res.0.2, musImmune@meta.data$orig.ident)

#Feature plots
FeaturePlot(musImmune, features = c("Mrc1"))

#Dotplot
genes=c("H2-Ab1","Icos","Mrc1", "Gzmb", "Plac8", "Itgax", "Igkc")
DotPlot(object=musImmune,assay="RNA",features=genes)

genes=c("Fcgr3","Cd3e","Cd4", "Il23r")
DotPlot(object=musImmune,assay="RNA",features=genes)

#Find markers
musImmune[["RNA"]] <- JoinLayers(musImmune[["RNA"]])
musImmune.markers <- FindAllMarkers(musImmune, assay="RNA", only.pos = TRUE, min.pct = 0.25, logfc.threshold = 0.25)
write.csv(musImmune.markers, "Markers_ImmuneRes0.2.csv")



#######################Male mouse urethra: clustering################################################
#####################################################################################################
# Load the dataset GSE145865

mus.data=Read10X_h5("GSM4338169_musAd004n5_UrF_GEX_filtered_feature_bc_matrix.h5", use.names = TRUE,
unique.features=TRUE)

# Initialize the Seurat object with the raw (non-normalized data).
mus <- CreateSeuratObject(counts = mus.data, project = "mus4n5", min.cells = 3, min.features = 200)
mus

# The [[ operator can add columns to object metadata. This is a great place to stash QC stats
mus[["percent.mt"]] <- PercentageFeatureSet(mus, pattern = "^mt-")

# Visualize QC metrics as a violin plot
VlnPlot(mus, features = c("nFeature_RNA", "nCount_RNA", "percent.mt"), ncol = 3)

# FeatureScatter is typically used to visualize feature-feature relationships, but can be used
# for anything calculated by the object, i.e. columns in object metadata, PC scores etc.

plot1 <- FeatureScatter(mus, feature1 = "nCount_RNA", feature2 = "percent.mt")
plot2 <- FeatureScatter(mus, feature1 = "nCount_RNA", feature2 = "nFeature_RNA")
plot1 + plot2

#Filtering
mus <- subset(mus, subset = nFeature_RNA > 200 & nFeature_RNA < 4000 & percent.mt < 20)

#Normalizing
mus <- NormalizeData(mus, normalization.method = "LogNormalize", scale.factor = 10000)

#Identification of highly variable features (feature selection)
mus <- FindVariableFeatures(mus, selection.method = "vst", nfeatures = 2000)


saveRDS(mus, file = "mus4n5.rds")



# Load the dataset GSE145865

mus.data=Read10X_h5("GSM4338168_musAd002_UrF_filtered_feature_bc_matrix.h5", use.names = TRUE, unique.features=TRUE)

# Initialize the Seurat object with the raw (non-normalized data).
mus <- CreateSeuratObject(counts = mus.data, project = "mus2", min.cells = 3, min.features = 200)
mus

# The [[ operator can add columns to object metadata. This is a great place to stash QC stats
mus[["percent.mt"]] <- PercentageFeatureSet(mus, pattern = "^mt-")

# Visualize QC metrics as a violin plot
VlnPlot(mus, features = c("nFeature_RNA", "nCount_RNA", "percent.mt"), ncol = 3)

# FeatureScatter is typically used to visualize feature-feature relationships, but can be used
# for anything calculated by the object, i.e. columns in object metadata, PC scores etc.
```

```
plot1 <- FeatureScatter(mus, feature1 = "nCount_RNA", feature2 = "percent.mt")
plot2 <- FeatureScatter(mus, feature1 = "nCount_RNA", feature2 = "nFeature_RNA")
plot1 + plot2

#Filtering
mus <- subset(mus, subset = nFeature_RNA > 200 & nFeature_RNA < 4000 & percent.mt < 20)

#Normalizing
mus <- NormalizeData(mus, normalization.method = "LogNormalize", scale.factor = 10000)

#Identification of highly variable features (feature selection)
mus <- FindVariableFeatures(mus, selection.method = "vst", nfeatures = 2000)

saveRDS(mus, file = "mus2.rds")


mus2=readRDS(file = "mus2.rds")
mus4n5=readRDS(file = "mus4n5.rds")

mus_list=list(mus2, mus4n5)

# normalize and identify variable features for each dataset independently
mus_list <- lapply(X = mus_list, FUN = function(x) {
  x <- NormalizeData(x)
  x <- FindVariableFeatures(x, selection.method = "vst", nfeatures = 2000)
})

# Select features that are repeatedly variable across datasets for integration
features <- SelectIntegrationFeatures(object.list = mus_list)

ur.anchors <- FindIntegrationAnchors(object.list = mus_list, anchor.features = features)

#Creating the integrated data assay
mus.combined <- IntegrateData(anchorset = ur.anchors)

#Integrated analysis on combined cells

# specify that we will perform downstream analysis on the corrected data note that the
# original unmodified data still resides in the 'RNA' assay
DefaultAssay(mus.combined) <- "integrated"

# Run the standard workflow for visualization and clustering
mus.combined <- ScaleData(mus.combined, verbose = FALSE)
mus.combined <- RunPCA(mus.combined, npcs = 30, verbose = FALSE)
mus.combined <- RunUMAP(mus.combined, reduction = "pca", dims = 1:30)
mus.combined <- FindNeighbors(mus.combined, reduction = "pca", dims = 1:30)
mus.combined <- FindClusters(mus.combined, resolution = 0.5)

DimPlot(mus.combined, reduction = "umap")

DimPlot(mus.combined, reduction = "umap", group.by = "orig.ident")

DefaultAssay(mus) <- "integrated"

#Clustering
mus <- FindNeighbors(mus, dims = 1:30)
mus <- FindClusters(mus, resolution = 0.1)

head(Idents(mus), 5)

mus <- RunUMAP(mus, dims = 1:30)

DimPlot(mus, reduction = "umap")

#Subset immune cells

VlnPlot(mus, features = c("Ptprc"))

musImmune <- subset(mus,idents=c("8","9"))

#Perform linear dimensional reduction
musImmune <- RunPCA(musImmune, features = VariableFeatures(object = musImmune))

# Examine and visualize PCA results a few different ways
print(musImmune[["pca"]], dims = 1:5, nfeatures = 5)

VizDimLoadings(musImmune, dims = 1:2, reduction = "pca")

DimPlot(musImmune, reduction = "pca")

DimHeatmap(musImmune, dims = 1, cells = 500, balanced = TRUE)

DimHeatmap(musImmune, dims = 1:15, cells = 500, balanced = TRUE)

#Determine the dimensionality of the dataset
ElbowPlot(musImmune) #For choosing number of PCs
```

```
#Clustering
musImmune <- FindNeighbors(musImmune, dims = 1:15)
musImmune <- FindClusters(musImmune, resolution = 0.4)

head(Idents(musImmune), 5)

musImmune <- RunUMAP(musImmune, dims = 1:15)


##VISUALIZATION
DimPlot(musImmune, reduction = "umap")

##FEATURE PLOTS
FeaturePlot(musImmune, features = c("Mrc1"))

##VIOLIN PLOT
VlnPlot(musImmune, features = c("Adgre1"))


##DOTPLOT
genes=c("Nkg7", "Cx3cr1", "Plac8", "Cd209a","Mrc1","Fxyd3","Iglc2","S100a9","Saa3")
DotPlot(object=musImmune,assay="RNA",features=genes)


#FIND MARKERS
musImmune[["RNA"]] <- JoinLayers(musImmune[["RNA"]])
musImmune.markers <- FindAllMarkers(musImmune, assay="RNA", only.pos = TRUE, min.pct = 0.25, logfc.threshold = 0.25)
write.csv(musImmune.markers, "ImmuneMarkersRes0.4.csv")

##########################Bladder and urethra immune integration and clustering######################
#####################################################################################################


#Bladder immune dataset from GSE149571 (GSM4504969      young_1). Unzip files and transfer
#barcodes, genes and and matrix files to a directory

data_dir <- 'C:/Users/HP/Desktop/MouseBladderImmune_Seurat' #change to directory path
list.files(data_dir) # Should show barcodes.tsv, genes.tsv, and matrix.mtx
expression_matrix <- Read10X(data.dir = data_dir)
musBl = CreateSeuratObject(counts = expression_matrix)

# The [[ operator can add columns to object metadata. This is a great place to stash QC stats
musBl[["percent.mt"]] <- PercentageFeatureSet(musBl, pattern = "^mt-")

# Visualize QC metrics as a violin plot
VlnPlot(musBl, features = c("nFeature_RNA", "nCount_RNA", "percent.mt"), ncol = 3)

# FeatureScatter is typically used to visualize feature-feature relationships, but can be used
# for anything calculated by the object, i.e. columns in object metadata, PC scores etc.

plot1 <- FeatureScatter(musBl, feature1 = "nCount_RNA", feature2 = "percent.mt")
plot2 <- FeatureScatter(musBl, feature1 = "nCount_RNA", feature2 = "nFeature_RNA")
plot1 + plot2

#Filtering
musBl <- subset(musBl, subset = nFeature_RNA > 200 & nFeature_RNA < 5000 & percent.mt < 15)

#Normalizing
musBl <- NormalizeData(musBl, normalization.method = "LogNormalize", scale.factor = 10000)

#Identification of highly variable features (feature selection)
musBl <- FindVariableFeatures(musBl, selection.method = "vst", nfeatures = 2000)


saveRDS(musBl, file = "musBlImmune.rds")





# Urethra immune dataset from GSE149571 (GSM4504969     young_1)

mus.UR1=Read10X_h5("UR_1_filtered_feature_bc_matrix.h5", use.names = TRUE, unique.features=TRUE)
mus.UR1=CreateSeuratObject(counts = mus.UR1, project = "UR1")
mus.UR1

mus.UR2=Read10X_h5("UR_2_filtered_feature_bc_matrix.h5", use.names = TRUE, unique.features=TRUE)
mus.UR2=CreateSeuratObject(counts = mus.UR2, project = "UR2")
mus.UR2

# Combining the two Seurat objects
mus=merge(mus.UR1, y = mus.UR2, add.cell.ids = c("UR1", "UR2"), project = "UR1and2")
mus
head(colnames(mus))
table(mus$orig.ident)
```

```
# Get percentage mito genes
mus[["percent.mt"]] <- PercentageFeatureSet(mus, pattern = "^mt-")

# Visualize QC metrics as a violin plot
VlnPlot(mus, features = c("nFeature_RNA", "nCount_RNA", "percent.mt"), ncol = 3)

# Filtering by nFeature and percent.mt
mus <- subset(mus, subset = nFeature_RNA > 200 & nFeature_RNA < 5000 & percent.mt < 15)
mus


# Normalizing
mus <- NormalizeData(mus, normalization.method = "LogNormalize", scale.factor = 10000)

# Identification of highly variable features (feature selection)
mus <- FindVariableFeatures(mus, selection.method = "vst", nfeatures = 2000)

# Identify the 10 most highly variable genes
top10 <- head(VariableFeatures(mus), 10)

# Plot variable features with and without labels
plot1 <- VariableFeaturePlot(mus)
plot2 <- LabelPoints(plot = plot1, points = top10, repel = TRUE)
plot1
plot2


# Scaling the data so that highly-expressed genes do not dominate
all.genes <- rownames(mus)
mus <- ScaleData(mus, features = all.genes)



# Perform linear dimensional reduction
mus <- RunPCA(mus, features = VariableFeatures(object = mus))

# Examine PCA results
print(mus[["pca"]], dims = 1:5, nfeatures = 5)

VizDimLoadings(mus, dims = 1:2, reduction = "pca")

DimPlot(mus, reduction = "pca")

DimHeatmap(mus, dims = 1, cells = 500, balanced = TRUE)

DimHeatmap(mus, dims = 1:15, cells = 500, balanced = TRUE)

# Determine the dimensionality of the dataset
ElbowPlot(mus) #For choosing number of PCs

# Clustering
mus <- FindNeighbors(mus, dims = 1:20)
mus <- FindClusters(mus, resolution = 0.2)

head(Idents(mus), 5)

mus <- RunUMAP(mus, dims = 1:20)

DimPlot(mus, reduction = "umap")


# DEGs

mus[["RNA"]] <- JoinLayers(mus[["RNA"]])
mus.markers <- FindAllMarkers(mus, only.pos = TRUE, min.pct = 0.25, logfc.threshold = 0.25)
write.csv(mus.markers, "MarkerGenes_AllCellsRes0.2.csv")



# Assessing stressed cell clusters

genes=c("Egr1", "Fos", "Jun", "Atf3","Hbegf")

DotPlot(object=mus,assay="RNA",features=genes)

genes.dissoc <- list(c("Atf3", "Btg2", "Cebpb", "Cebpd", "Cxcl3", "Cxcl2", "Cxcl1", "Dnaja1", "Dnajb1", "Dusp1", "Egr1",
"Fos", "Fosb", "Hsp90aa1", "Hsp90ab1", "Hspa1a", "Hspa1b", "Hspa1a", "Hspa1b", "Hspa8", "Hspb1", "Hspe1", "Hsph1", "Id3",
"Ier2", "Jun", "Junb", "Jund", "Nfkbia", "Nr4a1", "Ppp1r15a", "Socs3", "Zfp36"))

mus <- AddModuleScore(mus, features = genes.dissoc, ctrl= 20, name = 'stressed')

FeaturePlot(mus, features = 'stressed1')
```

```
# Remove stressed cells

mus <- subset(mus,idents=c("0","2","3","4","6","7","8","9","10","11"))
mus
#Perform linear dimensional reduction
mus <- RunPCA(mus, features = VariableFeatures(object = mus))

# Examine PCA results
print(mus[["pca"]], dims = 1:5, nfeatures = 5)

VizDimLoadings(mus, dims = 1:2, reduction = "pca")

DimPlot(mus, reduction = "pca")

DimHeatmap(mus, dims = 1, cells = 500, balanced = TRUE)

DimHeatmap(mus, dims = 1:15, cells = 500, balanced = TRUE)

#Determine the dimensionality of the dataset
ElbowPlot(mus) #For choosing number of PCs

#Clustering
mus <- FindNeighbors(mus, dims = 1:20)
mus <- FindClusters(mus, resolution = 0.1)

head(Idents(mus), 5)

mus <- RunUMAP(mus, dims = 1:20)

DimPlot(mus, reduction = "umap")


#Identify epithelial clusters

VlnPlot(mus, features = c("Epcam"))

#Identify immune clusters

VlnPlot(mus, features = c("Ptprc"))



# Subset immune cells
musImmune <- subset(mus,idents=c("5","8","9"))
musImmune

#Perform linear dimensional reduction
musImmune <- RunPCA(musImmune, features = VariableFeatures(object = musImmune))

# Examine and visualize PCA results
print(musImmune[["pca"]], dims = 1:5, nfeatures = 5)

VizDimLoadings(musImmune, dims = 1:2, reduction = "pca")

DimPlot(musImmune, reduction = "pca")

DimHeatmap(musImmune, dims = 1, cells = 500, balanced = TRUE)

DimHeatmap(musImmune, dims = 1:15, cells = 500, balanced = TRUE)

#Determine the dimensionality of the dataset
ElbowPlot(musImmune) #For choosing number of PCs

#Clustering
musImmune <- FindNeighbors(musImmune, dims = 1:20)
musImmune <- FindClusters(musImmune, resolution = 0.3)

head(Idents(musImmune), 5)

musImmune <- RunUMAP(musImmune, dims = 1:20)

DimPlot(musImmune, reduction = "umap")

#Dotplot
genes=c("Plac8","Axl","Mrc1", "Gzma", "Trgc1", "Lef1", "S100a9","Igkc","Itga1","Cd209a")
DotPlot(object=musImmune,assay="RNA",features=genes)

genes=c("Fcgr3","Cd3e","Cd4", "Il23r")
DotPlot(object=musImmune,assay="RNA",features=genes)

#Find markers
musImmune.markers <- FindAllMarkers(musImmune, assay="RNA", only.pos = TRUE, min.pct = 0.25, logfc.threshold = 0.25)
write.csv(musImmune.markers, "MarkerGenes_ImmuneRes0.3.csv")

#Save Seurat object
saveRDS(musImmune, file = "musImmune.rds")
```

```
musUr=readRDS(file = "musUrImmune.rds")



mus_list=list(musBl, musUr)

# normalize and identify variable features for each dataset independently
mus_list <- lapply(X = mus_list, FUN = function(x) {
  x <- NormalizeData(x)
  x <- FindVariableFeatures(x, selection.method = "vst", nfeatures = 2000)
})

# Select features that are repeatedly variable across datasets for integration
features <- SelectIntegrationFeatures(object.list = mus_list)

Immune.anchors <- FindIntegrationAnchors(object.list = mus_list, anchor.features = features)

#Creating the integrated data assay
musImmune <- IntegrateData(anchorset = Immune.anchors)

#Integrated analysis on combined cells

# specify that we will perform downstream analysis on the corrected data note that the
# original unmodified data still resides in the 'RNA' assay
DefaultAssay(musImmune) <- "integrated"

# Run the standard workflow for visualization and clustering
musImmune <- ScaleData(musImmune, verbose = FALSE)
musImmune <- RunPCA(musImmune, npcs = 30, verbose = FALSE)
musImmune <- RunUMAP(musImmune, reduction = "pca", dims = 1:30)
musImmune <- FindNeighbors(musImmune, reduction = "pca", dims = 1:30)
musImmune <- FindClusters(musImmune, resolution = 0.5)

DimPlot(musImmune, reduction = "umap")

DimPlot(musImmune, reduction = "umap", group.by = "orig.ident")

saveRDS(musImmune, file = "mus_ImmuneBlUr.rds")
mus=musImmune

#mus=readRDS(file = "mus_ImmuneBlUr.rds")
names(mus@meta.data)
Idents(object=mus)<-"integrated_snn_res.0.5"
print(levels(mus))

DefaultAssay(mus) <- "integrated"

#Clustering
mus <- FindNeighbors(mus, dims = 1:30)
mus <- FindClusters(mus, resolution = 0.1)

head(Idents(mus), 5)

mus <- RunUMAP(mus, dims = 1:30)

DimPlot(mus, reduction = "umap")

VlnPlot(mus, features = c("Cx3cr1"))

mus=musImmune

#Determine the dimensionality of the dataset
ElbowPlot(musImmune) #For choosing number of PCs

#Clustering
musImmune <- FindNeighbors(musImmune, dims = 1:20)
musImmune <- FindClusters(musImmune, resolution = 0.2)

head(Idents(musImmune), 5)

musImmune <- RunUMAP(musImmune, dims = 1:20)

saveRDS(musImmune, file = "musImmune.rds")

musImmune=readRDS(file = "musImmune.rds")

##VISUALIZATION
DimPlot(musImmune, reduction = "umap")

DimPlot(musImmune, reduction = "umap", group.by = "orig.ident")

##FEATURE PLOTS
FeaturePlot(musImmune, features = c("Cx3cr1"))
```

```
##VIOLIN PLOT
VlnPlot(musImmune, features = c("Adgre1"))

musImmune[["RNA"]] <- JoinLayers(musImmune[["RNA"]])
musImmune.markers <- FindAllMarkers(musImmune, assay="RNA", only.pos = TRUE, min.pct = 0.25, logfc.threshold = 0.25)
write.csv(musImmune.markers, "ImmuneMarkersRes0.2.csv")


#Subset Myeloid cells
musMyeloid <- subset(musImmune,idents=c("0","2","6","10","11","12"))

#Perform linear dimensional reduction
musMyeloid <- RunPCA(musMyeloid, features = VariableFeatures(object = musMyeloid))

# Examine and visualize PCA results a few different ways
print(musMyeloid[["pca"]], dims = 1:5, nfeatures = 5)

VizDimLoadings(musMyeloid, dims = 1:2, reduction = "pca")

DimPlot(musMyeloid, reduction = "pca")

DimHeatmap(musMyeloid, dims = 1, cells = 500, balanced = TRUE)

DimHeatmap(musMyeloid, dims = 1:15, cells = 500, balanced = TRUE)

#Determine the dimensionality of the dataset
ElbowPlot(musMyeloid) #For choosing number of PCs

#Clustering
musMyeloid <- FindNeighbors(musMyeloid, dims = 1:15)
musMyeloid <- FindClusters(musMyeloid, resolution = 0.6)

head(Idents(musMyeloid), 5)

musMyeloid <- RunUMAP(musMyeloid, dims = 1:15)

saveRDS(musMyeloid, file = "musMyeloid.rds")


##VISUALIZATION
DimPlot(musMyeloid, reduction = "umap")

DimPlot(musMyeloid, reduction = "umap", group.by = "orig.ident")

##FEATURE PLOTS
FeaturePlot(musMyeloid, features = c("Retnla"))

##VIOLIN PLOT
VlnPlot(musMyeloid, features = c("Cx3cr1"))

musMyeloid[["RNA"]] <- JoinLayers(musMyeloid[["RNA"]])
musMyeloid.markers <- FindAllMarkers(musMyeloid, assay="RNA", only.pos = TRUE, min.pct = 0.25, logfc.threshold = 0.25)
write.csv(musMyeloid.markers, "MyeloidMarkersRes0.6.csv")

head(musMyeloid@meta.data)

cellnumbers=table(musMyeloid@meta.data$integrated_snn_res.0.6, musMyeloid@meta.data$orig.ident)
write.csv(cellnumbers,"cellnumbers.csv")


#########################Bladder and urethra epithelial integration and clustering#####################
#################################################################################################

# Load datasets for UR1  Seurat object GSE293686

mus.UR1=Read10X_h5("UR_1_filtered_feature_bc_matrix.h5", use.names = TRUE, unique.features=TRUE)
mus.UR1=CreateSeuratObject(counts = mus.UR1, project = "UR1")
mus.UR1

#Bladder dataset from GSE129845 (GSM3723360     Mice Bladder Homogenate Reaction 1). Unzip files and transfer
#barcodes, genes and and matrix files to a directory

data_dir <- 'C:/Users/HP/Desktop/Immune manuscript/MouseBladderEpi2019_Seurat/Bl1' #change to directory path
list.files(data_dir) # Should show barcodes.tsv, genes.tsv, and matrix.mtx
expression_matrix <- Read10X(data.dir = data_dir)
musBl1 = CreateSeuratObject(counts = expression_matrix)


mus_list=list(musBl1, mus.UR1)

# normalize and identify variable features for each dataset independently
mus_list <- lapply(X = mus_list, FUN = function(x) {
  x <- NormalizeData(x)
  x <- FindVariableFeatures(x, selection.method = "vst", nfeatures = 2000)
})
```

```
# Select features that are repeatedly variable across datasets for integration
features <- SelectIntegrationFeatures(object.list = mus_list)

BlUr.anchors <- FindIntegrationAnchors(object.list = mus_list, anchor.features = features)


musBlUr <- IntegrateData(anchorset = BlUr.anchors)

#Integrated analysis on combined cells

# specify that we will perform downstream analysis on the corrected data note that the
# original unmodified data still resides in the 'RNA' assay
DefaultAssay(musBlUr) <- "integrated"

# Run the standard workflow for visualization and clustering
musBlUr <- ScaleData(musBlUr, verbose = FALSE)
musBlUr <- RunPCA(musBlUr, npcs = 30, verbose = FALSE)
musBlUr <- RunUMAP(musBlUr, reduction = "pca", dims = 1:30)
musBlUr <- FindNeighbors(musBlUr, reduction = "pca", dims = 1:30)
musBlUr <- FindClusters(musBlUr, resolution = 0.5)

DimPlot(musBlUr, reduction = "umap")

DimPlot(musBlUr, reduction = "umap", group.by = "orig.ident")

#Determine the dimensionality of the dataset
ElbowPlot(musBlUr) #For choosing number of PCs

#Clustering
musBlUr <- FindNeighbors(musBlUr, dims = 1:20)
musBlUr <- FindClusters(musBlUr, resolution = 0.2)

head(Idents(musBlUr), 5)

musBlUr <- RunUMAP(musBlUr, dims = 1:20)

DimPlot(musBlUr, reduction = "umap")
DimPlot(musBlUr, reduction = "umap", group.by = "orig.ident")

##VIOLIN PLOT
VlnPlot(musBlUr, features = c("Cxcl17"))

##DOTPLOT
genes=c("Fosb", "Hbegf","Fosl1","Myc")
DotPlot(object=musBlUr,assay="RNA",features=genes)

#Remove stressed cluster 2

musBlUr <- subset(musBlUr,idents=c("0","1","3","4","5","6","7","8","9","10","11","12","13"))

#Clustering
musBlUr <- FindNeighbors(musBlUr, dims = 1:20)
musBlUr <- FindClusters(musBlUr, resolution = 0.2)

head(Idents(musBlUr), 5)

musBlUr <- RunUMAP(musBlUr, dims = 1:20)

DimPlot(musBlUr, reduction = "umap")
DimPlot(musBlUr, reduction = "umap", group.by = "orig.ident")

##VIOLIN PLOT
VlnPlot(musBlUr, features = c("Epcam"))


#Subset Epi
musEpi <- subset(musBlUr,idents=c("0","1","2","5","6","7","10","12"))

#Perform linear dimensional reduction
musEpi <- RunPCA(musEpi, features = VariableFeatures(object = musEpi))

# Examine and visualize PCA results a few different ways
print(musEpi[["pca"]], dims = 1:5, nfeatures = 5)

VizDimLoadings(musEpi, dims = 1:2, reduction = "pca")

DimPlot(musEpi, reduction = "pca")

DimHeatmap(musEpi, dims = 1, cells = 500, balanced = TRUE)

DimHeatmap(musEpi, dims = 1:15, cells = 500, balanced = TRUE)

#Determine the dimensionality of the dataset
ElbowPlot(musEpi) #For choosing number of PCs

#Clustering
```

```
musEpi <- FindNeighbors(musEpi, dims = 1:20)
musEpi <- FindClusters(musEpi, resolution = 0.2)

head(Idents(musEpi), 5)

musEpi <- RunUMAP(musEpi, dims = 1:20)

##VISUALIZATION
DimPlot(musEpi, reduction = "umap")

DimPlot(musEpi, reduction = "umap", group.by = "orig.ident")

##VIOLIN PLOT
VlnPlot(musEpi, features = c("Slit3"))

FeaturePlot(musEpi, features = c("Cxcl17"))

saveRDS(musEpi, file = "musEpi.rds")

musEpi=readRDS(file = "musEpi.rds")

musEpi[["RNA"]] <- JoinLayers(musEpi[["RNA"]])
musEpi.markers <- FindAllMarkers(musEpi, assay="RNA", only.pos = TRUE, min.pct = 0.25, logfc.threshold = 0.25)
write.csv(musEpi.markers, "EpiMarkersRes0.2.csv")


##DOTPLOT
genes=c("Krt4", "Krt18","Lgals7","Upk3a","Cxcl17", "Vcl","Mki67","Vim","Myh11")
DotPlot(object=musEpi,assay="RNA",features=genes)


genes=c("Cxcl17","Nupr1","Pigr","Clu","Krt4","Upk1a","Krt20","Upk2","Upk3a","Sprr2a3","Fabp5")
DotPlot(object=musEpi,assay="RNA",features=genes)

########################Female mouse urethra PBS vs LPS: clustering##############################################
#####################################################################################################

# Load datasets for UrPBS and UrLPS from GSE304686 into Seurat objects

mus.PBS=Read10X_h5("PBSfiltered_feature_bc_matrix.h5", use.names = TRUE, unique.features=TRUE)
mus.PBS=CreateSeuratObject(counts = mus.PBS, project = "PBS")
mus.PBS

mus.LPS=Read10X_h5("LPSfiltered_feature_bc_matrix.h5", use.names = TRUE, unique.features=TRUE)
mus.LPS=CreateSeuratObject(counts = mus.LPS, project = "LPS")
mus.LPS

# Combining the two Seurat objects
mus=merge(mus.PBS, y = mus.LPS, add.cell.ids = c("PBS", "LPS"), project = "UrPBSLPS")
mus
head(colnames(mus))
table(mus$orig.ident)

# Get percentage mito genes
mus[["percent.mt"]] <- PercentageFeatureSet(mus, pattern = "^mt-")

# Visualize QC metrics as a violin plot
VlnPlot(mus, features = c("nFeature_RNA", "nCount_RNA", "percent.mt"), ncol = 3)

# Filtering by nFeature and percent.mt
mussubset <- subset(mus, subset = nFeature_RNA > 200 & nFeature_RNA < 6000 & percent.mt < 20)
mussubset
mus=mussubset


# Normalizing
mus <- NormalizeData(mus, normalization.method = "LogNormalize", scale.factor = 10000)

# Identification of highly variable features (feature selection)
mus <- FindVariableFeatures(mus, selection.method = "vst", nfeatures = 2000)

# Identify the 10 most highly variable genes
top10 <- head(VariableFeatures(mus), 10)

# Plot variable features with and without labels
plot1 <- VariableFeaturePlot(mus)
plot2 <- LabelPoints(plot = plot1, points = top10, repel = TRUE)
plot1
plot2


# Scaling the data so that highly-expressed genes do not dominate
all.genes <- rownames(mus)
mus <- ScaleData(mus, features = all.genes)
```

```
# Perform linear dimensional reduction
mus <- RunPCA(mus, features = VariableFeatures(object = mus))

# Examine PCA results
print(mus[["pca"]], dims = 1:5, nfeatures = 5)

VizDimLoadings(mus, dims = 1:2, reduction = "pca")

DimPlot(mus, reduction = "pca")

DimHeatmap(mus, dims = 1, cells = 500, balanced = TRUE)

DimHeatmap(mus, dims = 1:15, cells = 500, balanced = TRUE)

# Determine the dimensionality of the dataset
ElbowPlot(mus) #For choosing number of PCs

# Clustering
mus <- FindNeighbors(mus, dims = 1:20)
mus <- FindClusters(mus, resolution = 0.2)

head(Idents(mus), 5)

mus <- RunUMAP(mus, dims = 1:20)

DimPlot(mus, reduction = "umap")

DimPlot(mus, reduction = "umap", group.by="orig.ident")

table(mus@meta.data$RNA_snn_res.0.2)

# DEGs

mus[["RNA"]] <- JoinLayers(mus[["RNA"]])
mus.markers <- FindAllMarkers(mus, only.pos = TRUE, min.pct = 0.25, logfc.threshold = 0.25)
write.csv(mus.markers, "MarkerGenes_AllCellsRes0.2.csv")


#Identify immune clusters

VlnPlot(mus, features = c("Ptprc"))

# Subset immune cells
musImmune <- subset(mus,idents=c("1","2","5","8","10"))
musImmune

#Perform linear dimensional reduction
musImmune <- RunPCA(musImmune, features = VariableFeatures(object = musImmune))

# Examine and visualize PCA results
print(musImmune[["pca"]], dims = 1:5, nfeatures = 5)

VizDimLoadings(musImmune, dims = 1:2, reduction = "pca")

DimPlot(musImmune, reduction = "pca")

DimHeatmap(musImmune, dims = 1, cells = 500, balanced = TRUE)

DimHeatmap(musImmune, dims = 1:15, cells = 500, balanced = TRUE)

#Determine the dimensionality of the dataset
ElbowPlot(musImmune) #For choosing number of PCs

#Clustering
musImmune <- FindNeighbors(musImmune, dims = 1:15)
musImmune <- FindClusters(musImmune, resolution = 0.3)

head(Idents(musImmune), 5)

musImmune <- RunUMAP(musImmune, dims = 1:15)

DimPlot(musImmune, reduction = "umap")
DimPlot(musImmune, reduction = "umap", group.by="orig.ident")

table(musImmune@meta.data$RNA_snn_res.0.3, musImmune@meta.data$orig.ident)

#Feature plots
FeaturePlot(musImmune, features = c("Cd74"))

#Violin plots
VlnPlot(musImmune, features = c("Adgre1"))

#Ridge plots
RidgePlot(object=musImmune,features="Cd8a")

#Heatmap
```

```
genes=c("Mrc1", "Adgre1", "Cd3e", "Cd8","Cd4", "Cd19")
heatmap <- DoHeatmap(object=musImmune,features=genes,size=3,angle=22.5)
heatmap <- heatmap+theme(legend.text=element_text(size=10),legend.title=element_text(size=10))
heatmap

#Dotplot
genes=c("Plac8","Axl","Mrc1", "Gzma", "Trgc1", "Lef1", "S100a9","Igkc","Itga1","Cd209a")
DotPlot(object=musImmune,assay="RNA",features=genes)

genes=c("Fcgr3","Cd3e","Cd4", "Il23r")
DotPlot(object=musImmune,assay="RNA",features=genes)

#Find markers
musImmune[["RNA"]] <- JoinLayers(musImmune[["RNA"]])
musImmune.markers <- FindAllMarkers(musImmune, assay="RNA", only.pos = TRUE, min.pct = 0.25, logfc.threshold = 0.25)
write.csv(musImmune.markers, "MarkerGenes_ImmuneRes0.3.csv")

#Save Seurat object
saveRDS(musImmune, file = "musImmune.rds")

musImmune=readRDS(file = "musImmune.rds")



# Subset resident myeloid cells

musMyeloid <- subset(musImmune,idents=c("3","5","9"))

#Perform linear dimensional reduction
musMyeloid <- RunPCA(musMyeloid, features = VariableFeatures(object = musMyeloid))

# Examine and visualize PCA results a few different ways
print(musMyeloid[["pca"]], dims = 1:5, nfeatures = 5)

VizDimLoadings(musMyeloid, dims = 1:2, reduction = "pca")

DimPlot(musMyeloid, reduction = "pca")

DimHeatmap(musMyeloid, dims = 1, cells = 500, balanced = TRUE)

DimHeatmap(musMyeloid, dims = 1:15, cells = 500, balanced = TRUE)

#Determine the dimensionality of the dataset
ElbowPlot(musMyeloid) #For choosing number of PCs

#Clustering
musMyeloid <- FindNeighbors(musMyeloid, dims = 1:15)
musMyeloid <- FindClusters(musMyeloid, resolution = 0.2)

table(musMyeloid@meta.data$RNA_snn_res.0.2)
table(musMyeloid@meta.data$RNA_snn_res.0.2, musMyeloid@meta.data$orig.ident)

head(Idents(musMyeloid), 5)

musMyeloid <- RunUMAP(musMyeloid, dims = 1:15)

DimPlot(musMyeloid, reduction = "umap")
DimPlot(musMyeloid, reduction = "umap", group.by="orig.ident")


table(musMyeloid@meta.data$RNA_snn_res.0.2)
table(musMyeloid@meta.data$RNA_snn_res.0.2, musMyeloid@meta.data$orig.ident)


head(musMyeloid@meta.data)
levels(musMyeloid@meta.data$RNA_snn_res.0.2)
Idents(musMyeloid) <- "RNA_snn_res.0.2"

#Find markers
musMyeloid.markers <- FindAllMarkers(musMyeloid, assay="RNA", only.pos = TRUE, min.pct = 0.25, logfc.threshold = 0.25)
write.csv(musMyeloid.markers, "MarkerGenes_MyeloidRes0.2.csv")

#Violin plots
VlnPlot(musMyeloid, features = c("Axl"))
VlnPlot(musMyeloid, features = c("Cxcl16"))
VlnPlot(musMyeloid, features = c("H2-Ab1"))
VlnPlot(musMyeloid, features = c("Cd74"))
VlnPlot(musMyeloid, features = c("Rgs1"))
VlnPlot(musMyeloid, features = c("Tafa2"))

VlnPlot(musMyeloid, features = c("Folr2"))
VlnPlot(musMyeloid, features = c("Mrc1"))
VlnPlot(musMyeloid, features = c("Retnla"))
VlnPlot(musMyeloid, features = c("Lyve1"))
VlnPlot(musMyeloid, features = c("Cd163"))
```

```
musMyeloid$Cluster0.2_treatment=paste(musMyeloid$RNA_snn_res.0.2, musMyeloid$orig.ident, sep = "_")
Idents(musMyeloid) <- "Cluster0.2_treatment"



#MAST for differential expression analysis

install.packages("BiocManager") # Needed to install all Bioconductor packages
BiocManager::install("MAST")

MacA.de <- FindMarkers(musMyeloid, ident.1 = "2_LPS", ident.2 = "2_PBS", test.use = "MAST",verbose = FALSE)
head(MacA.de, n = 10)
write.csv(MacA.de, "DEGenes_MacAMyeloidRes0.2MAST.csv")

MacE.de <- FindMarkers(musMyeloid, ident.1 = "6_LPS", ident.2 = "6_PBS", test.use = "MAST", verbose = FALSE)
head(MacE.de, n = 10)
write.csv(MacE.de, "DEGenes_MacEMyeloidRes0.2MAST.csv")

DC.de <- FindMarkers(musMyeloid, ident.1 = "3_LPS", ident.2 = "3_PBS",test.use = "MAST", verbose = FALSE)
head(DC.de, n = 10)
write.csv(DC.de, "DEGenes_DCMyeloidRes0.2MAST.csv")
```
