# Supplementary material for: Single cell map of the adult female mouse urethra reveals epithelial and stromal macrophages with distinct functional identities
Source: Mucosal Immunol. Author manuscript; Available in PMC 2025 Sep 26. (PMC7618168; doi:10.1016/j.mucimm.2025.09.001)
Supplement: Key resources table [file EMS208696-supplement-Key_resources_table.docx]

**KEY RESOURCES TABLE**

| Reagent or resource | Source | Identifier |
| --- | --- | --- |
| Antibodies | | |
| Rabbit anti-AIF1/IBA-1, mAb, 1:200 dilution (IHC-P) | Cell Signaling Technology | Cat# 17198,  RRID:AB_2820254 |
| Rat anti-CD163, mAb, 1:200 dilution (IHC-Fr) | Invitrogen | Cat# 14-1631-82,  RRID:AB_2716934 |
| Rabbit anti-CD3e, mAb, 1:200 dilution (IHC-P) | Cell Signaling Technology | Cat# 78588,  RRID:AB_2889902 |
| Rabbit anti-CD11c, mAb, 1:200 dilution (IHC-P, IHC-Fr) | Cell Signaling Technology | Cat# 97585,  RRID:AB_2800282 |
| Rabbit anti-CD206, mAb, 1:200 dilution (IHC-P, IHC-Fr) | Cell Signaling Technology | Cat# 24595,  RRID:AB_2892682 |
| Rat anti-CD31, mAb, 1:200 dilution (IHC-Fr) | eBioscience | Cat# 14-0311-81,  RRID:AB_467200 |
| Rabbit anti-CD74, mAb, 1:200 dilution (IHC-P) | Cell Signaling Technology | Cat# 82174S |
| Rabbit anti-CD86, mAb, 1:200 dilution (IHC-Fr) | Cell Signaling Technology | Cat# 19589,  RRID:AB_2892094 |
| Rabbit anti-CDH1, mAb, 1:200 dilution (IHC-Fr, IHC-Wholemount) | Cell Signaling Technology | Cat# 3195S,  RRID:AB_2291471 |
| Mouse anti-CDH1, mAb, 1:200 dilution (IHC-P) | Cell Signaling Technology | Cat#14472,  RRID:AB_2728770 |
| Rabbit anti-CHGA, mAb, 1:200 dilution (IHC-P) | Abcam | Cat# ab254322,  RRID:AB_2910555 |
| Rabbit anti-CLDN10, pAb, 1:200 dilution (IHC-Fr) | BiCell Scientific | Cat# 00210 |
| Rat anti-F4/80, mAb, 1:100 dilution (IHC-Fr) | Invitrogen | Cat# 14-4801-82,  RRID:AB_467558 |
| Rabbit anti-F4/80, mAb, 1:200 dilution (IHC-P) | Cell Signaling Technology | Cat#70076,  RRID:AB_2799771 |
| Chicken anti-KRT5, pAb, 1:800 dilution (IHC-P, IHC-Fr) | Biolegend | Cat# 905901, RRID:AB_2565054 |
| Rat anti-LYVE1, mAb, 1:200 dilution (IHC-Fr) | Invitrogen | Cat# 14-0443-82,  RRID:AB_1633414 |
| Rat anti-MGL2, mAb, 1:200 dilution (IHC-Fr) | Invitrogen | Cat# 14-3011-80,  RRID:AB_2572871 |
| Rat anti-MHCII, mAb, 1:200 dilution (IHC-P) | Invitrogen | Cat# 14-5321-82,  RRID:AB_467561 |
| Mouse anti-MHCII, mAb, 1:200 dilution (IHC-P) | Abcam | Cat# ab23990,  RRID:AB_447796 |
| Mouse anti-Pan Cytokeratin, mAb, 1:200 dilution (IHC-P) | Cell Signaling Technology | Cat# 4545,  RRID:AB_490860 |
| Donkey Anti-Chicken IgG (H+L) Alexa Fluor 488, 1:500 dilution | Jackson Immunoresearch | Cat# 703-545-155, RRID:AB_2340375 |
| Donkey Anti-Mouse IgG (H+L) Alexa Fluor 488, 1:500 dilution | Jackson Immunoresearch | Cat# 715-545-151, RRID:AB_2341099 |
| Donkey Anti-Mouse IgG (H+L) Alexa Fluor 594, 1:500 dilution | Jackson Immunoresearch | Cat# 715-585-151, RRID:AB_2340855 |
| Donkey Anti-Mouse IgG (H+L) Alexa Fluor 647, 1:500 dilution | Jackson Immunoresearch | Cat# 715-605-151, RRID:AB_2340863 |
| Donkey Anti-Rabbit IgG (H+L) Alexa Fluor 488, 1:500 dilution | Jackson Immunoresearch | Cat# 711-545-152, RRID:AB_2313584 |
| Donkey Anti-Rabbit IgG (H+L) Alexa Fluor 594, 1:500 dilution | Jackson Immunoresearch | Cat# 715-585-152,  RRID:AB_2492288 |
| Donkey Anti-Rabbit IgG (H+L) Alexa Fluor 647, 1:500 dilution | Jackson Immunoresearch | Cat# 711-605-152, RRID:AB_2340863 |
| Donkey Anti-Rat IgG (H+L) Alexa Fluor 594, 1:500 dilution | Jackson Immunoresearch | Cat# 712-585-153,  RRID:AB_2340689 |
| Donkey Anti-Rat IgG (H+L) Alexa Fluor 647, 1:500 dilution | Jackson Immunoresearch | Cat# 712-605-153, RRID:AB_2340694 |
| Mouse TruStain FcX | Biolegend | Cat# 101320,  RRID:AB_1574975 |
| Live dead dye Ghost V450 | Tonbo Biosciences | Cat# 13-0863-T100 |
| Anti-mouse F4/80, PE conjugated | Tonbo Biosciences | Cat# 50-4801-U100,  RRID:AB_2621795 |
| Anti-mouse CD206, FITC conjugated | Biolegend | Cat# 141704,  RRID:AB_10901166 |
| Anti-mouse CD45, APC conjugated | Tonbo Biosciences | Cat# 20-0451-U100,  RRID:AB_2621573 |
| Bacterial and Virus Strains | | |
|  |  |  |
|  |  |  |
|  |  |  |
|  |  |  |
|  |  |  |
| Biological Samples |  |  |
|  |  |  |
|  |  |  |
|  |  |  |
|  |  |  |
|  |  |  |
| Chemicals, Peptides, and Recombinant Proteins | | |
| Lipopolysaccharides from Escherichia coli O111:B4 | Sigma Aldrich | L2630-10MG |
|  |  |  |
|  |  |  |
|  |  |  |
|  |  |  |
| Critical Commercial Assays | | |
| RNAscope™ Intro Pack 2.5 HD Reagent Kit Brown- Mm | ACD | Cat# 322371 |
| RNAscope Multiplex Fluorescent Reagent kit V2 kit with TSA vivid dyes | ACD | Cat# 323270 |
| RNAscope® Probe-Mm-Csf1r | ACD | Cat# 428191 |
| RNAscope® Probe-Mm-Cx3cl1 | ACD | Cat# 426211 |
| RNAscope® Probe-Mm-Cx3cr1 | ACD | Cat# 314221 |
| RNAscope® Probe-Mm-Cxcl17 | ACD | Cat# 519621 |
| Chromium Next GEM Single Cell 3' Kit v3.1 | 10x Genomics | Cat# 1000269 |
| Chromium Next GEM Chip G | 10x Genomics | Cat# 1000127 |
| Xenium Mouse Tissue Atlassing Panel (379 genes) | 10x Genomics | Cat# 1000627 |
| Illumina SP kit | Illumina | Cat# 20028401 |
| Deposited Data | | |
| Female mouse urethra single cell RNA-sequencing dataset | This paper | GSE293686 |
| Female mouse urethra single cell RNA-sequencing PBS vs LPS treated | This paper | GSE304686 |
| Female mouse urethra Xenium spatial transcriptomics dataset | This paper | GSE296982 |
| Male mouse urethra single cell RNA-sequencing dataset | Joseph DB, Henry GH, Malewska A, Iqbal NS, Ruetten HM, Turco AE, Abler LL, Sandhu SK, Cadena MT, Malladi VS, Reese JC, Mauck RJ, Gahan JC, Hutchinson RC, Roehrborn CG, Baker LA, Vezina CM, Strand DW. Urethral luminal epithelia are castration-insensitive cells of the proximal prostate. Prostate. 2020 Aug;80(11):872-884. doi: 10.1002/pros.24020. Epub 2020 Jun 4. PMID: 32497356; PMCID: PMC7339731. | GSE145865 |
| Mouse bladder immune single cell RNA-sequencing dataset | Ligon MM, Wang C, DeJong EN, Schulz C, Bowdish DME, Mysorekar IU. Single cell and tissue-transcriptomic analysis of murine bladders reveals age- and TNFα-dependent but microbiota-independent tertiary lymphoid tissue formation. Mucosal Immunol. 2020 Nov;13(6):908-918. doi: 10.1038/s41385-020-0290-x. Epub 2020 May 4. PMID: 32366865; PMCID: PMC7572484. | GSE149571 |
| Mouse bladder single cell RNA-sequencing dataset | Yu Z, Liao J, Chen Y, Zou C, Zhang H, Cheng J, Liu D, Li T, Zhang Q, Li J, Yang X, Ye Y, Huang Z, Long X, Yang R, Mo Z. Single-Cell Transcriptomic Map of the Human and Mouse Bladders. J Am Soc Nephrol. 2019 Nov;30(11):2159-2176. doi: 10.1681/ASN.2019040335. Epub 2019 Aug 28. PMID: 31462402; PMCID: PMC6830796. | GSE129845 |
| Experimental Models: Cell Lines | | |
|  |  |  |
|  |  |  |
|  |  |  |
|  |  |  |
|  |  |  |
| Experimental Models: Organisms/Strains | | |
| CD-1 Mice | Charles River, USA | Crl:CD1(ICR) |
| B6.C-Tg(Pgk1-cre)1Lni/CrsJ | The Jackson Laboratory | Strain: 020811 |
| B6.Cg-Csf1rtm1.2Jwp/J | The Jackson Laboratory | Strain: 021212 |
| C57BL6/J | The Jackson Laboratory | Strain: 000664 |
|  |  |  |
|  |  |  |
| Oligonucleotides | | |
| Xenium custom probe details | | |

| Gene | Ensembl ID | Probesets |
| --- | --- | --- |
| Abo | ENSMUSG00000015787 | 8 |
| Acaa1b | ENSMUSG00000010651 | 8 |
| Acta2 | ENSMUSG00000035783 | 3 |
| Apod | ENSMUSG00000022548 | 8 |
| Atp6v1g3 | ENSMUSG00000026394 | 8 |
| Bcam | ENSMUSG00000002980 | 8 |
| Bglap2 | ENSMUSG00000074486 | 6 |
| C1qa | ENSMUSG00000036887 | 8 |
| C1qtnf3 | ENSMUSG00000058914 | 8 |
| Ccl6 | ENSMUSG00000018927 | 8 |
| Cd34 | ENSMUSG00000016494 | 8 |
| Cd79a | ENSMUSG00000003379 | 8 |
| Chga | ENSMUSG00000021194 | 8 |
| Cldn10 | ENSMUSG00000022132 | 8 |
| Cldn5 | ENSMUSG00000041378 | 8 |
| Clu | ENSMUSG00000022037 | 8 |
| Col1a2 | ENSMUSG00000029661 | 5 |
| Crisp1 | ENSMUSG00000025431 | 8 |
| Cxcl14 | ENSMUSG00000021508 | 8 |
| Cyp2f2 | ENSMUSG00000052974 | 8 |
| Cyp4a12a | ENSMUSG00000066071 | 8 |
| Defb42 | ENSMUSG00000054763 | 8 |
| Dmkn | ENSMUSG00000060962 | 8 |
| Dmrt2 | ENSMUSG00000048138 | 8 |
| Egr1 | ENSMUSG00000038418 | 8 |
| Eln | ENSMUSG00000029675 | 8 |
| Fbln1 | ENSMUSG00000006369 | 7 |
| Fcer1g | ENSMUSG00000058715 | 6 |
| Fgl2 | ENSMUSG00000039899 | 8 |
| Flna | ENSMUSG00000031328 | 8 |
| Fosb | ENSMUSG00000003545 | 8 |
| Foxi1 | ENSMUSG00000047861 | 8 |
| Gas1 | ENSMUSG00000052957 | 8 |
| H2-Ab1 | ENSMUSG00000073421 | 4 |
| H2-Eb1 | ENSMUSG00000060586 | 5 |
| Has1 | ENSMUSG00000003665 | 8 |
| Hbegf | ENSMUSG00000024486 | 8 |
| Hhip | ENSMUSG00000064325 | 8 |
| Igf1 | ENSMUSG00000020053 | 8 |
| Iglc1 | ENSMUSG00000105906 | 7 |
| Iglc3 | ENSMUSG00000105547 | 6 |
| Itga6 | ENSMUSG00000027111 | 8 |
| Kcng3 | ENSMUSG00000045053 | 8 |
| Kcnk3 | ENSMUSG00000049265 | 8 |
| Kit | ENSMUSG00000005672 | 8 |
| Klf5 | ENSMUSG00000005148 | 8 |
| Krt14 | ENSMUSG00000045545 | 8 |
| Krt4 | ENSMUSG00000059668 | 8 |
| Krt5 | ENSMUSG00000061527 | 8 |
| Lgals7 | ENSMUSG00000053522 | 8 |
| Lgr5 | ENSMUSG00000020140 | 8 |
| Ly6d | ENSMUSG00000034634 | 8 |
| Lyz1 | ENSMUSG00000069515 | 7 |
| Mcam | ENSMUSG00000032135 | 8 |
| Mki67 | ENSMUSG00000031004 | 8 |
| Mmp7 | ENSMUSG00000018623 | 8 |
| Msln | ENSMUSG00000063011 | 8 |
| Myf5 | ENSMUSG00000000435 | 8 |
| Nkx3-1 | ENSMUSG00000022061 | 8 |
| Pate6 | ENSMUSG00000032108 | 8 |
| Pbsn | ENSMUSG00000000003 | 3 |
| Pdgfrb | ENSMUSG00000024620 | 8 |
| Pecam1 | ENSMUSG00000020717 | 8 |
| Piezo2 | ENSMUSG00000041482 | 8 |
| Pip | ENSMUSG00000058499 | 6 |
| Ppp1r1b | ENSMUSG00000061718 | 8 |
| Psca | ENSMUSG00000022598 | 8 |
| Rbp4 | ENSMUSG00000024990 | 8 |
| Rcan2 | ENSMUSG00000039601 | 8 |
| S100a2 | ENSMUSG00000094018 | 4 |
| Scara5 | ENSMUSG00000022032 | 8 |
| Shh | ENSMUSG00000002633 | 8 |
| Slpi | ENSMUSG00000017002 | 8 |
| Sncg | ENSMUSG00000023064 | 8 |
| Spink5 | ENSMUSG00000055561 | 8 |
| Spink8 | ENSMUSG00000050074 | 8 |
| Srd5a2 | ENSMUSG00000038541 | 8 |
| Stmn2 | ENSMUSG00000027500 | 8 |
| Sult1e1 | ENSMUSG00000029272 | 8 |
| Svs6 | ENSMUSG00000017000 | 6 |
| Tgm4 | ENSMUSG00000025787 | 5 |
| Top2a | ENSMUSG00000020914 | 8 |
| Tph1 | ENSMUSG00000040046 | 8 |
| Tpsab1 | ENSMUSG00000024173 | 8 |
| Trp63 | ENSMUSG00000022510 | 8 |
| Uchl1 | ENSMUSG00000029223 | 8 |
| Upk2 | ENSMUSG00000041523 | 8 |
| Wfdc15b | ENSMUSG00000018211 | 6 |
| Wfdc2 | ENSMUSG00000017723 | 8 |
| Wfdc3 | ENSMUSG00000076434 | 8 |
| Wnt2 | ENSMUSG00000010797 | 8 |
| Pdgfra | ENSMUSG00000029231 | 8 |
| Npy1r | ENSMUSG00000036437 | 8 |
| Lbp | ENSMUSG00000016024 | 8 |
| Htr1a | ENSMUSG00000021721 | 8 |
| Htr3a | ENSMUSG00000032269 | 8 |
| Hcn4 | ENSMUSG00000032338 | 8 |
| Htr2a | ENSMUSG00000034997 | 8 |
| Htr1b | ENSMUSG00000049511 | 8 |
| Kcnmb2 | ENSMUSG00000037610 | 8 |

| Recombinant DNA | | |
| --- | --- | --- |
|  |  |  |
|  |  |  |
|  |  |  |
|  |  |  |
|  |  |  |
| Software and Algorithms | | |
| bcl2fastq | Illumina |  |
| cellranger-8.0.1 | 10x Genomics |  |
| Xenium Explorer 3.1.0 | 10x Genomics |  |
| R statistics software | R project | https://www.r-project.org/ |
| R studio | Posit | https://posit.co/downloads/ |
| Seurat 5.1.0 |  | Satija, R., Farrell, J., Gennert, D. et al. Spatial reconstruction of single-cell gene expression data. Nat Biotechnol 33, 495–502 (2015). https://doi.org/10.1038/nbt.3192 |
| WebGestalt 2024 | https://www.webgestalt.org/ | Elizarraras JM, Liao Y, Shi Z, Zhu Q, Pico AR, Zhang B. WebGestalt 2024: faster gene set analysis and new support for metabolomics and multi-omics. Nucleic Acids Res. 2024 Jul 5;52(W1):W415-W421. doi: 10.1093/nar/gkae456. PMID: 38808672; PMCID: PMC11223849. |
| FCS Express 7 | De Novo Software |  |
| Zen Microscopy software | Zeiss |  |
| Adobe Photoshop | Adobe |  |
| Adobe Illustrator | Adobe |  |
| Other | | |
|  |  |  |
|  |  |  |
|  |  |  |
|  |  |  |
