# Supplementary material for: Single cell map of the adult female mouse urethra reveals epithelial and stromal macrophages with distinct functional identities
Source: Mucosal Immunol. Author manuscript; Available in PMC 2025 Sep 26. (PMC7618168; doi:10.1016/j.mucimm.2025.09.001)
Supplement: Supplementary figures S1-S12 [file EMS208696-supplement-Supplementary_figures_S1_S12.pdf]

Figure S1

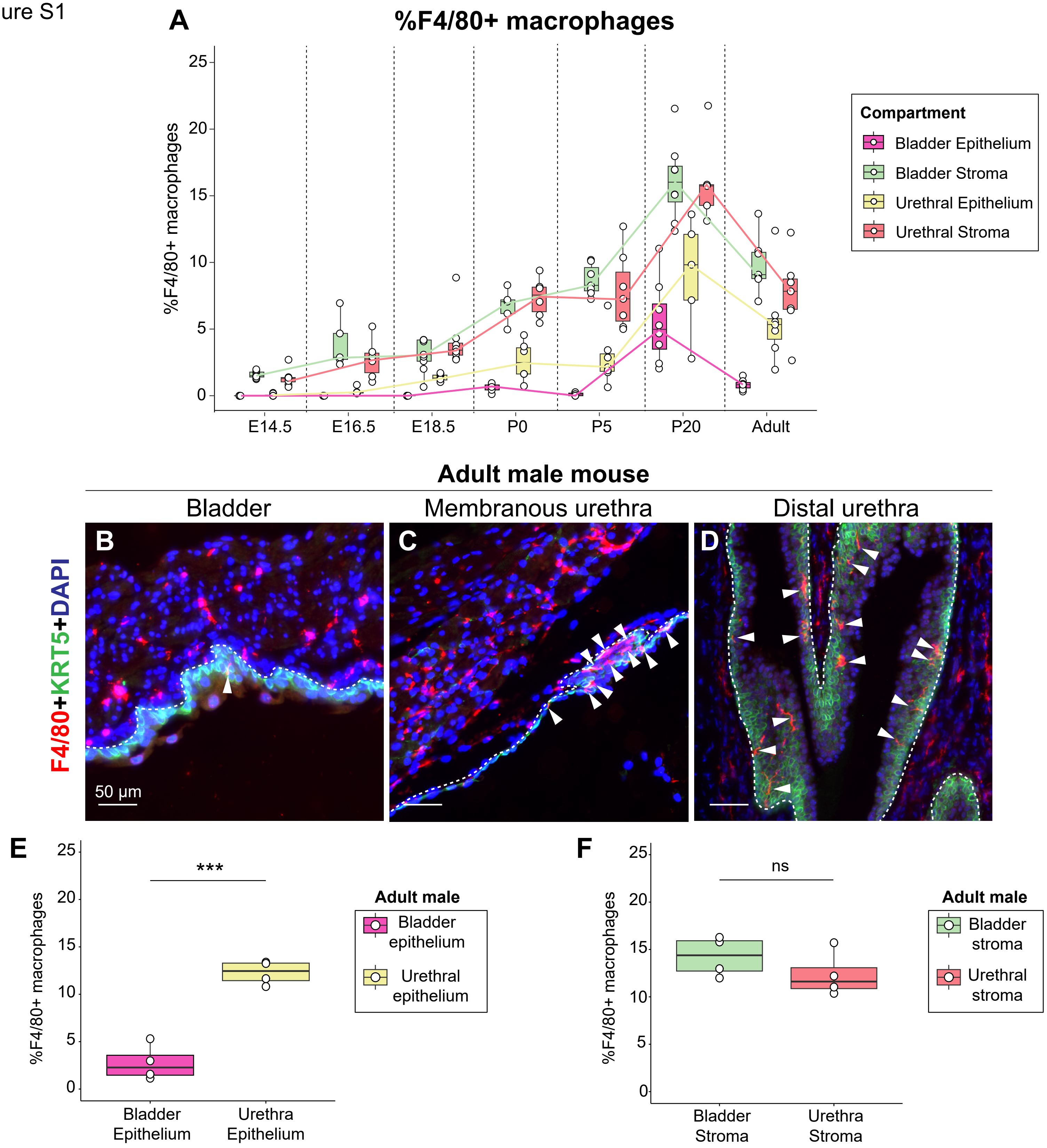


**Figure S1:** (A) Quantification of F4/80+ macrophages in the bladder and urethral epithelium and stroma across different stages. Male and female mice were assessed at E14.5 and E16.5 while only female mice were assessed from E18.5 to Adult stages. Data have been quantified from n=5-8 mice/group from at least n=3 independent litters. Abbreviations: E-embryonic day, P-postnatal day. (B-D) Tissue sections of the adult male mouse bladder and urethra labeled with antibodies to the macrophage marker F4/80 (in red) and the basal epithelial marker KRT5 (in green). Nuclei are labeled in blue. Images are representative of n=4 mice per group. Scale bar represents 50 microns. White arrowheads indicate epithelial-associated macrophages in the urethra. White dotted lines indicate border between epithelium and stroma. (E) Percentage of F4/80+ macrophages in the male bladder and urethral epithelium. (F) Percentage of F4/80+ macrophages in the male bladder and urethral stroma. *** $p < 0.0001$, ns $p > 0.05$, Unpaired Student's t-test.

Figure S2

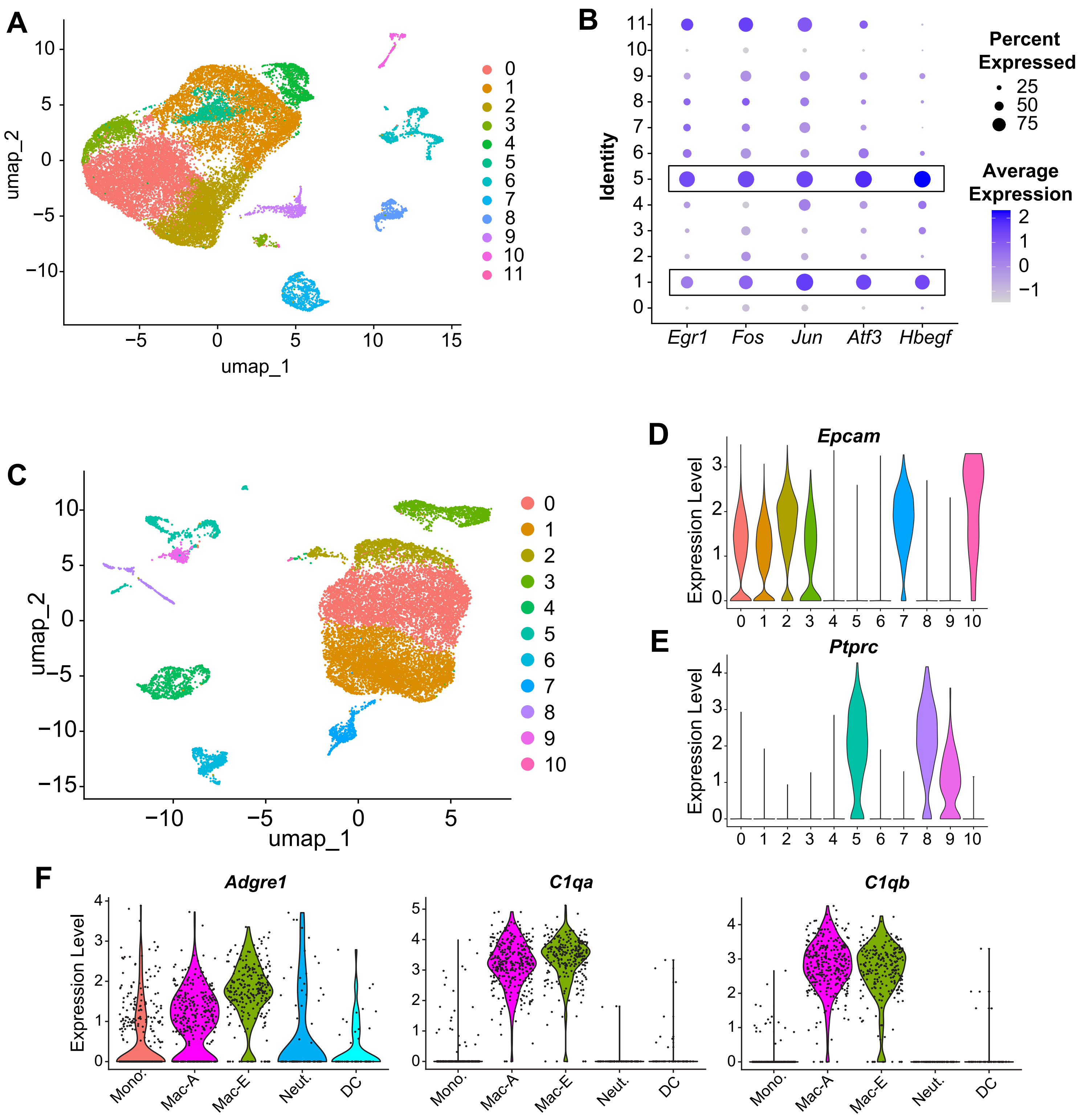


**Figure S2:** (A) UMAP depicting major cell clusters from the female mouse urethra. (B) Dot plot for gene expression for stress related genes showing high expression in clusters 1 and 5. (C) UMAP depicting cell clusters post-filtering and removal of stressed cell clusters. (D) Violin plot for the epithelial gene *Epcam* to identify epithelial cell clusters. (E) Violin plot for the immune cell gene *Ptprc* to identify immune cell clusters. (F) Violin plots showing expression of *Adgre1*, *C1qa* and *C1qb* in the myeloid cell clusters. Abbreviations: Mono.-Monocytes, Mac-A- Macrophage Activated, Mac-E- Macrophage Endocytic, Neut.- Neutrophils, DC- Dendritic cells.

Figure S3

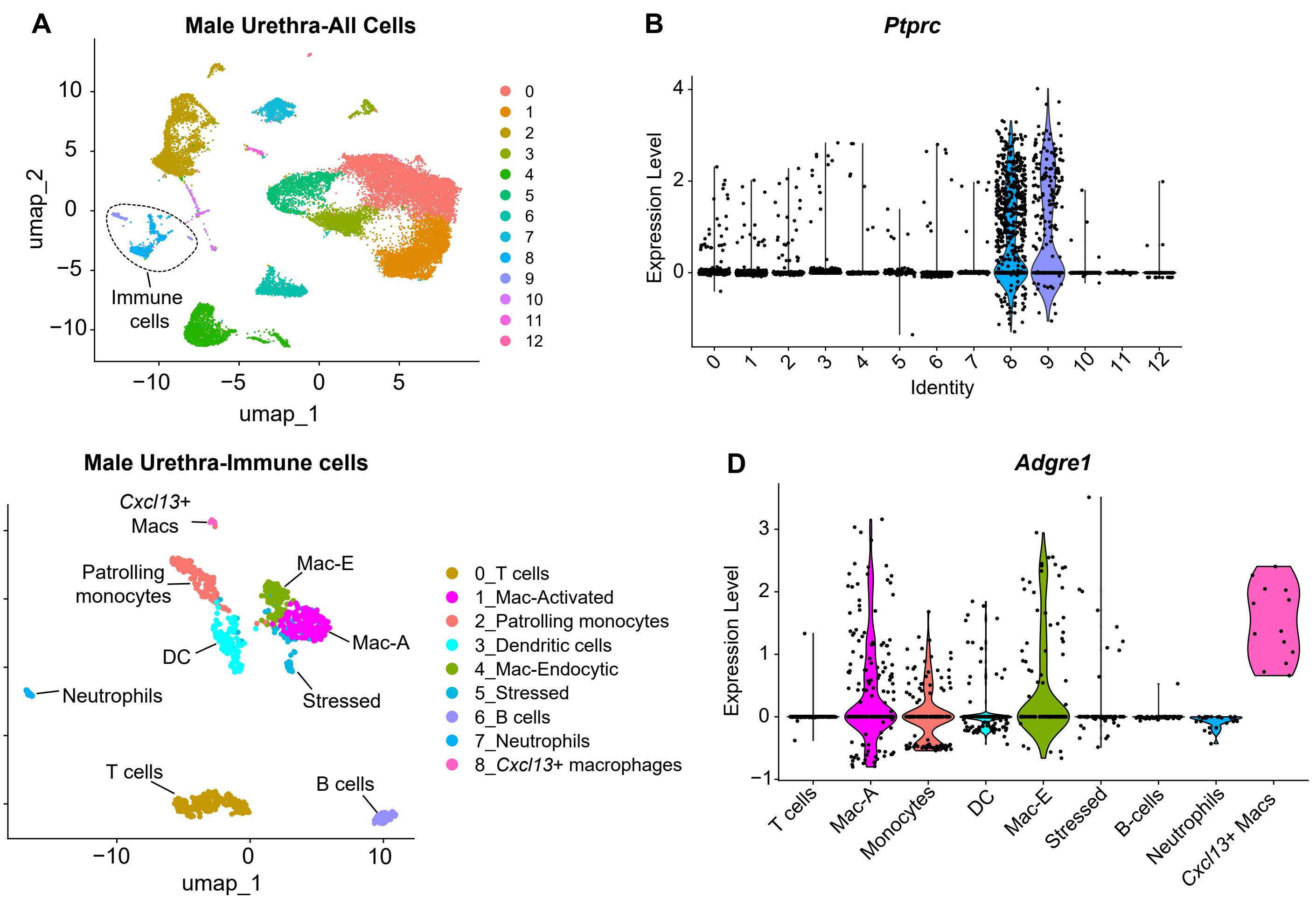


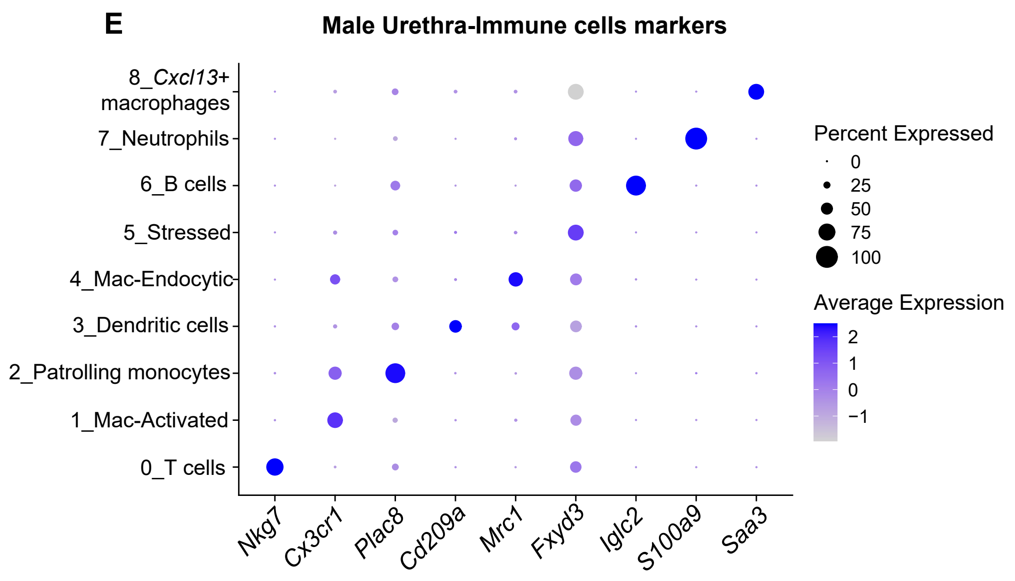


**F** **Top 10 marker genes**
*p-adj (lowest to highest)*
*log2 fold change > 0.5*

| **Mac-A** | **Mac-E** |
|---|---|
| *C1qc* | *Pf4* |
| *C1qb* | *Mrc1* |
| *Ms4a7* | *Mmp9* |
| *C1qa* | *Selenop* |
| *Zmynd15* | *Maf* |
| *Pmepa1* | *Stab1* |
| *Hexb* | *Igfbp4* |
| *Cd74* | *C1qc* |
| *Ctss* | *C1qa* |
| *Cxcl16* | *Cd163* |

**Figure S3:** Single cell RNA-sequencing data from the male proximal urethra (GSE145865) was reanalyzed. (A) UMAP of major cell clusters identified. (B) Violin plot showing expression level of immune cell marker *Ptprc*. (C) Cells with high *Ptprc* expression corresponding to immune cells were further sub-setted and clustered. UMAP of major immune cell clusters is shown. Clusters were annotated using top marker genes. (D) Violin plot showing expression of *Adgre1* gene in immune cell clusters. High *Adgre1* expression is observed in clusters corresponding to Mac-A, Mac-E and Infiltrating macrophages. (E) Dotplot showing expression of marker genes for each immune cell cluster. (F) Top 10 marker genes for Mac-A and Mac-E clusters sorted by p-adjusted lowest to highest (cut-off p-adj < 0.05, log2FC > 0.5).

Figure S4

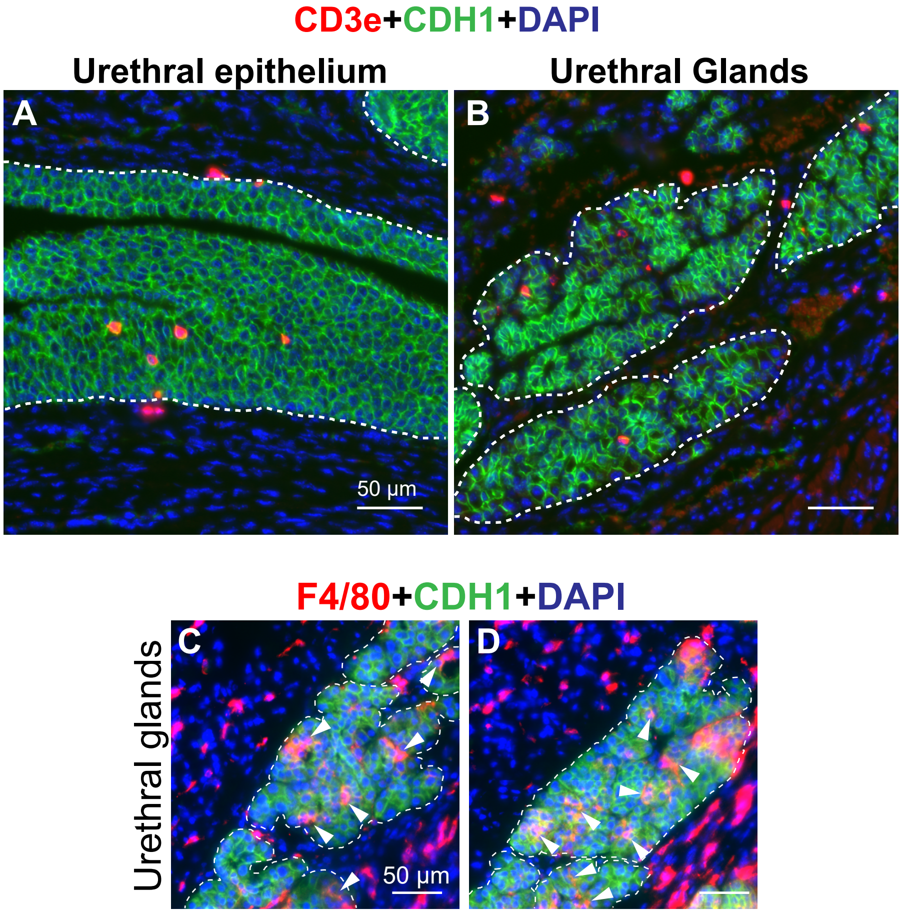


**Figure S4:** 5-micron tissue sections of the adult female mouse urethra labeled with antibodies against the T cell marker CD3e (in red) and the epithelial protein CDH1 (in green). Urethral epithelial regions are displayed in (A) and urethral glands in (B). Images are representative of n=4 mice per group. 5-micron tissue sections of the adult female mouse urethra labeled with antibodies against the macrophage marker F4/80 (in red) and the epithelial protein CDH1 (in green). Urethral glands are displayed in (C) and (D). Nuclei are labeled in blue. Images are representative of n=3 mice per group. Scale bar represents 50 microns. White arrowheads indicate epithelial-associated macrophages in the urethral glands. White dotted line represents border between epithelium and stroma.

Figure S5

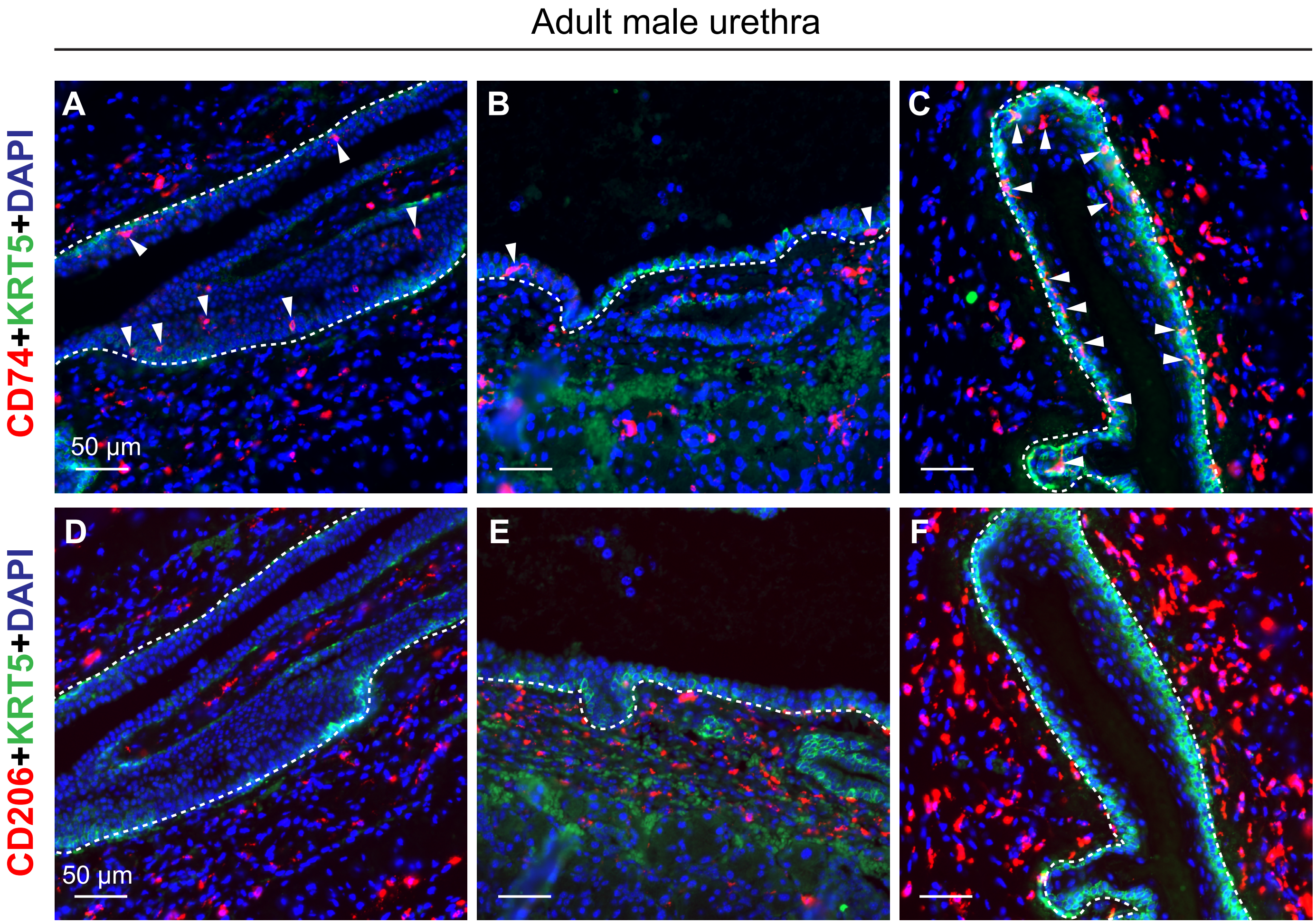


**Figure S5:** (A-C) 5-micron tissue sections of the adult male mouse urethra labeled with antibodies against CD74 (in red) and the basal epithelial marker KRT5 (in green). (D-F) 5-micron tissue sections of the adult male mouse urethra labeled with antibodies against CD206 (in red) and the basal epithelial marker KRT5 (in green). Images are representative of n=4 mice per group. Nuclei are labeled in blue. Scale bar represents 50 microns. White arrowheads indicate epithelial-associated macrophages in the urethral glands. White dotted line represents border between epithelium and stroma.

Figure S6

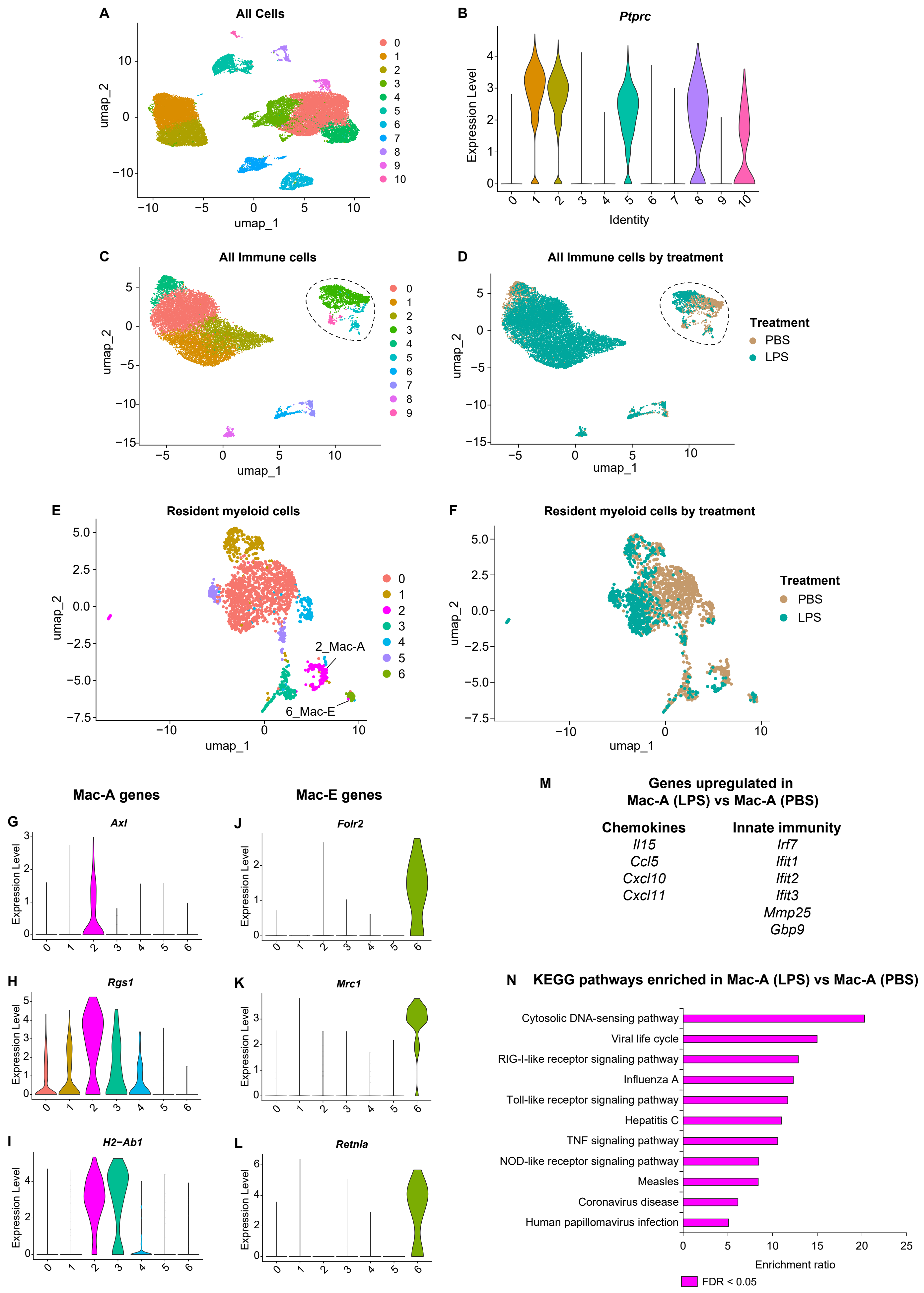
A
All Cells
umap_1
umap_2
B
Ptprc
Expression Level
Identity
C
All Immune cells
D
All Immune cells by treatment
Treatment
PBS
LPS
E
Resident myeloid cells
2_Mac-A
6_Mac-E
F
Resident myeloid cells by treatment
Mac-A genes
Mac-E genes
G
Axl
H
Rgs1
I
H2−Ab1
J
Folr2
K
Mrc1
L
Retnla
M
Genes upregulated in
Mac-A (LPS) vs Mac-A (PBS)
Chemokines
Il15
Ccl5
Cxcl10
Cxcl11
Innate immunity
Irf7
Ifit1
Ifit2
Ifit3
Mmp25
Gbp9
N
KEGG pathways enriched in Mac-A (LPS) vs Mac-A (PBS)
Cytosolic DNA-sensing pathway
Viral life cycle
RIG-I-like receptor signaling pathway
Influenza A
Toll-like receptor signaling pathway
Hepatitis C
TNF signaling pathway
NOD-like receptor signaling pathway
Measles
Coronavirus disease
Human papillomavirus infection
Enrichment ratio
FDR < 0.05

**Figure S6: Transcriptional changes in resident Mac-A cells upon LPS treatment.** Adult female mice were transurethrally instilled with sterile PBS or sterile PBS containing 1 mg/ml lipopolysaccharides (LPS). Mice were euthanized 3 hours post-instillation and 3 mice per group were pooled for the PBS and LPS treated samples. (A) UMAP depicting major cell clusters from the female mouse urethras of mice treated with PBS and LPS. (B) Violin plot depicting expression of the immune cell marker *Ptprc* to identify immune cell clusters. (C) UMAP of immune cells from the PBS and LPS treated mouse urethras showing major immune cell clusters. Resident myeloid cell clusters are within the dotted line. (D) UMAP of immune cells from PBS and LPS treated mouse urethras labelled by treatment type. (E) UMAP of resident myeloid cells from PBS and LPS treated mouse urethras. (F) UMAP of resident myeloid cells from PBS and LPS treated mouse urethras labelled by treatment type. Violin plots indicating expression of Mac-A markers (G) *Axl* (H) *Rgs1* and (I) *H2-Ab1* in resident myeloid cell clusters. Violin plots indicating expression of Mac-E markers (J) *Folr2* (K) *Mrc1* and (L) *Retnla* in resident myeloid cell clusters. (M) Selected genes upregulated in the Mac-A cluster upon LPS treatment (log2FC > 0.5 and padj $\leq$ 0.1). (N) KEGG pathways enriched in Mac-A cluster upon LPS treatment.

Figure S7

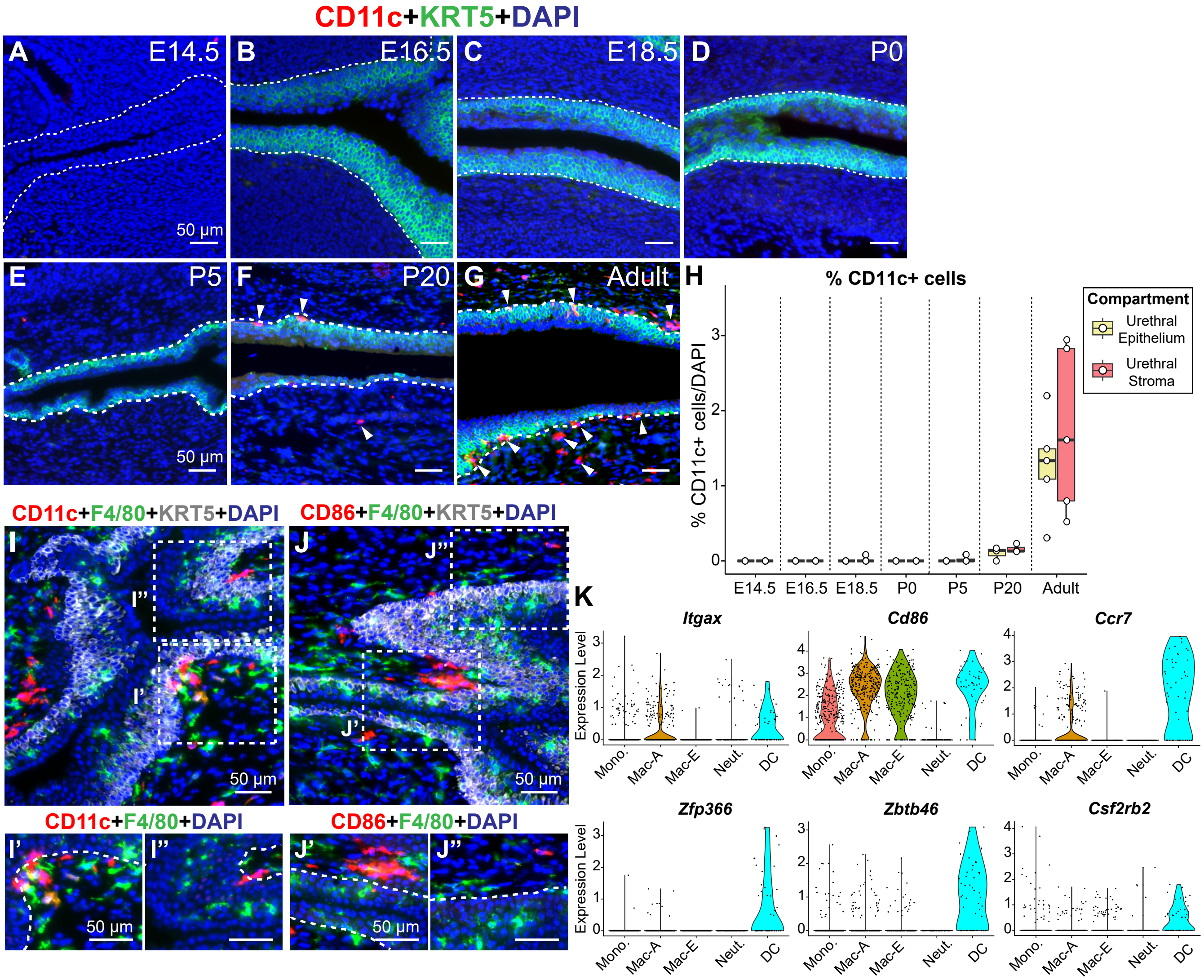


**Figure S7:** Tissue sections of urethras from (A) E14.5 mice, (B) E16.5 mice, (C) E18.5 female mice, (D) P0 female mice, (E) P5 female mice, (F) P20 female mice and (G) adult female mice labeled with antibodies to CD11c (in red) and KRT5 (in green). White arrowheads indicate dendritic cells expressing high levels of CD11c. (H) Quantification of CD11c high cells in the urethral epithelium and urethral stroma across different stages. Images are representative of at least n=3 mice/group from at least n=3 independent litters. (I) Tissue sections of adult female mouse urethra labeled with antibodies to CD11c (in red), F4/80 (in green) and KRT5 (in white). Magnified insets from (I) shown in I' and I". (J) Tissue sections of adult female mouse urethra labeled with antibodies to CD86 (in red), F4/80 (in green) and KRT5 (in white). Magnified insets from (J) shown in J' and J". Nuclei are labeled in blue. Scale bar represents 50 microns. White dotted line indicates border between epithelium and stroma. (K) Violin plots showing expression of *Itgax, Cd86, Ccr7, Zfp366, Zbtb46* and *Csf2rb2* in myeloid cell clusters. Abbreviations: Mono.-Monocytes, Mac-A- Macrophage Activated, Mac-E- Macrophage Endocytic, Neut.- Neutrophils, DC- Dendritic cells.

Figure S8

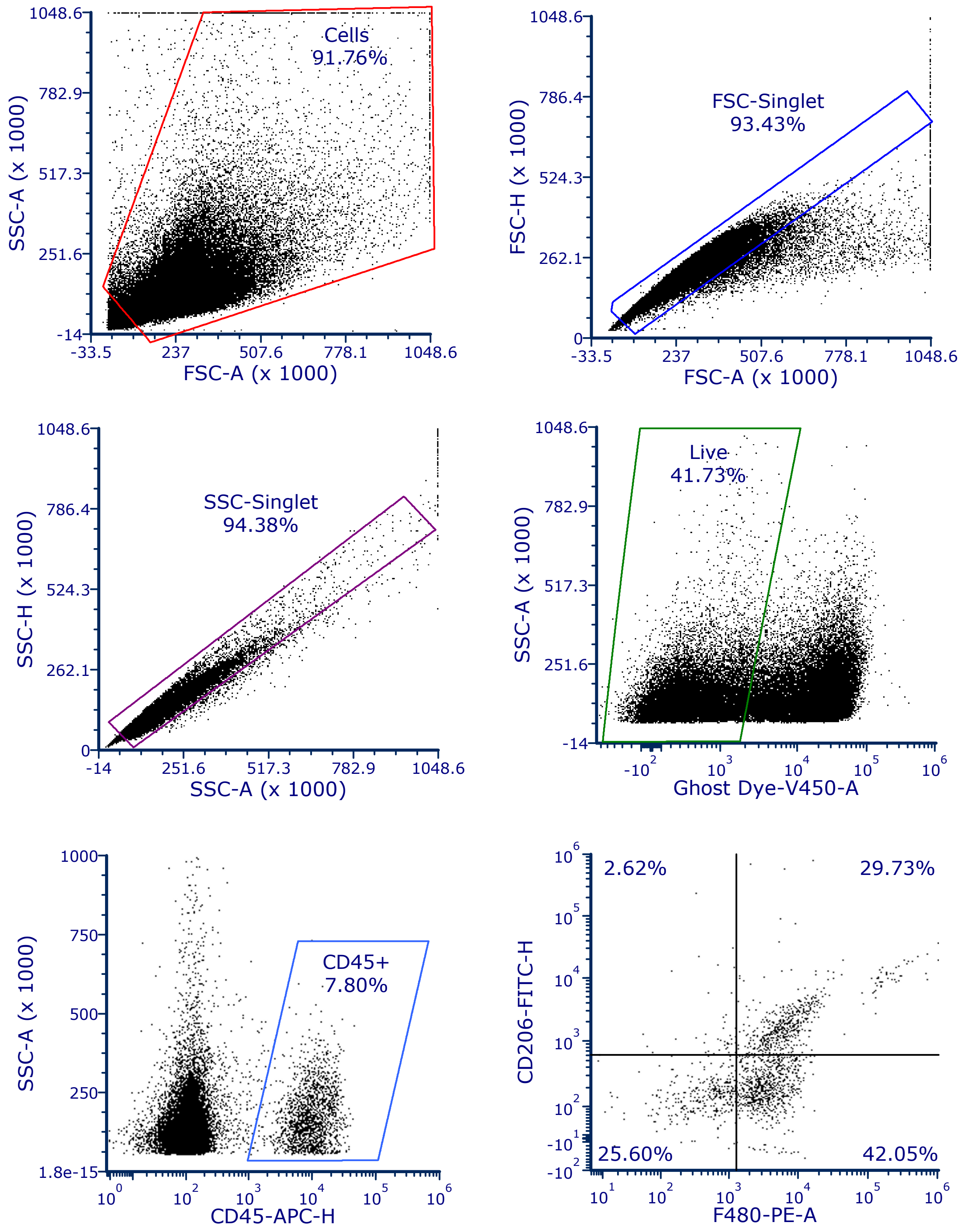


**Figure S8:** Flow cytometry was performed on single cell preparations of pooled whole mouse urethras and bladders (n=3 pooled samples/group). Live cells were assessed for CD45 expression. CD45+ cells were assessed for F4/80 and CD206 expression. Representative data from one pooled urethra sample is shown.

Figure S9

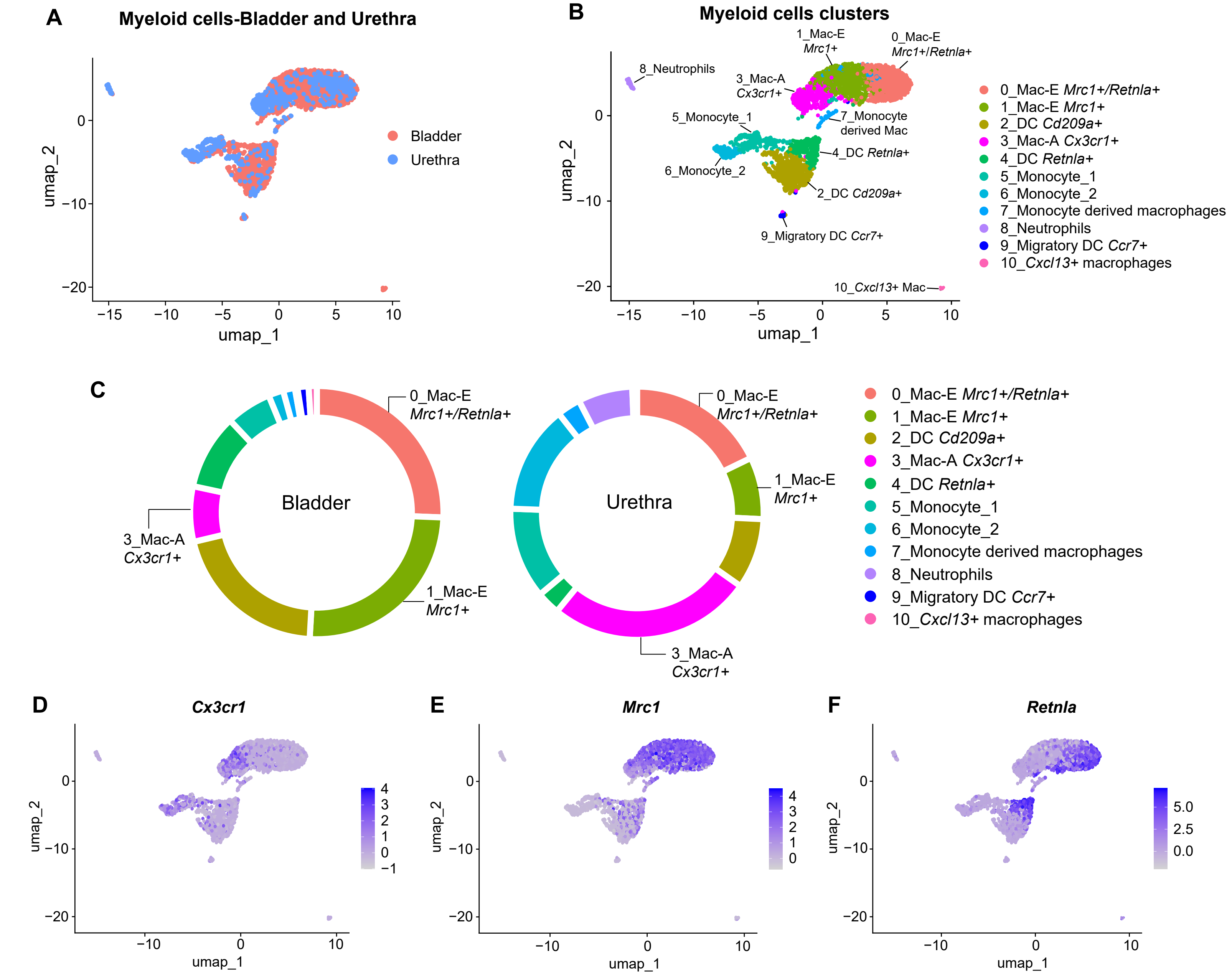


G

| | Top marker genes p-adj (lowest to highest) log2 fold change > 0.5 |
|---|---|
| Mac-E *Mrc1* +, *Retnla* + | *Fcna, Cd209f, Folr2, Ednrb, Selenop, F13a1, C4b, Retnla, Cd209g, Lyve1, Clec10a, Gas6, Ccl24, Ctsb, Mrc1, Igfbp4, Pltp, Fcgrt, Cfh, Maf* |
| Mac-E *Mrc1* + | *Pf4, C1qc, Jun, C1qb, Ccl2, Mmp9, Ccl7, Cbr2, Apoe, Selenop, Itm2b, C1qa, Csf1r, Fos, Egr1, Ccl12, Fosb, Mrc1, Ftl1, mt-Atp8* |
| Mac-A *Cx3cr1+* | *Pmepa1, Dcstamp, Cadm1, Zmynd15, Tafa2, Creb5, Frmpd4, Rnf180, Apbb2, Rgs1, Col14a1, Itga9, Il1a, Dst, H2-Ea, Fmnl2, Cxcl16, Myo1e, Arhgap22, Snhg15, Plcl1, Ms4a7, Cx3cr1* |

**Figure S9:** (A) Data from a published single cell RNA-sequencing data set of immune cells from young 3-month old mouse bladders (GSE149571) was integrated with immune cells subsetted from adult female mouse bladders (this study). Myeloid cells (non-lymphoid) were subsetted and labeled by tissue type. (B) Myeloid cells from bladders and urethras were clustered and identities applied to each cluster based on differentially expressed marker genes. (C) Distribution of myeloid cells by tissue type. Feature plots showing expression of (D) *Cx3cr1* (E) *Mrc1* and (F) *Retnla*. (G) Top marker genes for Mac-E and Mac-A clusters sorted by p-adjusted lowest to highest (cut-off p-adj < 0.05, log2FC > 0.5).

Figure S10

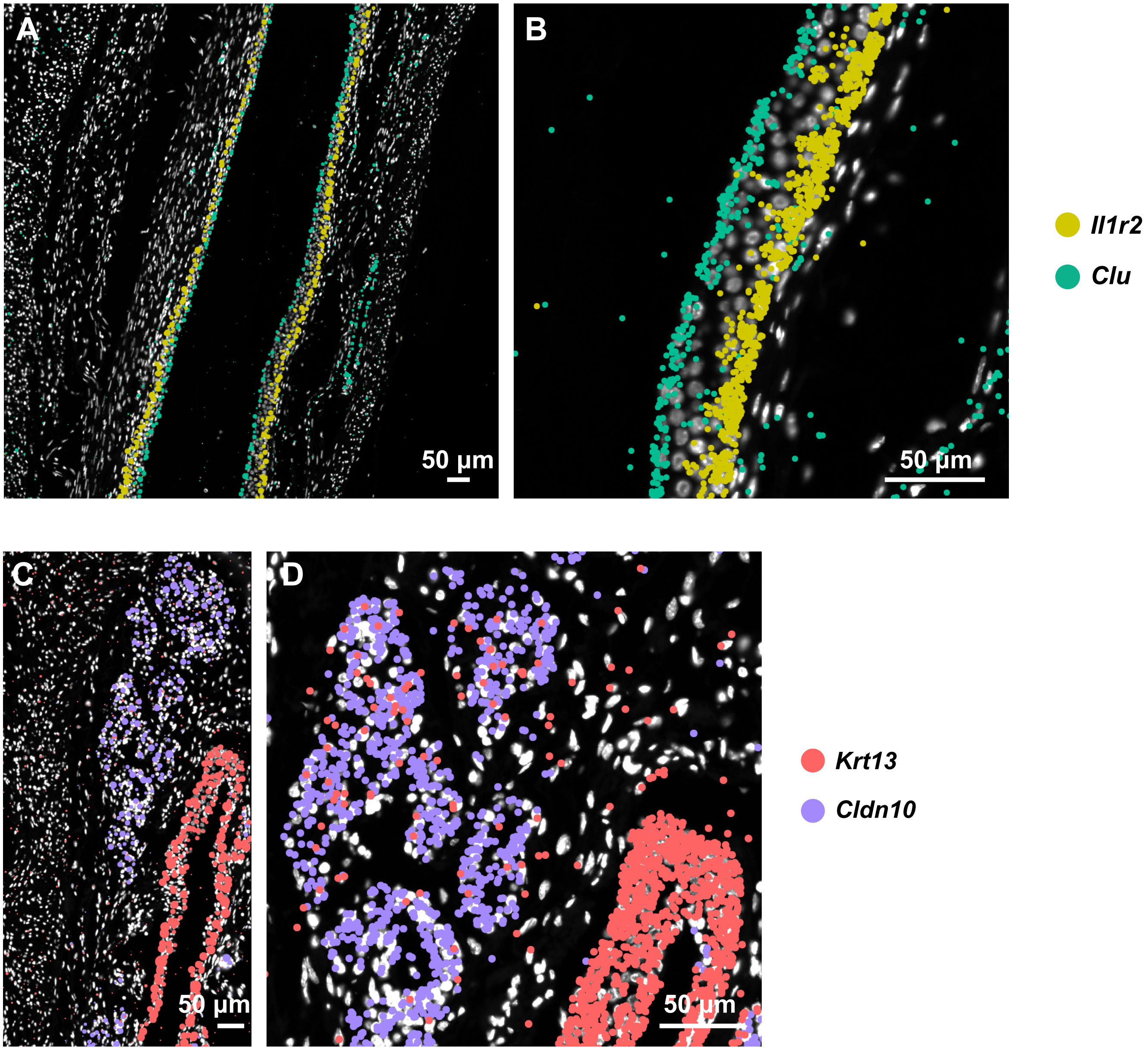


**Figure S10:** (A-B) Xenium expression data from the adult female mouse urethra showing localization of probes against the luminal epithelial marker gene *Clu* and the basal epithelial marker gene *Il1r2*. (C-D) Xenium expression data from the adult female mouse urethra showing localization of probes against the urethral gland marker gene *Cldn10* and the intermediate epithelial marker gene *Krt13*. Scale bar represents 50 microns.

Figure S11

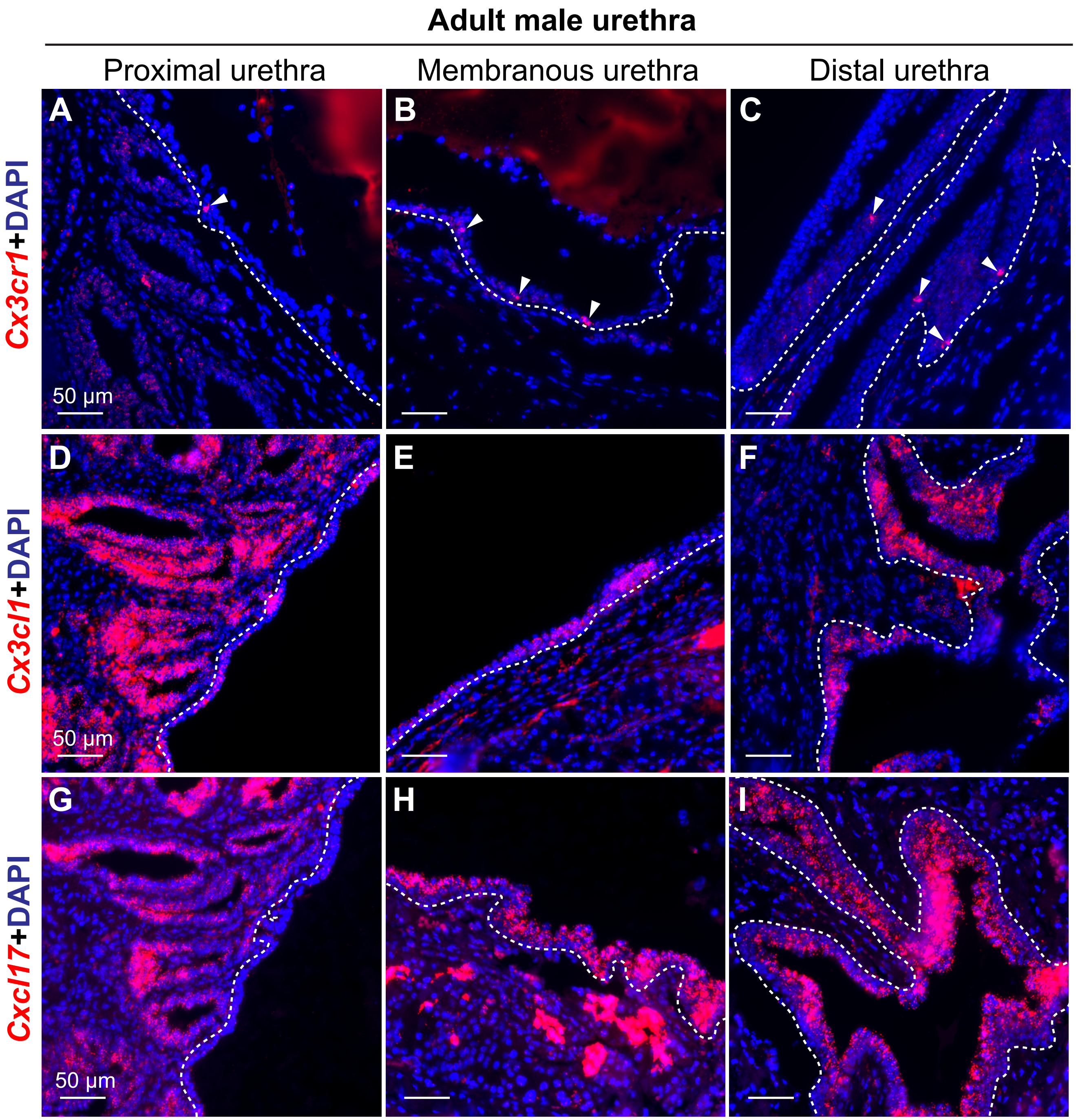


**Figure S11:** (A-C) Tissue sections of the adult male mouse urethra labeled with probes against *Cx3cr1* mRNA (in red). White arrowheads indicate *Cx3cr1*+ cells in the epithelial compartment (D-F) Tissue sections of the adult male mouse urethra labeled with probes against *Cx3cl1* mRNA (in red). (G-I) Tissue sections of the adult male mouse urethra labeled with probes against *Cxcl17* mRNA (in red). Images representative of n=3 mice/group. Nuclei are labeled in blue. White scale bar represents 50 microns. White dotted line represents border between epithelium and stroma.

Figure S12

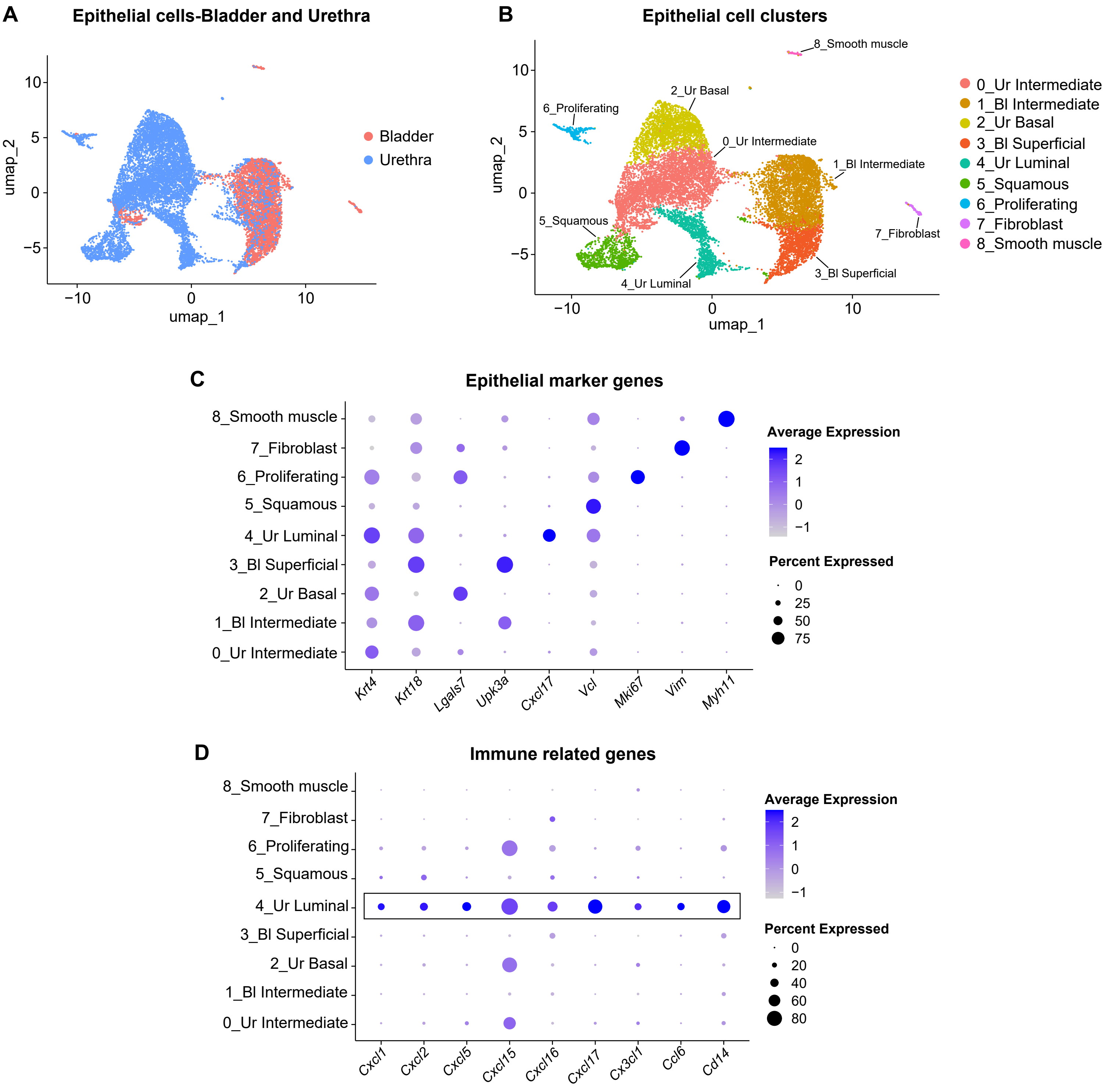


**Figure S12:** (A) A published dataset of single cell RNA-sequencing from wildtype mouse bladders (GSE129845) was integrated with female mouse urethra data (this study). Epithelial cells were subsetted and reclustered. Umap plot shows epithelial cells labeled by tissue type. (B) Epithelial cells from bladders and urethras were clustered and represented on a Umap plot. Cell type identity was applied to each cluster based on differentially expressed marker genes. (C) Dot plot showing marker gene expression for each cluster. (D) Dot plot showing expression of immune-related genes in the epithelial cell clusters.
