# Supplementary material for: Single cell map of the adult female mouse urethra reveals epithelial and stromal macrophages with distinct functional identities
Source: Mucosal Immunol. Author manuscript; Available in PMC 2025 Sep 26. (PMC7618168; doi:10.1016/j.mucimm.2025.09.001)
Supplement: Supplementary table descriptions [file EMS208696-supplement-Supplementary_table_descriptions.docx]

**Supplementary Tables:**

Table S1: Marker genes from all clusters obtained from single cell RNA-sequencing of the adult female mouse urethra.

Table S2: Marker genes from clusters obtained from single cell RNA-sequencing of the adult female mouse urethra post filtering of stressed cells.

Table S3: Marker genes from immune cell clusters obtained from single cell RNA-sequencing of the adult female mouse urethra.

Table S4: Marker genes from myeloid immune cell clusters obtained from single cell RNA-sequencing of the adult female mouse urethra.

Table S5: Marker genes obtained from comparison of Mac-A and Mac-E cell clusters.

Table S6: KEGG pathways enriched in Mac-Activated compared to Mac-Endocytic cell clusters.

Table S7: KEGG pathways enriched in Mac-Endocytic compared to Mac-Activated cell clusters.

Table S8: Marker genes from immune cell clusters obtained from single cell RNA-sequencing of the adult male mouse prostatic urethra.

Table S9: Marker genes from all clusters obtained from single cell RNA-sequencing of epithelial and stromal separations from the adult female mouse urethra.

Table S10: Marker genes from immune cell clusters obtained from single cell RNA-sequencing of epithelial and stromal separations from the adult female mouse urethra.

Table S11: Immune cell counts by cluster in epithelial and stromal separations from the adult female mouse urethra.

Table S12: Marker genes of cell clusters obtained from single cell RNA-sequencing of PBS or LPS instilled adult female mouse urethras.

Table S13: Gene ontology analysis from genes enriched in Mac-Endocytic compared to Mac-Activated cell clusters.

Table S14: Marker genes from myeloid immune cell clusters obtained from single cell RNA-sequencing integrated dataset of bladder and urethra immune cells.

Table S15: Marker genes from epithelial cell clusters obtained from single cell RNA-sequencing of the adult female mouse urethra.

Table S16: Marker genes from epithelial cell clusters obtained from single cell RNA-sequencing integrated dataset of adult mouse bladder and adult female mouse urethra.
